# Cyclo‐Polyproline: Chameleonic All‐Peptide Macrocycles With Induced‐Fit Host‐Guest Recognition

**DOI:** 10.1002/anie.8698780

**Published:** 2026-05-14

**Authors:** Camilla Di Girolamo, Patricia C. Fleming, Caroline R. Kwawu, Amanda R. Guimarães, Jimmy Muldoon, Julia Bruno‐Colmenarez, Yannick Ortin, Michael R. Probert, Felipe Fantuzzi, Aniello Palma

**Affiliations:** ^1^ School of Chemistry University College Dublin Dublin Ireland; ^2^ Supramolecular and Interfacial Chemistry, School of Natural Sciences University of Kent Canterbury UK; ^3^ Department of Chemistry Kwame Nkrumah University of Science and Technology Kumasi Ghana; ^4^ Chemistry – School of Natural and Environmental Sciences Newcastle University Newcastle upon Tyne UK

**Keywords:** cyclo‐polyproline, host‐guest systems, peptide‐based macrocycles, peptide‐based supramolecular chemistry, supramolecular chemistry

## Abstract

We report the design, synthesis, and characterization of a novel class of all‐peptide macrocycles, Cyclo‐Polyprolines (**CP**). Exploiting the precision of Fmoc‐based solid‐phase peptide synthesis (SPPS) and head‐to‐tail macrocyclization, this platform grants unparalleled control over the macrocycle's primary sequence and secondary structure, offering a viable route toward *exo*‐/*endo*‐functionalization and addressing a bottleneck of traditional synthetic host macrocycles. The resulting **CP** scaffold is highly amphiphilic, exhibiting excellent solubility in both organic and aqueous media. Structural analysis via NMR spectroscopy and single‐crystal x‐ray diffraction reveals a distinct chameleonic character: the macrocycle shifts from an all‐junctions‐*cis* conformation in organic solvents to a predominantly all‐junctions‐*trans* isomer in water. We demonstrate that this transition is driven by a cooperative hydration effect, wherein water molecules stabilize the expanded framework through precise two‐point hydrogen bonding. Demonstrating responsive host‐guest capabilities, **CP** undergoes induced‐fit isomerization to bind ligands, successfully forming, among other species, an all‐peptide pseudo‐rotaxane. This methodology establishes a robust platform for creating functionalized, proline‐based hosts with significant potential in medicinal chemistry, drug delivery, and organocatalysis, thereby bridging the gap between supramolecular systems and enzyme mimetics.

## Graphical abstract

Macrocycles served as the genesis of supramolecular chemistry, advancing synthetic, separation, and materials fields. Despite their utility, they typically lack synthetic control. This work establishes a robust platform for all‐peptide macrocycles capable of host‐guest complexation and chameleonic behavior. Bridging supramolecular systems and enzyme mimetics, they offer significant potential in medicine, drug delivery, and organocatalysis.

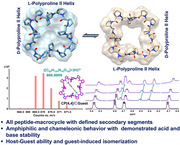

## 1 Introduction

**FIGURE 1:**
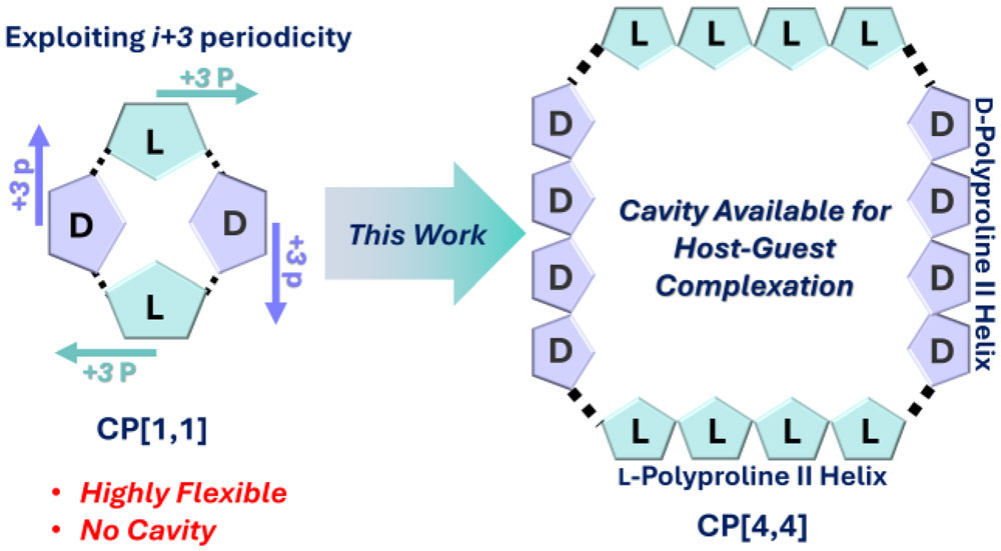
**CP[1,1]** lacks a cavity and is highly flexible. This work exploits polyproline periodicity; each side of **CP[1,1]** is extended by tetra‐(D/L)‐proline helical segments to yield **CP[4,4]**, which possesses a cavity available for host‐guest complexation.

Inspired by nature, chemists have long pursued the synthesis of supramolecular constructs that emulate the function of natural catalytic systems, such as enzymes [1]. With the emergence of the first macrocyclic molecules capable of performing host‐guest chemistry in the 1970s, the concept of enzyme mimetics was introduced. This arose from the resemblance between the confined cavities of these macrocyclic systems and enzymatic active sites. Prominent examples include cyclodextrins (CDs), cucurbiturils (CBs), calixarenes (CXs), and pillar[*n*]arenes. The ability of these synthetic systems to perform host‐guest chemistry has been exploited in a plethora of research fields, ranging from biological applications (e.g., drug delivery) and materials science (e.g., supramolecular materials) to chemical transformations [2, 3, 4, 5, 6, 7, 8]. Despite their potential, these classical macrocycles exhibit some limitations. For example, native calixarenes and pillar[n]arenes are entirely insoluble in water [9]. Similarly, cyclodextrins and cucurbiturils with cavity sizes suitable for pharmaceutical formulations (i.e., β‐CD and CB[8]) exhibit poor water solubility compared to their smaller homologs [7, 10]. While the water solubility issue has been addressed in some cases by introducing hydrophilic functional groups onto the polyaromatic rims [11], a broader challenge remains: these macrocycles are typically synthesized via condensation chemistry or enzymatic transformations, approaches that inherently limit the achievable structural complexity [10, 12, 13, 14, 15]. Functionalization is largely restricted to the rims or surfaces exposed to the bulk solvent (i.e., *exo*‐functionalization). However, some macrocycles are extremely difficult to *exo*‐functionalize in a controlled manner, such as pillar[n]arenes and CB[n] [16, 17]. With the goal of enhancing chemical diversity, modular syntheses for ABCD‐type heterocalix[4]arenes have been successfully reported. However, these solution‐phase routes require laborious and time‐intensive purification steps [18, 19]. Moreover, with the exception of a very limited number of examples, it is extremely difficult to achieve inner cavity functionalization (i.e., endo‐functionalization) in a controlled and modular manner [20, 21]. Our approach aims to address these synthetic shortcomings. Herein, we propose the use of a specific class of secondary structured peptides, namely polyproline helices, to access a new class of all‐peptide macrocycles. Proline‐rich peptides are characterized by a stable and resilient secondary structure known as the polyproline helix. This type of biopolymer has a rigid, rod‐like structure that can interconvert between two different secondary conformations (polyproline II helix and polyproline I helix) as a function of the environment to which it is exposed (i.e., temperature, solvent polarity, and pH) [22, 23, 24]. Both polyproline II and I helices have a structural periodicity of ≈*i* + 3 amino acids. In contrast to other helices used in supramolecular chemistry, we have shown that the polyproline helix is retained even in exceptionally short sequences (as few as four prolines), enabling the design of bioinspired responsive materials [25, 26]. These helices also possess a C_3_ rotational symmetry axis. As such, they present three faces, which can be accurately functionalized to induce the desired controllable self‐assembly [27, 28, 29]. We have recently reported the use of polyproline helices as unique supramolecular bioinspired building blocks for the formation of responsive supramolecular peptide frameworks [25, 26], peptide‐based Pd cages [30], and metallo‐peptide nanoparticles [29]. In addition to our work, other groups have utilized polyproline helices to construct complex supramolecular architectures [22, 27, 31, 32, 33, 34, 35, 36, 37]. In particular, Lewandowski and Wennemers have recently described the use of proline‐based programmable templates that clearly demonstrate the potential of polyproline helices for *endo*‐functionalization [38]. Although these architectures are fundamentally distinct from the one reported herein, the efficiency of utilizing proline helix topography for *endo*‐functionalization will translate effectively to our system. Our inspiration is drawn from the extensive literature on cyclic tetrapeptides containing alternating L‐ and D‐amino acids [39, 40, 41, 42, 43, 44, 45, 46] and, in particular, the reported cyclic tetrapeptide comprised of alternating L‐ and D‐prolines, which are known to induce a turn at the junction [47]. Since the cyclic tetrapeptide is highly flexible and its cavity too small for guest binding, we propose exploiting the *i* + 3 periodicity of polyproline helices to elongate each side to four units (Figure 1).

Our strategy aims to obtain a cyclic polyproline via a head‐to‐tail intramolecular macrocyclization reaction of the linear peptide analog. The linear hexadecameric peptide is synthesized using well‐established Fmoc‐based solid‐phase peptide synthesis (SPPS). This approach simplifies purification at each synthetic step and allows an unparalleled level of control over the primary sequence of the macrocycle. The well‐known secondary‐structure stability of polyproline tetramers, in turn, enables the introduction of functional groups with highly desirable positional control. Therefore, this work reports a robust methodology for accessing a novel class of all‐peptide macrocycles, Cyclo‐Polyprolines[4,4] (**CP[4,4**]). Our strategy utilizes controlled step‐growth solid‐phase synthesis to resolve long‐standing challenges in positional control, offering a facile and highly modular synthetic pathway. We show that **CP[4,4]** is amphiphilic and highly soluble in both organic and aqueous solvents. Using NMR spectroscopy and SC‐XRD, we unequivocally establish that **CP[4,4]** possesses a chameleonic nature, switching conformations based on solvent polarity. This behavior is associated with the ability of peptides to cross cell membranes [48], underscoring the potential of this class of peptide‐based macrocycles in medicinal chemistry and drug delivery. Finally, we demonstrate that **CP[4,4]** facilitates host‐guest interactions in diverse media, undergoing guest‐induced conformational change reminiscent of the induced‐fit mechanism observed in enzymes. This work establishes a clear route to highly *exo*‐/*endo*‐functionalized proline‐based macrocycles as responsive supramolecular hosts and potential organocatalysts, thereby bridging the gap between supramolecular systems and enzyme mimetics.

## 2 Results and Discussion

**SCHEME 1:**
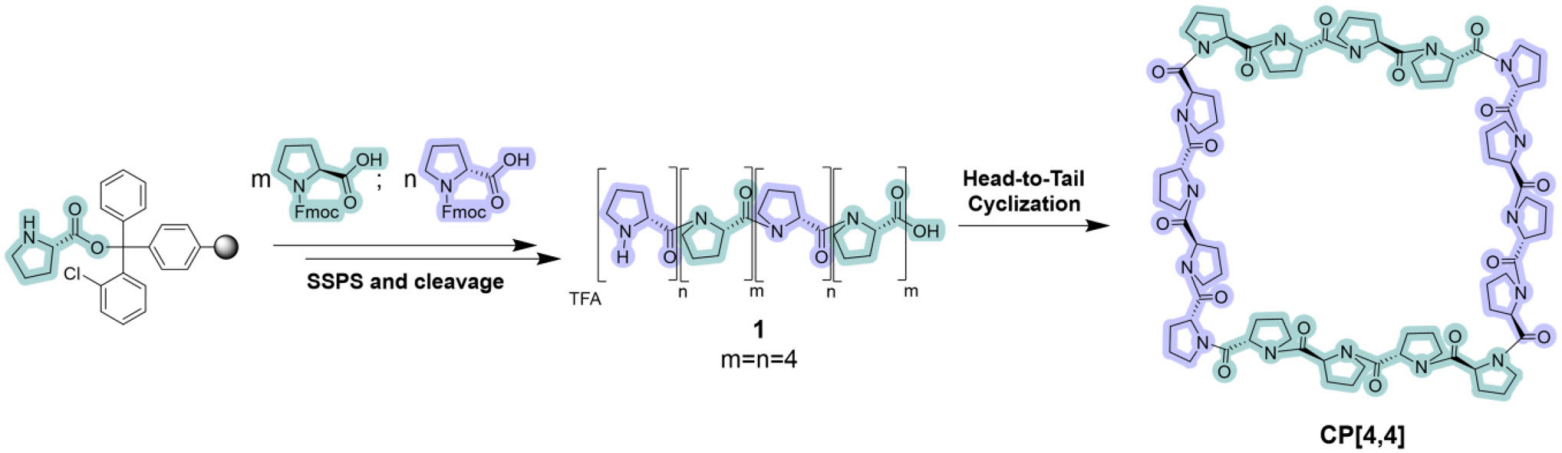
Schematic representation of the solid‐phase peptide synthesis (SPPS) used for the synthesis of the linear peptide **1** and of the head‐to‐tail cyclization of **CP[4,4]**. Peptide **1**, dissolved in acetonitrile (2.15 mM), was added using a syringe pump (rate 3 mL/h) to a stirring solution of PyBOP and DIPEA in acetonitrile (9.5 mM) at 60°C. Upon purification on reverse‐phase preparative HPLC, **CP[4,4]** was obtained in 43% yield over two synthetic steps (ESI, Section 2).

The linear peptide **1** was synthesized on an automated peptide synthesizer utilizing Fmoc SPPS techniques in quantitative yield (Scheme 1; for peptide method optimization, see ESI, Section 2). This approach enabled precise, atom‐level control over the primary sequence and streamlined the removal of excess reagents and by‐products after each step, presenting a clear advantage over other methods for generating macrocyclic structures reported in the literature. Upon cleavage from the resin followed by precipitation and washing with cold diethyl ether, the crude product was obtained in high purity, as confirmed by LC‐HRMS, and used in the next step without further purification.

^1^H‐NMR analysis of **1** showed the presence of a limited number of conformations in solution, consistent with the expected retention of the polyproline helical secondary structure across all the tetrameric segments (ESI, Section 3). The synthesis of the novel cyclic polyproline macromolecule was then completed via a head‐to‐tail intramolecular cyclization. To minimize the formation of oligomers, the linear starting material was added slowly to a round‐bottom flask containing the coupling reagent and base. The use of *N*,*N*‐DIC and Oxyma Pure as coupling reagents did not yield the desired product but instead led to oligomeric material (Figure S33). The more reactive coupling reagent PyBOP was therefore used for the cyclization of **1**, giving promising results. After optimization of the macrocyclization reaction conditions (Table S1), the novel cyclo‐polyproline (**CP**) was obtained in excellent crude yield. Since this macrocycle consists of alternating tetramers of L‐ and D‐proline residues, it is named **CP[4,4]**, where the indices denote the length of the L‐ and D‐segments. Purification via reverse‐phase preparative HPLC afforded the desired pure product in an impressive 43% yield over two synthetic steps, as confirmed by LC‐HRMS analysis.

### 2.1 Solution and Solid‐State Characterization of **CP[4,4]**

**FIGURE 2:**
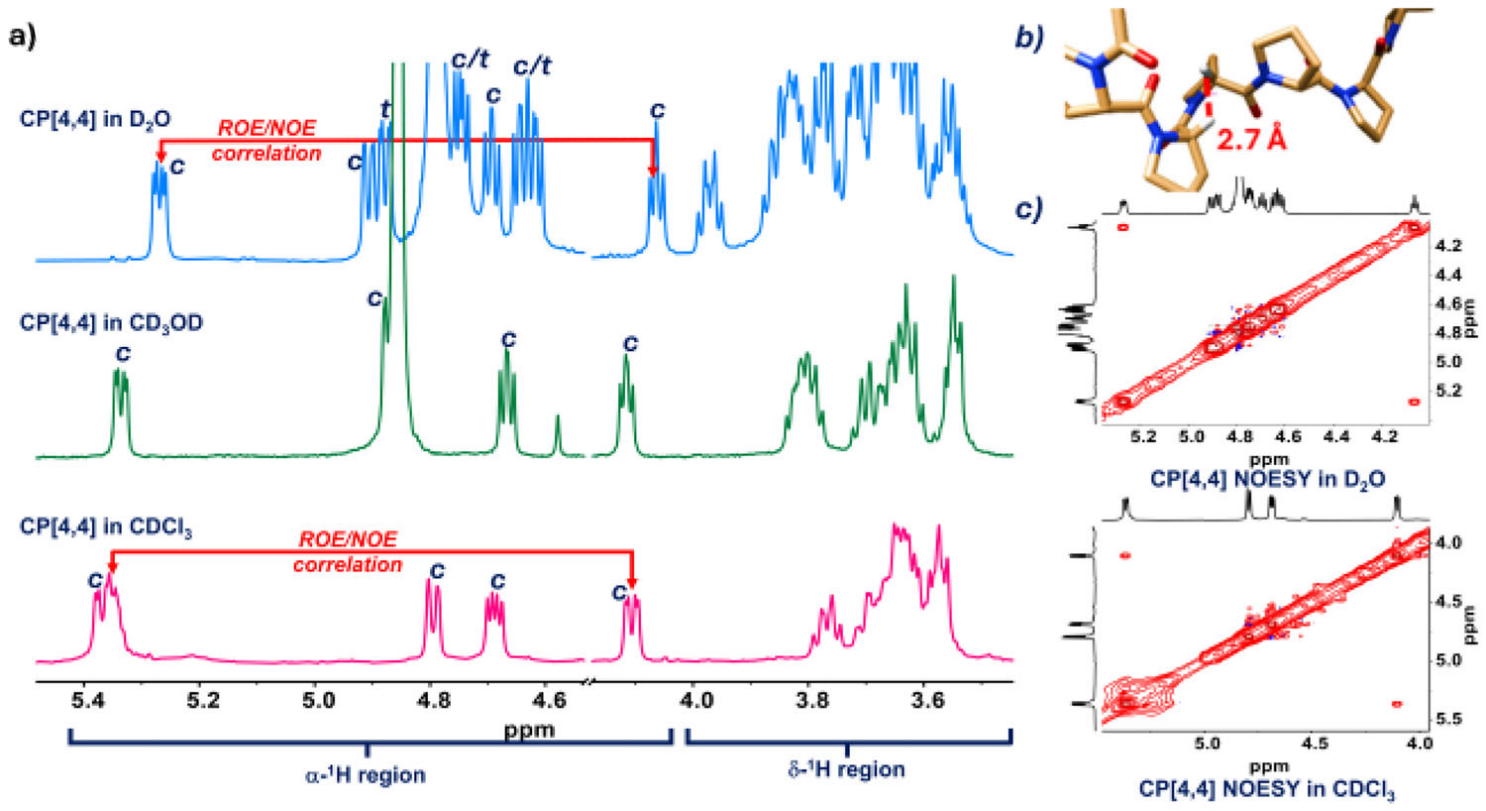
(a) ^1^H‐NMR of **CP[4,4]** in CDCl_3_, CD_3_OD, and D_2_O. Only the all‐junctions‐*cis* isomer is observed in CDCl_3_ and CD_3_OD, whereas a mixture of all‐junctions‐*cis* and all‐junctions‐*trans* is observed in D_2_O. All α‐protons of **CP[4,4]** are marked with ***c*** for the all‐junctions‐*cis* conformation or ***t*** for the all‐junctions‐*trans* conformation. ROE/NOE correlations between the L/D junction points are highlighted; (b) SC‐XRD of all‐junctions‐*cis* **CP[4,4]** highlighting the distance between the two α‐protons at the L‐ and D‐proline junctions; (c) zoomed‐in region of the **CP[4,4]** NOESY spectrum in D_2_O and CDCl_3_.

Based on the report focusing on the cyclic D‐L‐D‐L‐polyproline (i.e., **CP[1,1]** according to our notation), which highlighted the presence of *cis*/*trans* isomerization across its four amide bonds [47], we investigated the conformational behavior of **CP[4,4]** in different solvents using ^1^H‐NMR (Figure 2). **CP[4,4]** was dissolved in D_2_O, CDCl_3_, and CD_3_OD to give three 2.25 mM solutions for NMR analysis. Remarkably, **CP[4,4]** showed good solubility at room temperature in water, a polar protic organic solvent (methanol), and a nonpolar organic solvent (chloroform). This contrasts with many native macrocycles.

**FIGURE 3:**
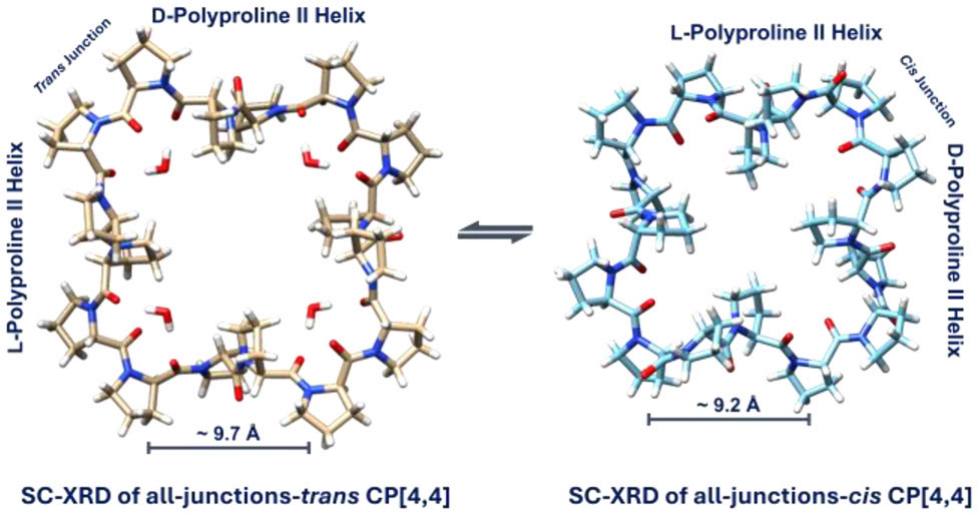
SC‐XRD structure of all‐junctions‐*cis* **CP[4,4]** (CCDC 2532549) obtained from slow evaporation of a CDCl_3_ solution, and SC‐XRD structure of all‐junctions‐*trans* **CP[4,4]** (CCDC 2534359) obtained from slow evaporation of a D_2_O solution. The two conformations exist in equilibrium.

^1^H‐NMR analysis of **CP[4,4]** in D_2_O revealed the presence of two major isomers (Figure 2), each displaying a relatively simple spectrum, suggesting a high degree of symmetry in both structures. This observation indicates that not all amide bonds are isomerizing in solution, in contrast to what was reported for **CP[1,1]** [47]. We hypothesized that, in both isomers present in solution, the tetrameric polyproline segments adopt the stable polyproline II helix secondary structure, while *cis/trans* amide isomerization occurs exclusively at the four L/D junction points. Multiplicity‐edited HSQC was used to identify the α‐protons in **CP[4,4]** (ESI, Section 3). Interestingly, a through‐space correlation in both NOESY and ROESY between the α‐protons at 4.06 ppm and 5.27 ppm was observed for the minor isomer in D_2_O (Figure 2c and ESI, Section 3). Such a correlation between the α‐protons at the L/D junction points is observable only when the L‐Pro and D‐Pro amide bonds are in the *cis* conformation (Figure 2b). We therefore assign this species as the all‐junctions‐*cis* **CP[4,4]** (Figure 3). Accordingly, in D_2_O **CP[4,4]** exists in equilibrium between the all‐junctions‐*trans (*∼60%) and all‐junctions‐*cis* (∼40%) isomers (Figure S23). Variable‐temperature ^1^H‐NMR was performed on CP[4,4] in D_2_O to estimate the rate of isomerization. The sample was heated to 90°C in 10°C increments. The resulting spectra exhibited minimal changes, and the peaks corresponding to the all‐junctions‐*cis* and ‐*trans* isomers did not coalesce. This indicates that the activation energy is > 18 kcal/mol while demonstrating the thermal stability of **CP[4,4]** (Figure S32). We propose that interconversion is mediated by a 180° rotation of the amide bonds at the junctions between L‐ and D‐polyproline segments (ESI, Section 10). We were pleased to find that slow evaporation of the **CP[4,4]** D_2_O solution at room temperature yielded colorless block‐shaped crystals in the tetragonal space group *I* $\bar{4}$, suitable for single‐crystal x‐ray analysis. Compound **CP[4,4]** in D_2_O crystallized in the all‐junctions‐*trans* amide conformation at the four L‐ and D‐proline segment junctions (Figure 3).

Analysis of the CDCl_3_ sample of **CP[4,4]**, dried from the semipreparative purification, clearly indicated two isomers in solution (ESI, Section 9). Through‐space correlations between the α‐protons at 4.06 and 5.27 ppm confirm that the all‐junctions‐*cis* isomer is the major species in solution. Notably, within 72 h, the NMR sample in CDCl_3_ showed the appearance of crystalline material. Isolation and dissolution of these crystals in fresh CDCl_3_ yielded a ^1^H‐NMR spectrum corresponding exclusively to the all‐junctions‐*cis* isomer (Figure 2a). This result indicates that the all‐junctions‐*trans* **CP[4,4]** is equilibrating into the all‐junctions‐*cis* **CP[4,4]** in chloroform. To our delight, the crystals of **CP[4,4]** obtained from the chloroform solution were also suitable for single crystal x‐ray diffraction analysis. Compound **CP[4,4]** in CDCl_3_ crystallized in the tetragonal space group *I* $\bar{4}$. The diffraction data were used to unambiguously determine the full structure of all‐junctions‐*cis* **CP[4,4]**, although the chloroform present in the crystals occupies locations that only have weak interactions and therefore displays significant disorder in the final structural model. Consistent with the NMR data in CDCl_3_ and CD_3_OD, the solid‐state structure of all‐junctions‐*cis* **CP[4,4]** shows that all four junctions between L‐ and D‐proline tetramers adopt the *cis* amide conformation, with a distance between the α‐protons of the L‐ and D‐prolines of 2.7 Å (Figure 2b). Importantly, in both isomers, all‐junctions‐*trans* and all‐junctions‐*cis* **CP[4,4]**, the L‐ and D‐polyproline tetrameric segments retain the polyproline II helical conformation (Figure 3). These results have significant implications for the positional control of functional groups within this novel macrocyclic cavity. Finally, method development on the semipreparative HPLC for an aqueous sample of **CP[4,4]** allowed us to cleanly separate the two isomers (Δ*t* = 198 s; ESI, Section 9). However, reinjection of the individually collected fractions, handled without delay, yielded chromatograms in which both isomers reappeared, consistent with rapid equilibration of the two species in aqueous media. The stability and solution equilibrium of **CP[4,4]** were evaluated as a function of pH. **CP[4,4]** was dissolved in D_2_O/DCl (pH 0.8) and D_2_O/Na_2_CO_3_ (pH 11.6). In both samples, the ratio of *cis/trans* isomers remained unchanged. Impressively, after 2 weeks at room temperature, no signs of decomposition were observed for either sample (Figures S34 and S35).

### 2.2 Computational Study of **CP[4,4]** Isomer Stability

Analysis of the SC‐XRD for the two isomers of **CP[4,4]** gives us a good understanding of their stability and interconversion mechanism. In the crystals obtained from water, the all‐junctions‐*trans* isomer binds four water molecules in well‐defined pockets within the macrocycle (Figure 3). Each pocket orientates a water molecule so that it can engage in two‐point hydrogen bonding with amide carbonyls from alternating proline residues. This favorable arrangement is not accessible in the all‐junctions‐*cis* framework, where water can at best interact with carbonyl groups on vicinal prolines in a less favorable geometry. To rationalize these observations, we carried out quantum‐chemical calculations on both the isolated macrocycles and their hydrated complexes. Geometry optimizations were carried out at the GFN2‐xTB level [49], followed by single‐point energy calculations at the PBE0‐D3(BJ)/def2‐TZVPP level [50, 51, 52, 53, 54]. Solvation effects were included using the CPCM/SMD implicit solvent [55, 56, 57] for water and chloroform. Further computational details are provided in the ESI. Focusing first on the macrocycles without explicit water, the all‐junctions‐*cis* isomer is more stable than the all‐junctions‐*trans* by 8.7 kcal mol^−1^ in the gas phase, 8.0 kcal mol^−1^ in chloroform, and 7.2 kcal mol^−1^ in water. Thus, increasing polarity modestly stabilizes the all‐junctions‐*trans* geometry and is sufficient to rationalize the all‐junctions‐*cis* preference in chloroform, but this effect alone cannot account for the predominance of the all‐junctions‐*trans* form in aqueous solution. Because the SC‐XRD structure of the all‐junctions‐*trans* isomer shows four well‐defined water molecules hydrogen‐bonded to the macrocycle, we next examined the effect of explicit solvation. Starting from the crystallographically observed all‐junctions‐*trans* **CP[4,4]** tetra‐hydrated complex, we optimized structures containing one, two, three, and four water molecules and compared these with the corresponding all‐junctions‐*cis* **CP[4,4]** *n*‐hydrate complexes (*n* = 1–4). In the all‐junctions‐*trans* macrocycle, the carbonyl groups at each corner define well‐oriented pockets that allow each water molecule to form two strong hydrogen bonds to alternating amide carbonyl groups. By contrast, the geometry of the all‐junctions‐*cis* isomer enforces a twisted arrangement of carbonyls so that each water molecule can at best bridge vicinal carbonyl groups with less favorable distances and angles, thereby preventing the formation of the same two‐point hydrogen‐bonding motif. These structural differences are directly reflected in the computed stabilization energies. Coordination of a single water molecule (*n* = 1) to the all‐junctions‐*trans* **CP[4,4]** stabilizes this isomer by −23.2 kcal mol^−1^ via two‐point hydrogen bonding, in which both O‐H groups donate hydrogen bonds to two suitably oriented carbonyl oxygens. According to commonly used classifications of hydrogen‐bond strengths [58], weak, moderate, and strong hydrogen bonds typically span the ranges 1–4, 4–15, and 15–40 kcal mol^−1^, respectively. The average stabilization per O–H···O═C interaction in **CP[4,4]** (≈11.6 kcal mol^−1^) clearly falls within the moderate hydrogen‐bond regime. In contrast, interaction of the same water molecule with vicinal carbonyls in the all‐junctions‐*cis* **CP[4,4]** isomer affords a smaller stabilization of −13.8 kcal mol^−1^, approximately 9.4 kcal mol^−1^ less than in the all‐junctions‐*trans* case. Therefore, the monohydrate, all‐junctions‐*trans* **CP[4,4]** complex is already slightly more stable (by 0.7 kcal mol^−1^) than the corresponding all‐junctions‐*cis* **CP[4,4]** H_2_O species. Sequential addition of water molecules (*n* = 2–4) further increases the stability of the all‐junctions‐*trans* **CP[4,4]** macrocycle. Adsorption of the second, third, and fourth water molecules provides additional stabilization energies of −18.0 (to −41.2 kcal mol^−1^), −17.8 (to −59.0 kcal mol^−1^), and −18.3 kcal mol^−1^ (to a total of −77.3 kcal mol^−1^), respectively. These values reveal a clear cooperative effect: each water molecule is accommodated within a preorganized pocket and forms its own two‐point hydrogen‐bonding interaction with alternating carbonyl groups, reinforcing the stabilization imparted by those already bound. Thus, the preference for the all‐junctions‐*trans* **CP[4,4]** isomer in water arises from a combination of increased solvation polarity and the cumulative formation of several geometrically favorable hydrogen bonds within the macrocycle cavity and water molecules. Next, we performed molecular dynamics simulations to investigate the interaction of the proline residues with explicit water molecules (ESI, Section 10). The all‐junctions‐*trans* conformation was maintained throughout the 100 ns simulation, as confirmed by the RMSD profile (Figure S61). A recurring structural feature observed during the simulation is the presence of a single water molecule bridging two intercalated proline moieties via hydrogen bonding (Figure S59). This water‐mediated interaction is associated with an oxygen‐oxygen distance of approximately 4 Å between the two proline residues involved (Figure S60). The formation and stability of this motif are strongly influenced by key dihedral angles that define the pinch‐like architecture of the **CP[4,4]** macrocycle. Alterations in these dihedral angles disrupt the characteristic ∼4 Å oxygen‐oxygen separation, thereby preventing the formation of the bridging hydrogen‐bonded interaction (Figure S60). Analysis of the simulation trajectories shows that the ∼4 Å oxygen‐oxygen distance predominates in the majority of frames (Figure S62). In addition, a systematic decrease in the relevant dihedral angles relative to the initial structure is observed and maintained throughout the simulation (Figure S63). This conformational adjustment promotes a geometry that is more favorable for the formation and persistence of the water‐mediated hydrogen‐bonded motif. The experimental and computational analyses converge to a consistent structural interpretation: the isolated macrocycle favors the all‐junctions‐*cis* isomer in nonpolar media, whereas cooperative, preorganized water‐mediated hydrogen bonding stabilizes the all‐junctions‐*trans* form in aqueous solution. These results identify solvent‐dependent amide isomerization at the junctions as the structural switch underlying the conformational equilibrium of **CP[4,4]**.

### 2.3 Investigating the Host‐Guest Complexation and Chameleonic Behavior of **CP[4,4]**

**FIGURE 4:**
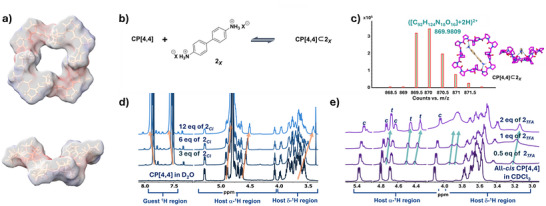
(a) Coulombic surface of all‐junctions‐*trans* **CP[4,4]** calculated in ChimeraX [59]; (b) Reaction scheme of the titration between **CP[4,4]** and benzidine salt **2** to form the complex **CP[4,4]⊂2*_X_*** (where **X^−^** is either trifluoroacetate or chloride salt of the protonated benzidine); (c) HRMS spectrum and isotopic pattern for the **CP[4,4]⊂2*_X_*** complex. HRMS: calcd for [M + 2H]^2+^: C_92_H_126_N_18_O_16_, 869.4794; found: [M + 2H]^2+^ 869.9809 (ESI, Sections 1 and 5). Red boxes overlaying the measured peaks denote the calculated isotopic pattern for the complex (bounds set to calculated isotopic abundances with a permitted mass deviation of ±5 ppm). Inset: top and later views of binding pose of **CP[4,4]⊂2*_X_*** obtained using AutoDock Vina [60, 61]; (d) ^1^H‐NMR titration of **2*_Cl_*** (1–12 equiv.) into a D_2_O solution of **CP[4,4]** (2.25 mM). Arrows highlight changes in the chemical shifts of diagnostic peaks; (e) ^1^H‐NMR titration of **2*_TFA_*** (0.5‐2 equiv.) into a CDCl_3_ solution of **CP[4,4]** (2.25 mM). Initially, only all‐junctions‐*cis* **CP[4,4]** is present; upon titration of the guest, the all‐junctions‐*trans* **CP[4,4]⊂2*_TFA_*** complex is observed. Arrows highlight the spectral changes.

The SC‐XRD analysis of the all‐junctions‐*trans* **CP[4,4]** clearly showed a saddle shape evident in its lateral view (Figure 4a). The macrocycle's cavity in the all‐junctions‐*trans* conformation can host solvent molecules, thereby demonstrating its potential to engage in host‐guest chemistry. Moreover, four water bridges are formed by four water molecules hydrogen‐bonding at the corners of **CP[4,4]**, which is crucial to stabilize the all‐junctions‐*trans* conformation.

The central cleft features two hydrogen‐bond acceptor corners positioned opposite each other. The walls of this cleft are primarily hydrophobic (Figure 4a). Analysis of this molecule using MoloVol gave a measurement of 97.6 Å^3^ for the accessible volume (ESI, Section 6) [62]. We decided to use molecular docking carried out with AutoDock Vina [60, 61] to qualitatively assess possible binding poses of potential ligands for **CP[4,4]** [63, 64]. Due to the topology of the complex, the first guest investigated in silico was the benzidine HCl salt, **2*_Cl_***, as this molecule has two H‐bond donor groups spaced by a flat aromatic unit. Docking studies for **CP[4,4]⊂2*_Cl_*** revealed a persistent binding pose in which **2*_Cl_*** resides within the macrocyclic cleft of **CP[4,4**], with the protonated amine groups oriented toward the hydrogen‐bond acceptor groups (inset, Figure 4c and ESI, Sections 5 and 6). Titration of a solution of **2*_Cl_*** in D_2_O into a stock solution of **CP[4,4]** in D_2_O (2.25 mM) showed diagnostic shifts in ^1^H‐NMR for both the all‐junctions‐*trans* isomer of **CP[4,4]** macrocycle and guest peaks indicative of host‐guest complexation in a fast exchange regime (Figure 4d and ESI, Section 4). The peaks corresponding to the α‐protons (4.89 and 4.63 ppm, Figure 4d) and the δ‐protons (final position at 3.39 ppm, Figure 4d) of the proline residues in **CP[4,4]** exhibit a clear downfield shift. In turn, β‐ and γ‐proton signals also show shifts; however, the complexity of this spectral region precludes definitive assignment for most of them. Notably, the isolated peak at 3.39 ppm (Figure 4d) arises from a pronounced downfield shift of a signal originally located within the *δ*‐proton region of **CP[4,4]**, between 4.10 and 3.50 ppm (Figure 4d). The binding affinity between **CP[4,4]** and **2*_Cl_*** was determined by ^1^H‐NMR titration by monitoring the perturbation of **CP[4,4]** signal at 4.63 ppm as a function of guest concentration (Figure 4d, 4.63 → 4.49 ppm). The data were fitted using a 1:1 host:guest binding model using BindFit v0.5 [65, 66, 67]. This model gave an excellent numerical fit, with an apparent association constant of 70.82% ± 3.99% M^−1^ (ESI, Section 7). It is important to note that only **CP[4,4]** in the all‐junctions‐*trans* conformation can engage in host‐guest chemistry and that, in water, this species is in equilibrium with the all‐junctions‐*cis* **CP[4,4]** isomer. The formation of the **CP[4,4]⊂2*_Cl_*** complex was also confirmed by LC‐HRMS (Figure 4c). As ligand **2*_Cl_*** binds efficiently only to the all‐junctions‐*trans* **CP[4,4]** isomer, we hypothesized that titrating the pure all‐junctions‐*cis* isomer in CDCl_3_ with an organic soluble salt of **2** (e.g., **2*_TFA_***) could induce a conformational change to the all‐junctions‐*trans* form, yielding the **CP[4,4]⊂2*_TFA_*** complex. This mechanism would mimic the induced‐fit behavior typically observed in biological systems [68, 69]. The TFA salt of **2** was prepared quantitatively, and a stock solution of **2*_TFA_*** in DMSO‐d_6_ was then prepared (ESI, Section 4). Upon addition of **2*_TFA_*** to a CDCl_3_ solution of all‐junctions‐*cis* **CP[4,4]** (2.25 mM), new resonances appeared at 4.70, 4.48, 4.37, 3.91, 3.85, and 3.17 ppm in the ^1^H NMR spectrum, which are diagnostic of the all‐junctions‐*trans* isomer. This result confirmed that titrating **2*_TFA_*** into a solution of all‐junctions‐*cis* **CP[4,4]** resulted in the formation of the desired **CP[4,4]⊂2*_TFA_*** complex via an induced‐fit isomerization mechanism (Figure 4e). The presence of two sets of peaks for all‐junctions‐*cis* **CP[4,4]** and **CP[4,4]⊂2*_TFA_*** allows us to estimate a binding affinity of ∼650 M^−1^ for this system (ESI, Section 4). This result is consistent with the molecular docking poses, which show the guest positioned across the two hydrogen‐bond acceptor corners and within the hydrophobic cleft. Remarkably, no complexation was observed when commercially available unprotonated guest **2** was used in this experiment (ESI, Section 4).

**FIGURE 5:**
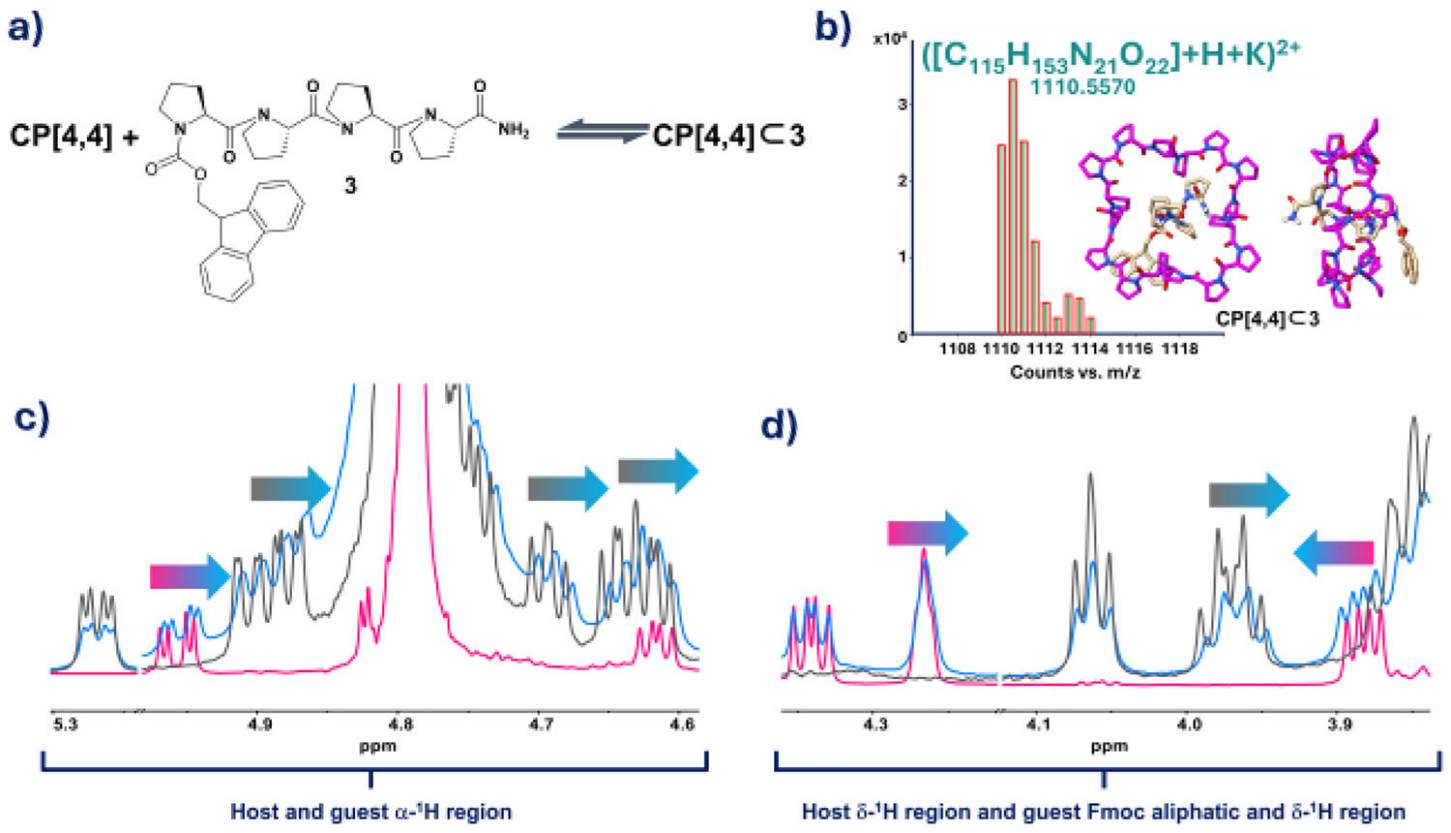
(a) Reaction scheme of the host‐guest interaction between **CP[4,4]** and polyproline four Fmoc **3** to form the complex **CP[4,4]⊂3**; (b) HRMS spectrum and isotopic pattern for the **CP[4,4]⊂3** complex; HRMS: calcd for [M + H + K]^2+^: C_115_H_154_N_21_O_22_K, 1110.5619; found; [M + H + K]^2+^: 1110.5570 (ESI, Sections 1 and 5). Red boxes overlaying the measured peaks denote the calculated isotopic pattern for the complex (bounds set to calculated isotopic abundances with a permitted mass deviation of ±5 ppm). Inset: top and lateral views of the binding pose of **CP[4,4]⊂3** obtained using AutoDock Vina [60, 61]; (c and d) ^1^H‐NMR regions overlap of **CP[4,4]** (gray trace; 2.37 mM in D_2_O), **3** (pink trace, 2.37 mM in D_2_O) and **CP[4,4]⊂3** obtained by mixing the host and guest in a 1:1 ratio (blue trace, 2.37 mM in D_2_O). Arrows highlight changes in the chemical shifts of diagnostic peaks.

Finally, we decided to investigate the ability of our system to tether a small peptide through its cavity. The peptide selected was the Fmoc‐capped polyproline tetramer **3** (Figure 5a) [25]. This peptide was conveniently selected, as we reported its SC‐XRD structure, which was used for molecular docking (ESI, Section 6). **3** has a polyproline II helical conformation and therefore a rod‐like secondary structure, which we hypothesized could tether within the **CP[4,4]** cavity. The **CP[4,4]⊂3** supramolecular complex is an all‐peptide pseudo‐rotaxane. Molecular docking confirmed that the tether peptide **3** could fit inside the **CP[4,4]** cavity. The low solubility of **3** in D_2_O, combined with its tendency to self‐assemble [25], posed an experimental challenge to performing a multipoint ^1^H‐NMR titration. A ^1^H‐NMR analysis of a 1:1 mixture of **CP[4,4]** and **3** showed small, diagnostic chemical shifts in the peaks of both the host and guest molecules, indicative of a fast‐exchange equilibrium (Figure 5c,d). These shifts in signals belonging to the **CP[4,4]** and the backbone of tether peptide **3** suggest the successful formation of an all‐peptide pseudo‐rotaxane. We were pleased to see that LC‐HRMS, followed by a mass‐mass spectra analysis of a solution of 2.30 mM in H_2_O of the **CP[4,4]⊂3** confirmed the presence of the desired complex (Figure 5b, ESI, Section 5).

## 3 Conclusions

In summary, a new peptide‐based macrocyclic structure, namely cyclo‐polyproline (**CP**), has been obtained in excellent yield via a head‐to‐tail intramolecular cyclization strategy of the linear peptide analog. The use of well‐established synthetic methodologies, such as solid‐phase peptide synthesis, enables a level of positional control when synthesizing this class of macrocycles, unmatched by other macrocyclization methodologies. **CP[4,4]** shows amphiphilic behavior with evidence of excellent solubility in water and organic solvents. **CP[4,4]** exists as a single all‐junctions‐*cis* isomer in organic solvents. However, in D_2_O, it is present as an equilibrium mixture of the all‐junctions‐*cis* and all‐junctions‐*trans* isomers. SC‐XRD analysis was conducted on both the all‐junctions‐*cis* and all‐junctions‐*trans* forms. DFT and MD investigations were subsequently performed to quantify the relative stability of the isomers and rationalize their solvent‐dependent conformational preferences. The ability of the all‐junctions‐*trans* **CP[4,4]** to perform host‐guest complexation was assessed first in silico using molecular docking and then experimentally. Selected ligands showed host‐guest complexation by means of ^1^H‐NMR and LC‐HRMS, successfully leading to the formation of an all‐peptide pseudo‐rotaxane. Remarkably, when the appropriate ligand was titrated into the all‐junctions‐*cis* **CP[4,4]** isomer, an induced‐fit isomerization and complexation were achieved, mimicking the response of more complex biological systems. Finally, the robust synthetic methodology and the well‐characterized attributes of this novel macrocycle in both solution and the solid state will facilitate future endeavors to develop this class of compound as supramolecular hosts for applications in separation and catalysis and will be reported in due course.

## Conflicts of Interest

The authors declare no conflicts of interest.

## Supporting information




**Supporting File 1**: CCDC 2534359 (all‐junctions‐*trans* **CP[4,4]**) and 2532549 (all‐junctions‐*cis* **CP[4,4]**) contain the supplementary crystallographic data for this paper. These data can be obtained free of charge from the Cambridge Crystallographic Data Centre via www.ccdc.cam.ac.uk/structures. ESI file can be found online. The following files are available free of charge. PDF of the experimental section. CIF and checkCIF files for the two **CP[4,4]** isomers. The authors have cited additional references within the Supporting Information [25, 57, 60, 63, 64, 65, 68].

**Supporting File 2**: anie72664‐sup‐0002‐Data.zip.

## Data Availability

The data that support the findings of this study are available on request from the corresponding author. The data are not publicly available due to privacy or ethical restrictions.
