# Supplementary material for: Cyclo‐Polyproline: Chameleonic All‐Peptide Macrocycles With Induced‐Fit Host‐Guest Recognition
Source: Angew Chem Int Ed Engl. 2026 May 14;65(27):e8698780. doi: 10.1002/anie.8698780 (PMC13327576; doi:10.1002/anie.8698780)
Supplement: Supplementary file 1 — Supporting File 1: CCDC 2534359 (all‐junctions‐trans CP[4,4]) and 2532549 (all‐junctions‐cis CP[4,4]) contain the supplementary crystallographic data for this paper. These data can be obtained free of charge from the Cambridge Crystallographic Data Centre via www.ccdc.cam.ac.uk/structures. ESI file can be found online. The following files are available free of charge. PDF of the experimental section. CIF and checkCIF files for the two CP[4,4] isomers. The authors have cited additional references within the Supporting Information [25, 57, 60, 63, 64, 65, 68]. [file ANIE-65-e8698780-s002.pdf]

*Supporting Information*

# Cyclo-Polyproline: Chameleonic All-Peptide Macrocycles with Induced-Fit Host-Guest Recognition

Camilla Di Girolamo,[a] Patricia C. Fleming,[a] Caroline R. Kwawu,[b,c] Amanda R. Guimarães,[b] Jimmy Muldoon,[a] Julia Bruno-Colmenarez,[a] Yannick Ortin,[a] Michael R. Probert,[d] Felipe Fantuzzi,[b] Aniello Palma*[a]

[a] School of Chemistry, University College Dublin, Belfield, Dublin 4, Ireland.

[b] Supramolecular and Interfacial Chemistry, School of Natural Sciences, Park Wood Rd, University of Kent, Canterbury CT2 7NH, UK.

[c] Department of Chemistry, Kwame Nkrumah University of Science and Technology, Kumasi, UPO, Ghana.

[d] Chemistry - School of Natural and Environmental Sciences, Bedson Building, Newcastle University, Newcastle upon Tyne, Tyne and Wear NE1 7RU, UK.

## Abstract

We report the design, synthesis and characterization of a novel class of all-peptide macrocycles, Cyclo-Polyprolines (**CP**). Exploiting the precision of Fmoc-based solid-phase peptide synthesis (SPPS) and head-to-tail macrocyclization, this platform grants unparalleled control over the macrocycle's primary sequence and secondary structure, offering a viable route towards exo-/endo-functionalization and addressing a bottleneck of traditional synthetic host macrocycles. The resulting **CP** scaffold is highly amphiphilic, exhibiting excellent solubility in both organic and aqueous media. Structural analysis *via* NMR spectroscopy and single-crystal X-ray diffraction reveals a distinct chameleonic character: the macrocycle shifts from an all-junctions-*cis* conformation in organic solvents to a predominantly all-junctions-*trans* isomer in water. We demonstrate that this transition is driven by a cooperative hydration effect, wherein water molecules stabilize the expanded framework through precise two-point hydrogen bonding. Demonstrating responsive host-guest capabilities, **CP** undergoes induced-fit isomerization to bind ligands, successfully forming, among other species, an all-peptide pseudo-rotaxane. This methodology establishes a robust platform for creating functionalized, proline-based hosts with significant potential in medicinal chemistry, drug delivery, and organocatalysis, thereby bridging the gap between supramolecular systems and enzyme mimetics.

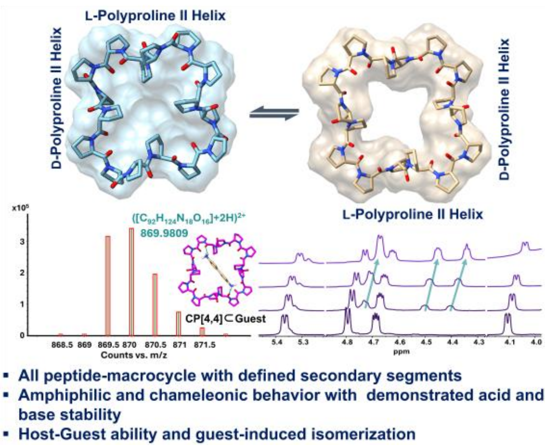

# Contents


1. Instruments and Methods ........ 3
2. Synthetic Methods ........ 5
3. NMR Spectroscopy Data ........ 11
4. NMR Titrations Data ........ 26
5. LC-HRMS Data ........ 34
6. Docking Studies ........ 36
7. Binding Affinity Studies ........ 39
8. Single Crystal X-Ray Diffraction Data ........ 42
9. HPLC Studies – Isomers Equilibration ........ 46
10. Molecular Dynamics and DFT Calculations ........ 48
11. References ........ 51

**Materials** - *L-Proline 2-chloro trityl resin (100-200 mesh, 0.45 mmol/g) 1% DVB was obtained from Merk (Novabiochem). Fmoc-L-proline, Fmoc-D-proline, N,N-Diisopropylethylamine (DIPEA), Trifluoroacetic acid (TFA), Oxyma Pure® and Diisopropylcarbodiimide (DIC) were obtained from Fluorochem Ltd. All the solvents used were procured from Fischer Scientific Ireland Limited. Acetic acid (AcOH) and Diethylamine ($Et_2NH$) were obtained from Merk. For all LC-HRMS and HPLC purpose, either HPLC or LCMS grade solvents were procured from Fischer Scientific and used as such. Deuterated methanol ($CD_3OD$) and chloroform ($CDCl_3$) were purchased from Merk. Deuterated water ($D_2O$) was obtained from Eurisotop.*

# 1. Instruments and Methods

**Automated Solid-Phase Peptide Synthesis:** The synthesis of the linear sequences was performed by a Liberty Blue Automated Microwave Peptide Synthesizer CEM.
**HPLC:** Semi Preparative HPLC was performed on a 1260 Infinity II (Agilent) HPLC, equipped with a C18 (SiliaChrom® Plus HPLC Column, 10 x 250 mm, 5 µm, 300 Å) at 1.9 mL/min flow rate monitored at 225/240/254 nm wavelengths.
**LC-MS:** Spectra were recorded on an Agilent 1260 HPLC system Single-Quadrupole MSD-XT, equipped with a C18 (SiliaChrom® Plus HPLC Column, 4.6 x 150 mm, 5 µm, 300 Å) at 0.4 mL/min flow rate monitored at 225/254 nm wavelengths. The ionization source is API-ES. Data was processed using MassHunter software.
**LC-HRMS:** Liquid Chromatography High-resolution Accurate Mass Spectra (LC-HRMS) data were acquired on an Agilent 6546 Quadrupole Time-of-flight MS system with an Agilent Jetstream Electrospray Ionization (ESI) source coupled to an Agilent Infinity Prime II HPLC system. Chromatography was effected by an Agilent Poroshell 50mm HPLC column, 2.7 µm particle size, having superficially porous particle, at 40ºC temperature. The gradient was 5min long, with mobile phase from 90% 0.1% formic Acid in water:10% acetonitrile to 10% formic acid in water:90% acetonitrile with an isocratic hold for further 5 minutes. MS spectra were acquired in positive mode, with a capillary voltage of 4000V, nozzle voltage of 2000V, fragmentor voltage 175V, skimmer voltage of 65V, drying gas temperature 325ºC, sheath gas 350ºC, nebulizer pressure 40 psi. Data were processed using Agilent MassHunter Qualitative Analysis software (version 10.0).

Linear peptidic sequences: Chromatography was effected *via* a 150mm SiliaChrom® Plus 5 µm C18 HPLC Column. The usual flow rate is 0.4 mL/min, the injection volume is 20 µL and the mobile phases of 0.1% formic Acid *vs* methanol on a gradient from 90% aqueous to 100% organic over 20min, with an isocratic hold of 3min at 100% methanol. API-ES Source conditions were, fragmentor voltage set to 110V, Drying Gas temperature of 350ºC and capillary voltage was 3000V.

Macrocyclic peptidic products: Purification was effected *via* a 250mm SiliaChrom® Plus 5 µm C18 HPLC Column. The usual flow rate is 1.9 mL/min, the injection volume is 20 µL and the mobile phases of 0.1% formic acid in water *vs* methanol on a gradient from 50% aqueous to 60% methanol over 12min, with an isocratic hold of 3min at 60% methanol and an isocratic hold at 100% methanol for 3min.

Host-Guest Complexes: Samples were directly infused from the LC inlet using a restriction capillary and mobile phases consisting of unbuffered water and ACN, with source conditions optimisations including fragmentor voltage set to 100V, Drying Gas temperature of 150C and Sheath Gas temperature of 150C. Capillary voltage was 4000V and nozzle voltage 2000V.

MS/MS of the **CP[4,4]⊂3** Complex: This measurement was carried out using targeted precursor isolation at 1102.065 Da and a wide precursor isolation window of 9 Da at a retention

time of 0.35min, at collision energies of 10, 20 and 40V with an MSMS range of 50-1700Da and a scan rate of 4 spectra/s. Source conditions were as previously stated, LC conditions incorporated a restriction capillary with an unbuffered isocratic Aqueous/MeOH mix at 1:1 ratio.

**NMR spectroscopy:** Nuclear magnetic resonance (NMR) spectra were recorded on a Varian VnmrS 500MHz spectrometer and a Bruker Avance Neo 600MHz spectrometer, operating at 499.72 MHz for $^{1}H$ and 125.67 MHz for $^{13}C$ and 599.79 MHz for $^{1}H$ and 150.83 MHz for $^{13}C$ respectively. Variable temperature experiments were carried out on an Agilent DD2 500 spectrometer, operating at 500.03 MHz for $^{1}H$. Chemical shifts are with reference to the residual solvent peak, with J values in Hz. For multiplicity of the peaks, the abbreviations used are (s) singlet, (d) doublet, (t) triplet, (q) quartet and (m) multiplet. DOSY NMR was analyzed using Bruker Dynamic Center Software.

# 2. Synthetic Methods

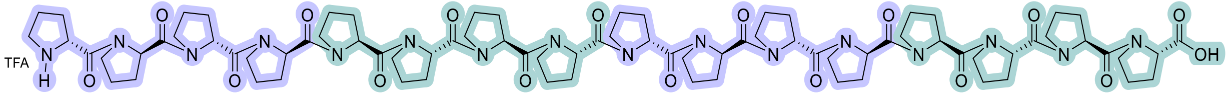

**1**

**Figure S1** Linear peptidic sequence **1** -N to -C. The sequence is made of alternating tetramers of L-Pro and D-Pro. Colour code is green for L-Proline and purple for D-Proline.

**Peptide Synthesizer Method:** The peptide **1** was synthesized on a 0.1 mmol scale on H-Proline-2-Chloro Trityl resin, which bears one Pro residue linked to the polymeric resin. The primary sequence was synthesized on a Liberty Blue CEM microwave peptide synthesizer utilizing Fmoc solid-phase peptide synthesis techniques and repeated steps of single deprotections, and single couplings interspaced with washings (4 x 4 mL DMF). From the tenth amino acid on the coupling cycle was set to double, as it is known that increasing the length of a peptide makes the coupling reaction more challenging. Deprotection: 10 % piperidine in DMF (4.5 mL) for 6 min at 70 W microwave irradiation at 40ºC. Coupling: Fmoc-amino acid (1.5 mL, 0.2 M, 3 eq.), DIC (1.2 mL, 0.5 M, 6 eq.), Oxyma Pure (0.6 mL, 0.5 M, 3 eq.) in DMF, and DMF (3 mL) for 11 min at 40ºC and 45 W microwave irradiation.

**Peptide Cleavage:** The resin was then washed with DCM (5 x 5 mL), before the Fmoc-protected peptide was cleaved on the bench from the resin with TFA (50 % in DCM) for 15 min. The resin was then washed with the cleavage cocktail (2 x 0.5mL), and the filtrate was concentrated by evaporation before precipitation in cold $Et_2O$ and centrifugation. The solution was then decanted and the solid repeatedly washed with cold $Et_2O$ to isolate the peptide, as a white solid in a quantitative yield (98%) after drying under vacuum.

**1** was obtained as a white solid; yield 98%. In $D_2O$ exists as a mixture of isomers. $^1$H NMR (600 MHz, $D_2O$) δ 5.18 – 5.15 (m, 1H), 4.75 – 4.68 (m, 7H), 4.67 – 4.63 (m, 1H), 4.46 – 4.42 (m, 1H), 4.34 – 4.28 (m, 1H), 3.90 – 3.36 (m, 32H), 2.62 – 2.54 (m, 1H), 2.50 – 2.25 (m, 14H), 2.19 – 1.75 (m, 47H), 1.71 – 1.78 (m,1H), 1.66 – 1.64 (m, 1H). $^{13}$C NMR (151 MHz, $D_2O$, peaks split due to isomers) δ 175.95, 172.12, 172.09, 172.04, 171.62, 171.60, 171.57, 171.49, 171.47, 171.40, 171.30, 171.08, 167.60, 163.10, 162.87, 127.24, 117.28, 115.35, 59.34, 59.18, 59.07, 59.00, 58.85, 58.69, 58.60, 48.76, 47.68, 47.44, 47.32, 46.61, 30.24, 28.72, 28.20, 28.14, 27.98, 27.88, 27.81, 25.82, 25.04, 24.92, 24.65, 24.54, 24.35, 24.18, 23.81, 23.45, 22.00. HRMS: calcd for $[M+H]^+$: $C_{80}H_{114}N_{16}O_{17}$, 1571.8581; found; $[M+H]^+$ 1571.8609.

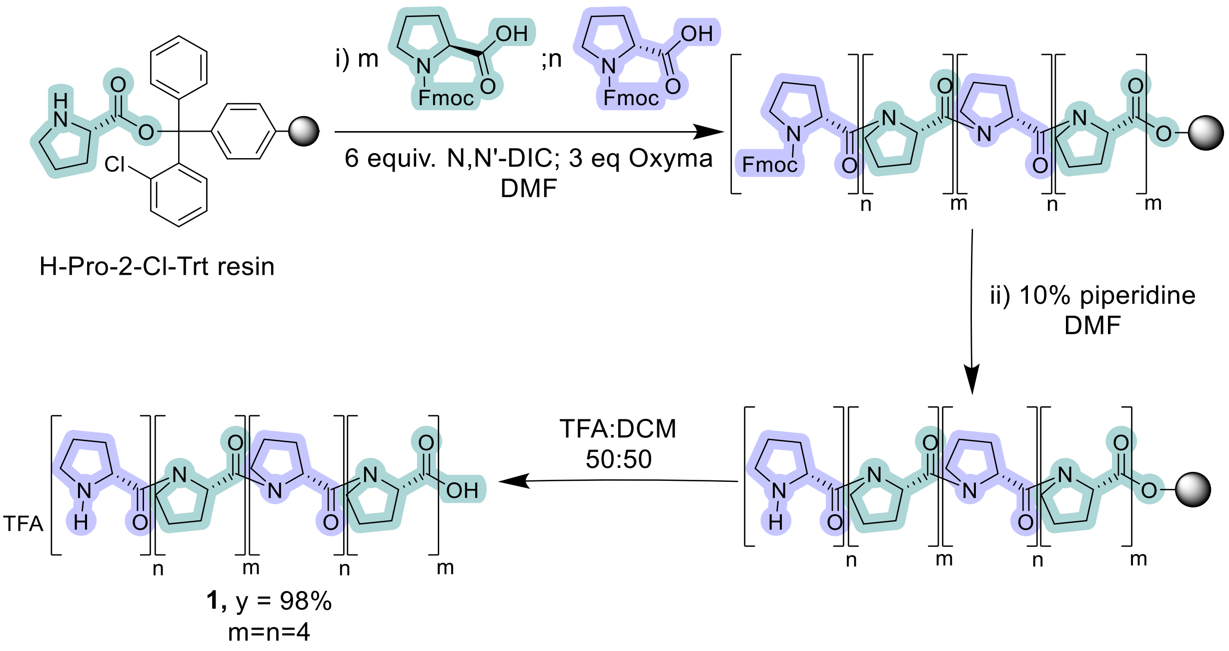


**Scheme S1** Reaction scheme of the step-by-step synthesis to obtain the linear peptide **1**: couplings and deprotections are performed on a Liberty Blue Automated Microwave Peptide Synthesizer CEM. The cleavage of the peptide from the resin is performed on the bench.

### Synthesis of the macrocyclic peptide CP[4,4]:

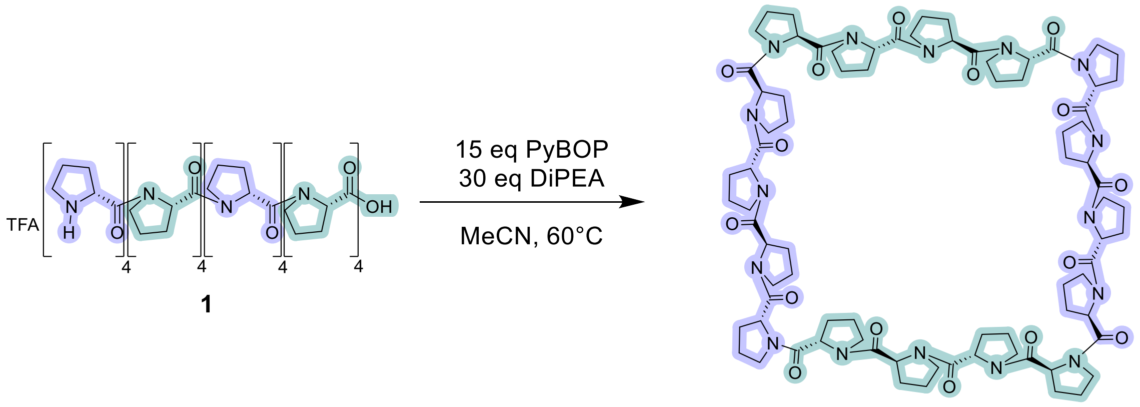


**CP[4,4]**
y = 43%

**Scheme S2** Macrocyclization reaction scheme of linear sequence **1**, yielding the macrocyclic structure **CP[4,4]**.

To a stirred solution of PyBOP (298 mg, 15 equiv) and DIPEA (148 mg, 200 µL, 30 equiv) in MeCN (60 mL) at 60 °C was added a solution of the linear peptide **1** (60 mg) in MeCN (18 mL) via syringe pump over 6 h (rate: 3 mL/h). The reaction mixture was stirred at 60 °C for an additional 30 min. Successively, the reaction mixture was concentrated under reduced pressure and the product precipitated in cold $Et_2O$ and centrifugated, this procedure was repeated twice. The washing solution was then decanted and the solid dissolved in water (approximately 1 mL/100 mg of crude product), then sonicated for half an hour and centrifuged again. This procedure was assessed to be key in reducing PyBOP byproducts as well as either oligomers or polymers that form during the reaction. The formation of the macrocyclic peptide **CP[4,4]** was confirmed by LC-HRMS analysis and $^{1}$H-NMR and purified by semipreparative HPLC. The overall yield was 43%.

**CP[4,4]** was obtained as a white solid; yield 43%. In $D_2O$, **CP[4,4]**, exists as a mixture of isomers. $^1$H NMR (600 MHz, $D_2O$) δ 5.27 (dd, *J* = 9.1, 3.6 Hz, 1H, all-junctions-*cis* **CP[4,4]** isomer), 4.91 (dd, *J* = 8.7, 1.6 Hz, 1H, all-junctions-*cis* **CP[4,4]** isomer), 4.88 (dd, *J* = 8.6, 2.4 Hz, 1H, all-junctions-*trans* **CP[4,4]** isomer), 4.76 – 4.73 (m, 2H,mix of all-junctions-*cis* and all-junctions-*trans* **CP[4,4]** isomers), 4.71 – 4.68 (m, 1H, all-junctions-*cis* **CP[4,4]** isomer), 4.66 – 4.61 (m, 3H, mix of all-junctions-*cis* and all-junctions-*trans* **CP[4,4]** isomer), 4.06 (t, *J* = 6.8 Hz, 1H, all-junctions-*trans* **CP[4,4]** isomer), 3.99 – 3.95 (m, 1H, all-junctions-*trans* **CP[4,4]** isomer), 3.88 – 3.52 (m, 22H), 2.50 – 2.43 (m, 1H), 2.39 – 1.77 (m, 47H). $^{13}$C NMR (151 MHz, $D_2O$, peaks split due to isomers) δ 173.55, 172.60, 172.54, 172.30, 171.91, 171.44, 171.10, 170.91, 59.19, 58.80, 58.78, 58.64, 58.41, 58.15, 48.04, 47.82, 47.76, 47.43, 47.35, 47.33, 47.23, 47.20, 30.42, 28.59, 28.42, 28.10, 27.87, 27.75, 27.68, 27.59, 25.13, 24.94, 24.89, 24.87, 24.62, 23.77, 23.69, 22.48. HRMS: calcd for $[M+H]^+$: $C_{80}H_{112}N_{16}O_{16}$, 1553.8515; found; $[M+H]^+$ 1553.8510.

**CP[4,4]**, in $CDCl_3$, exists as a single isomer, the all-junctions-*cis* **CP[4,4]**. $^1$H NMR (600 MHz, $CDCl_3$) δ 5.36 (dd, *J* = 9.3, 3.3 Hz, 4H), 4.79 (d, *J* = 7.8 Hz, 4H), 4.69 (dd, *J* = 8.5, 4.4 Hz, 4H), 4.10 (dd, *J* = 8.5, 2.7 Hz, 4H), 3.78 – 3.75 (m, 4H), 3.71 – 3.56 (m, 30H), 2.47 – 2.39 (m, 4H), 2.36 – 2.29 (m, 4H), 2.24 – 1.81 (m, 69H). $^{13}$C NMR (151 MHz, $CDCl_3$) δ 171.93, 171.62, 170.28, 58.27, 58.19, 58.08, 57.98, 47.38, 47.19, 47.05, 46.62, 31.07, 29.85, 29.21, 27.98, 27.95, 25.33, 25.00, 24.03, 22.97, 14.27.

### Optimization of the synthesis of the macrocyclic peptide CP[4,4]:

The reaction conditions mentioned above are the result of an optimization process. The screening is reported in the following table. The entry 7 reports the ideal conditions (Scheme S2). The other attempts all lead to formation of oligomeric product.

**Table S1** Macrocyclization reaction conditions screening.

| **Entry** | **No. of equivalents (coupling reagent, base)** | **Temperature (°C)** | **Addition flow rate (mL/hr)** | **Total reaction time (hr)** | **$V_{RBF}$ (m/[mM])** | **Outcomes** |
|---|---|---|---|---|---|---|
| **1** | 6, 12 | r.t. | No slow addition | 72 hr | 250/[0.012] | Mix of desired and oligomeric product |
| **2** | 15,30 | 60 | 1 mL/2 hr | 2 hr 15 min | 5/[0.53] | Major product is oligomers |
| **3** | 15,30 | 60 | 1 mL/4 hr | 6 hr 30 min | 5/[0.53] | Little product formed |
| **4** | 15,30 | r.t. | 4 mL/8 hr | 20 hr | 60/[0.052] | Mix of desired and oligomeric product |
| **5** | 15,30 | 60 | 4 mL/10 hr | 20 hr | 20/[0.53] | Product not formed |
| **6** | 15,30 | 60 | 10 mL/12 hr | 20 hr | 50/[0.53] | Desired product is the major but too long reaction time |
| **7** | 15,30 | 60 | 18 mL/6 hr | 6 hr 30 min | 60/[0.49] | Ideal conditions |

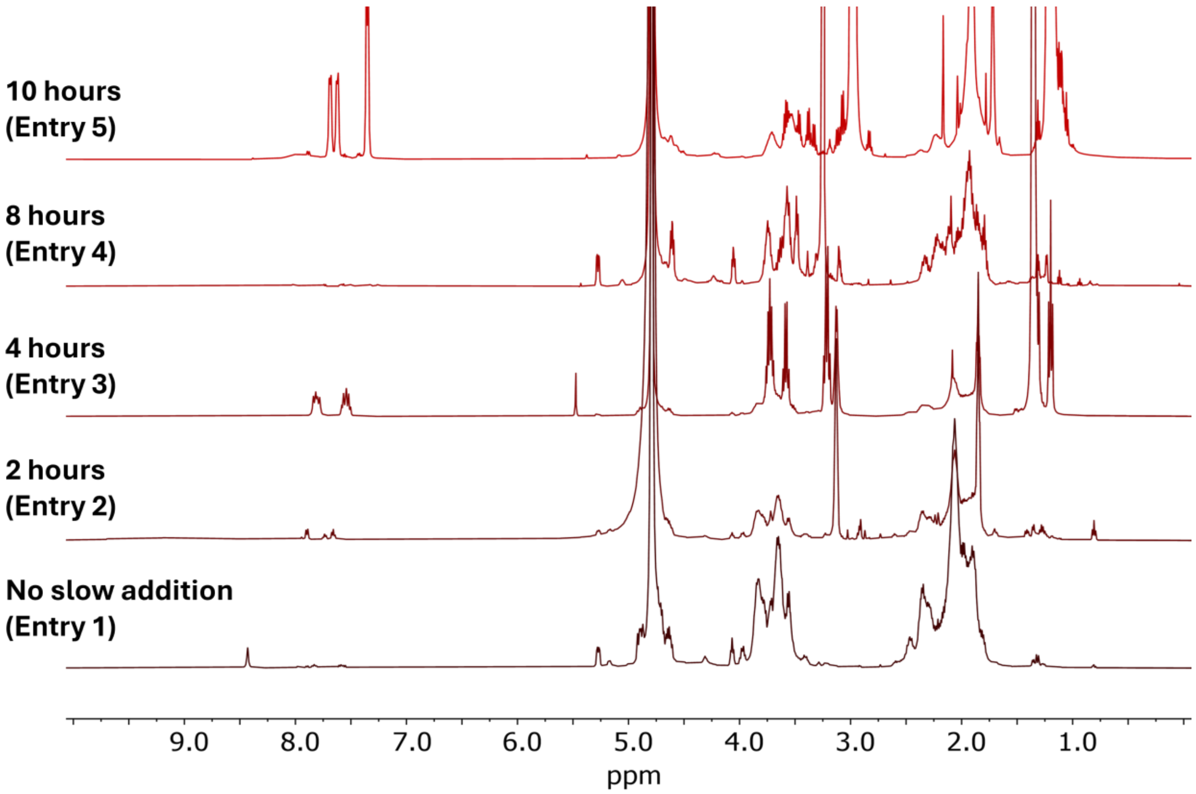


**Figure S2** Stacked $^{1}$H-NMRs of macrocyclization reactions that yielded a majority of oligomeric product.

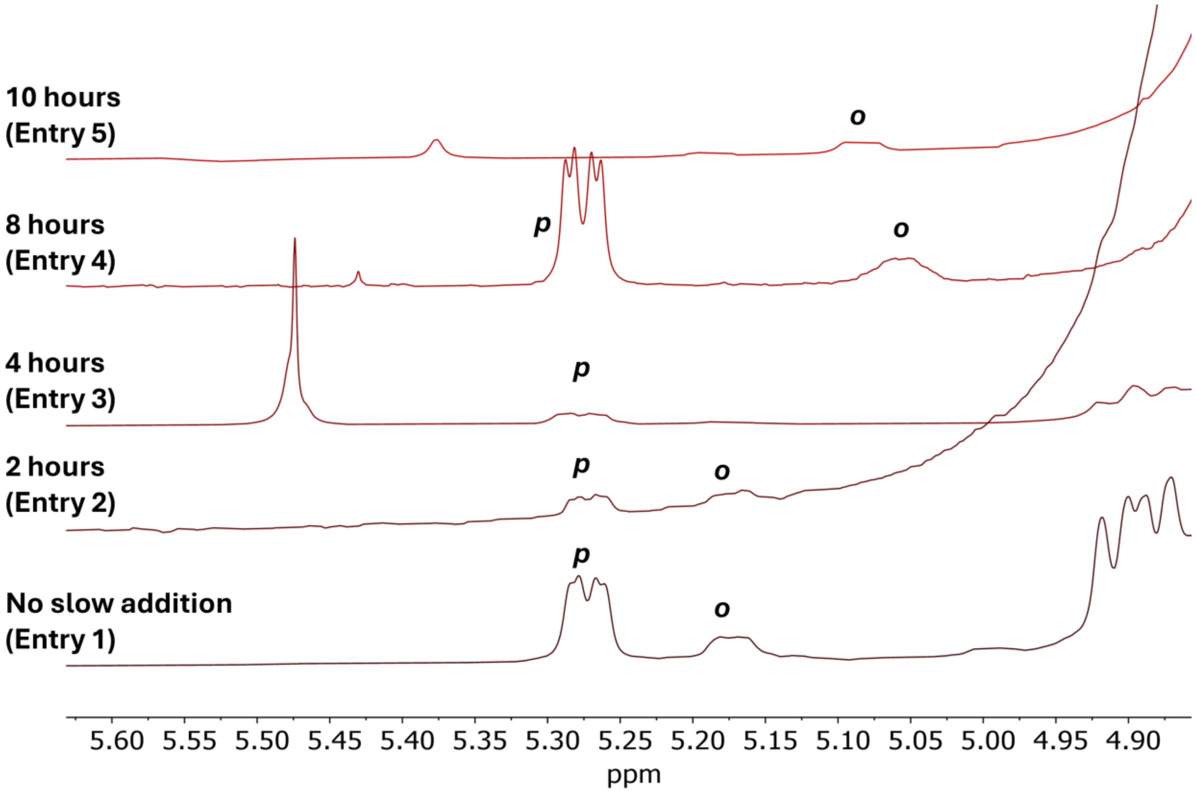


**Figure S3** Expansion of stacked $^{1}$H-NMRs of macrocyclization reactions that yielded a majority of oligomeric product. Letter ***o*** stands for oligomeric product and letter ***p*** for desired product.

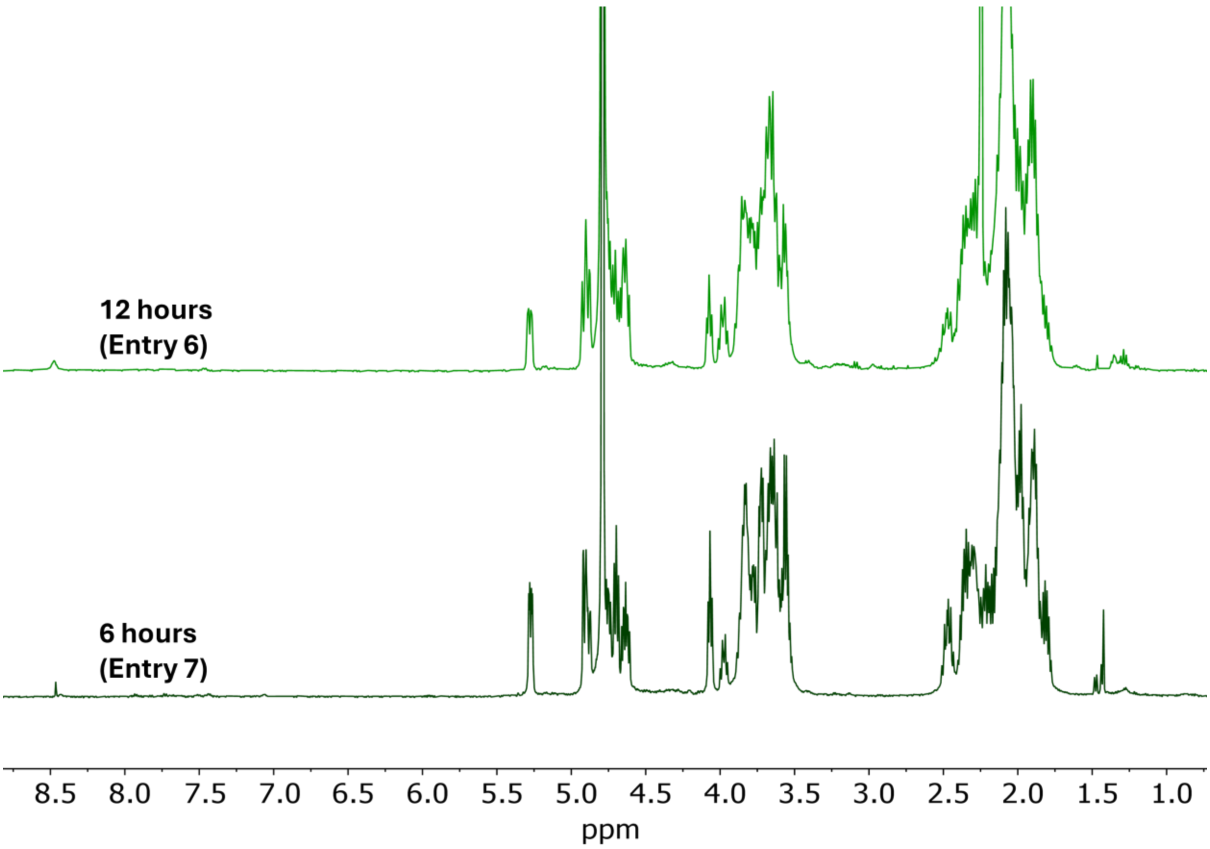


**Figure S4** Stacked $^{1}$H-NMRs of macrocyclization reactions that yielded the desired product.

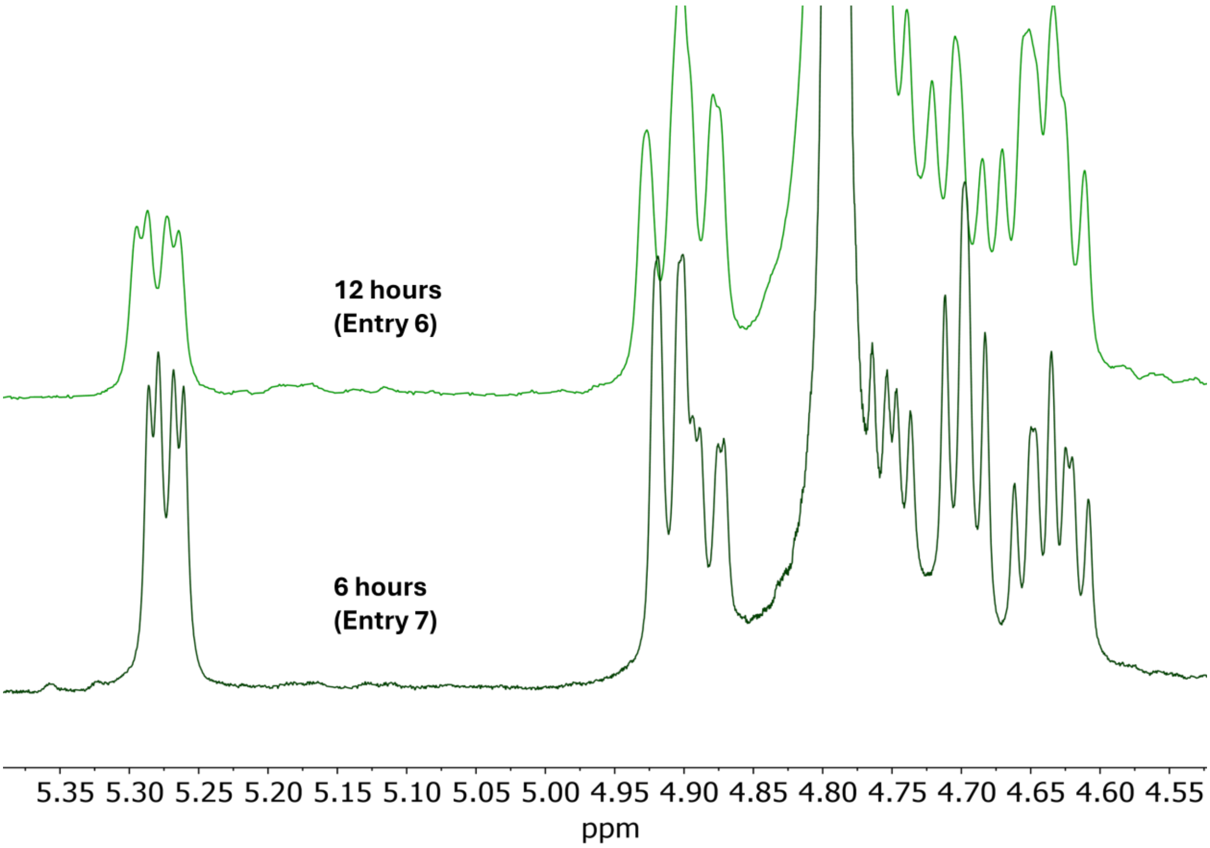


**Figure S5** Expansion of stacked $^{1}$H-NMRs of macrocyclization reactions that yielded the desired product.

**Synthesis of the Benzidine bis(trifluoroacetate) 2*TFA*:** Benzidine (100mg, 0.543 mmol) was dissolved in $CHCl_3$ (10 mL) and cooled to 0ºC, Trifluoroacetic acid (TFA, 2.0 equiv, 1.086 mmol, 830 µL) was added dropwise to afford a final solution concentration of approximately 1 M. The reaction mixture was then allowed to warm to room temperature and stirred for 1 h, resulting in formation of the corresponding benzidine bis(trifluoroacetate) salt. The solution was dried and **2*TFA*** was weighed as needed.

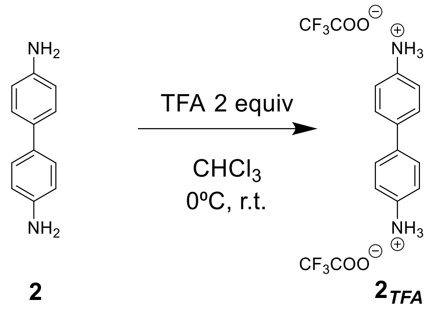


**Scheme S3** Formation of Benzidine bis(trifluoroacetate) **2*TFA***

**Synthesis of the Benzidine Hydrochloride 2*Cl*:** Benzidine (100 mg, 0.543 mmol) was dissolved in MeOH (5 mL). A 3 M HCl solution in MeOH was prepared by addition of acetyl chloride (2.7 mL, 37.9 mmol) to MeOH (10 mL). An aliquot of this solution (5 mL, 15.0 mmol HCl, 27.6 equiv) was added to the benzidine solution. The resulting mixture was stirred to afford the corresponding benzidine hydrochloride salt. The solution was dried and **2*Cl*** was weighed as needed.

# 3. NMR Spectroscopy Data

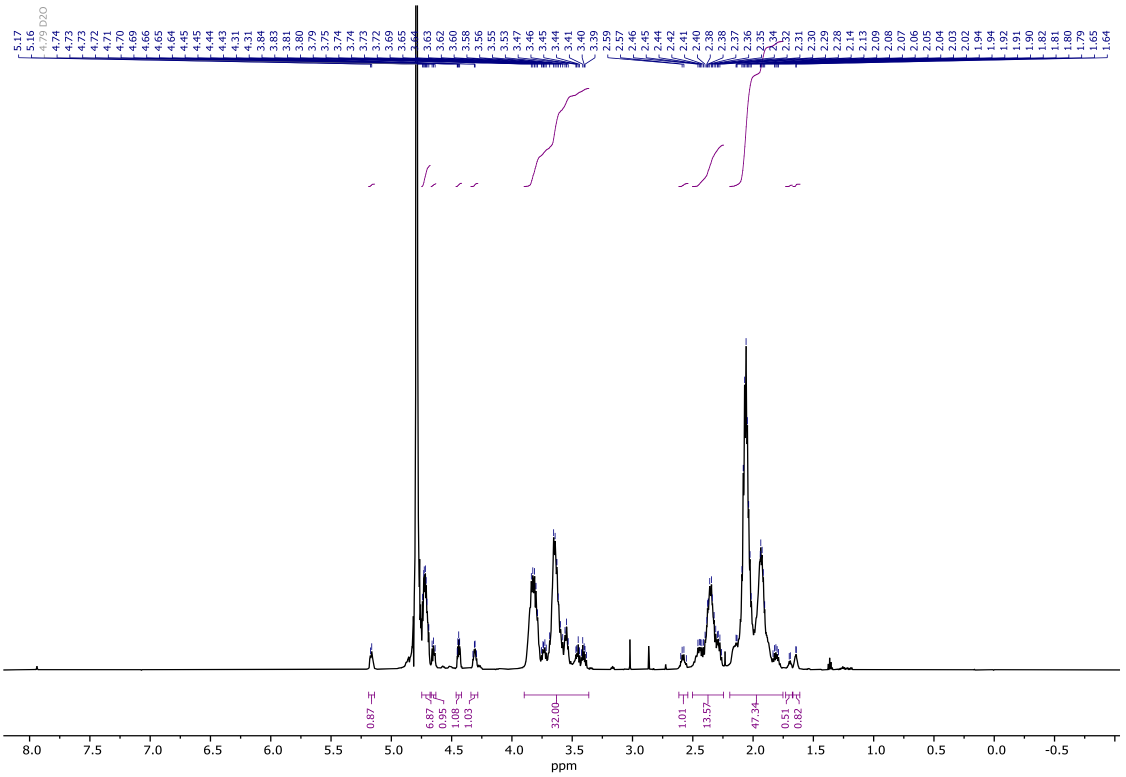

**Figure S6** $^{1}$H-NMR spectrum of **1** (600 MHz, $D_2O$, 298 K).

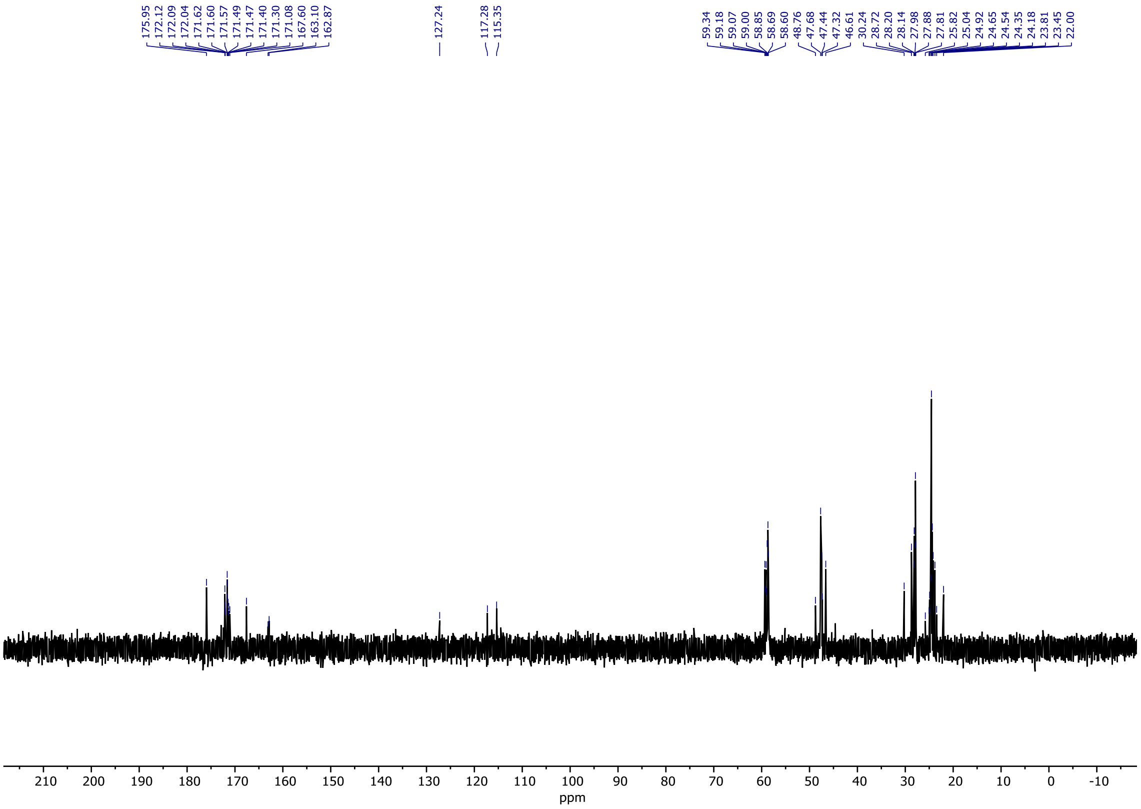

**Figure S7** $^{13}$C-NMR spectrum of **1** (151 MHz, $D_2O$, 298 K).

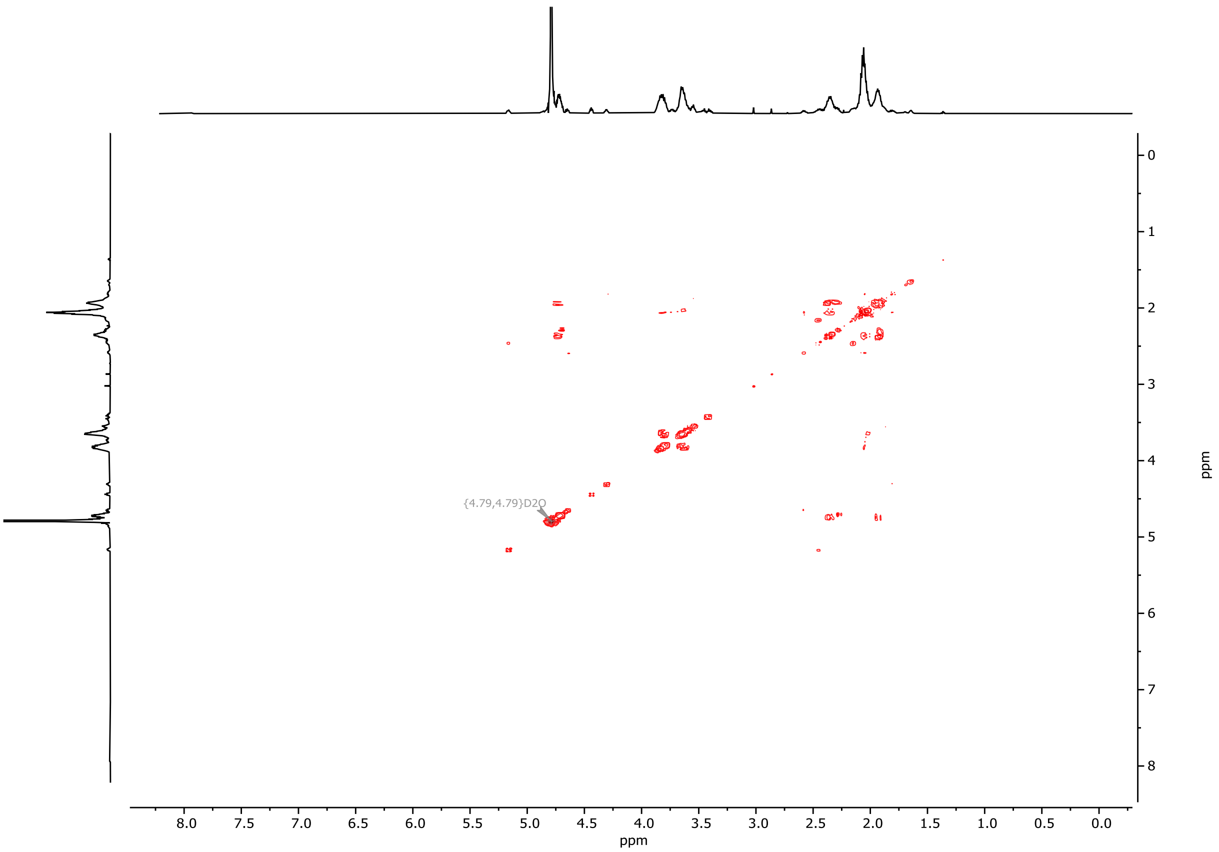


**Figure S8** $^1$H-$^1$H COSY NMR spectrum of **1** (600 MHz, $D_2O$, 298 K).

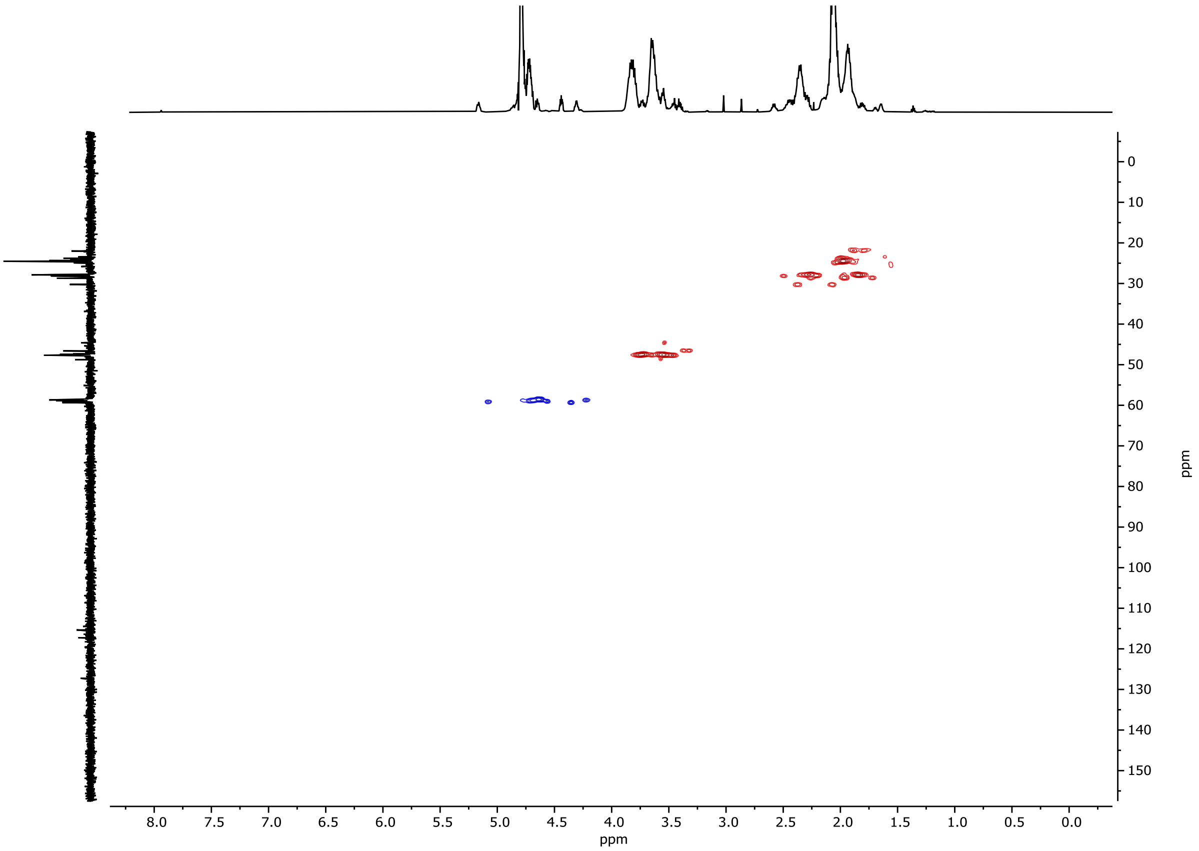


**Figure S9** $^1$H-$^{13}$C HSQC NMR spectrum of **1** (600-151 MHz, $D_2O$, 298 K).

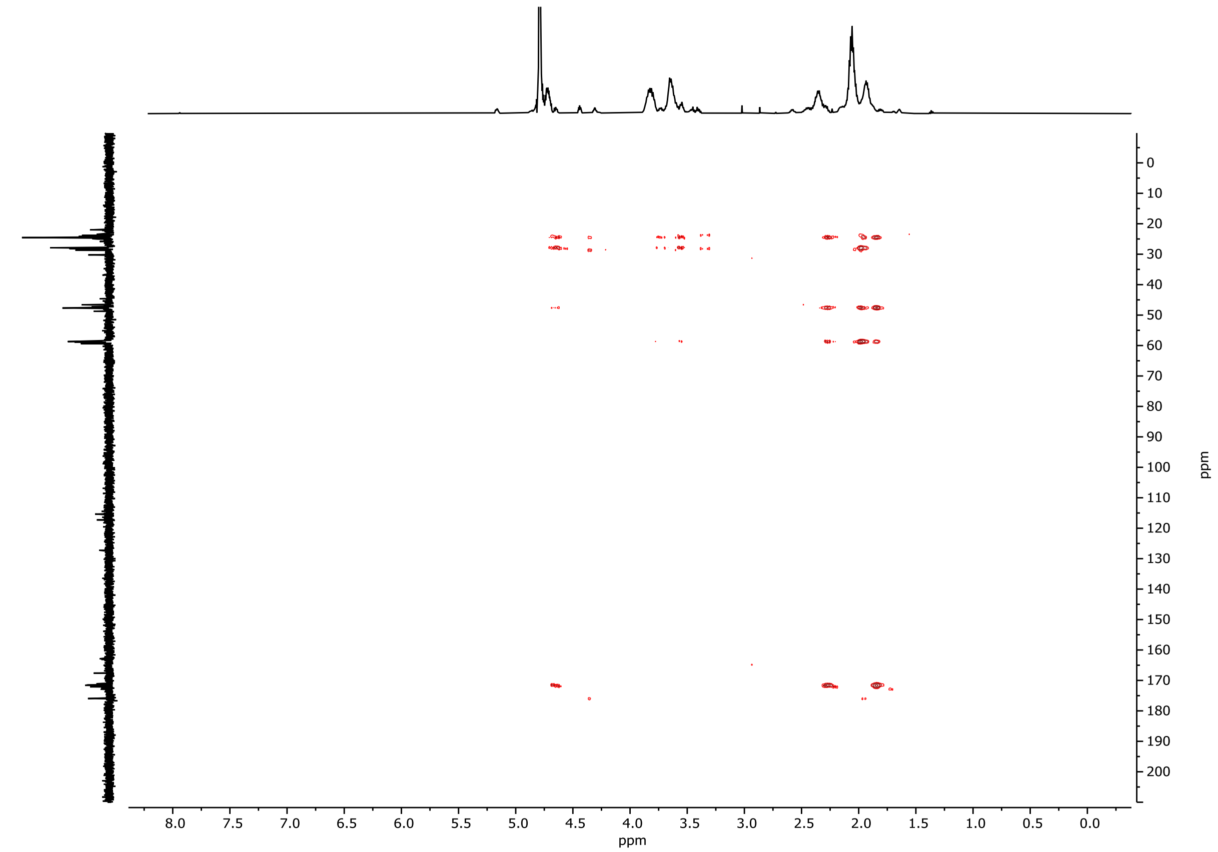


**Figure S10** $^{1}H$-$^{13}C$ HMBC NMR spectrum of **1** (600-151 MHz, $D_2O$, 298 K).

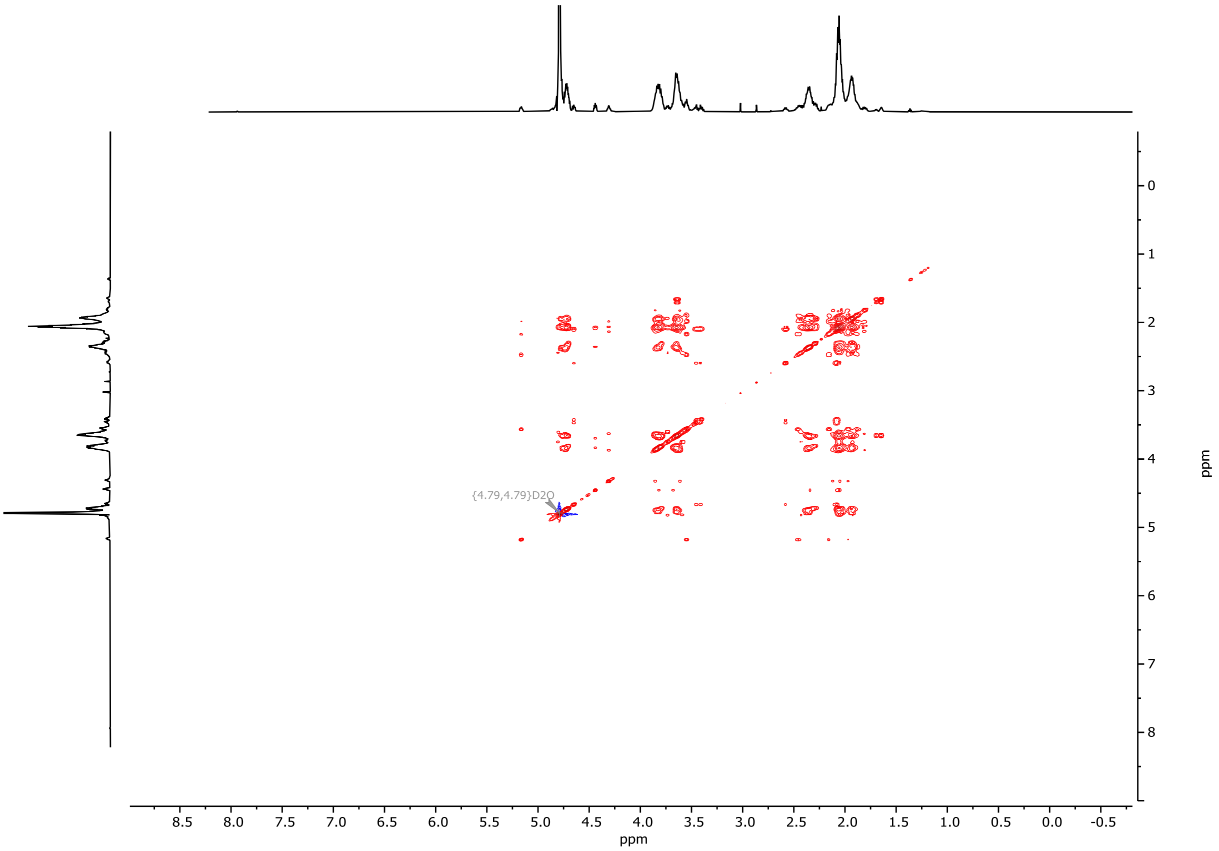


**Figure S11** $^{1}H$-$^{1}H$ TOCSY NMR spectrum of **1** (600 MHz, $D_2O$, 298 K).

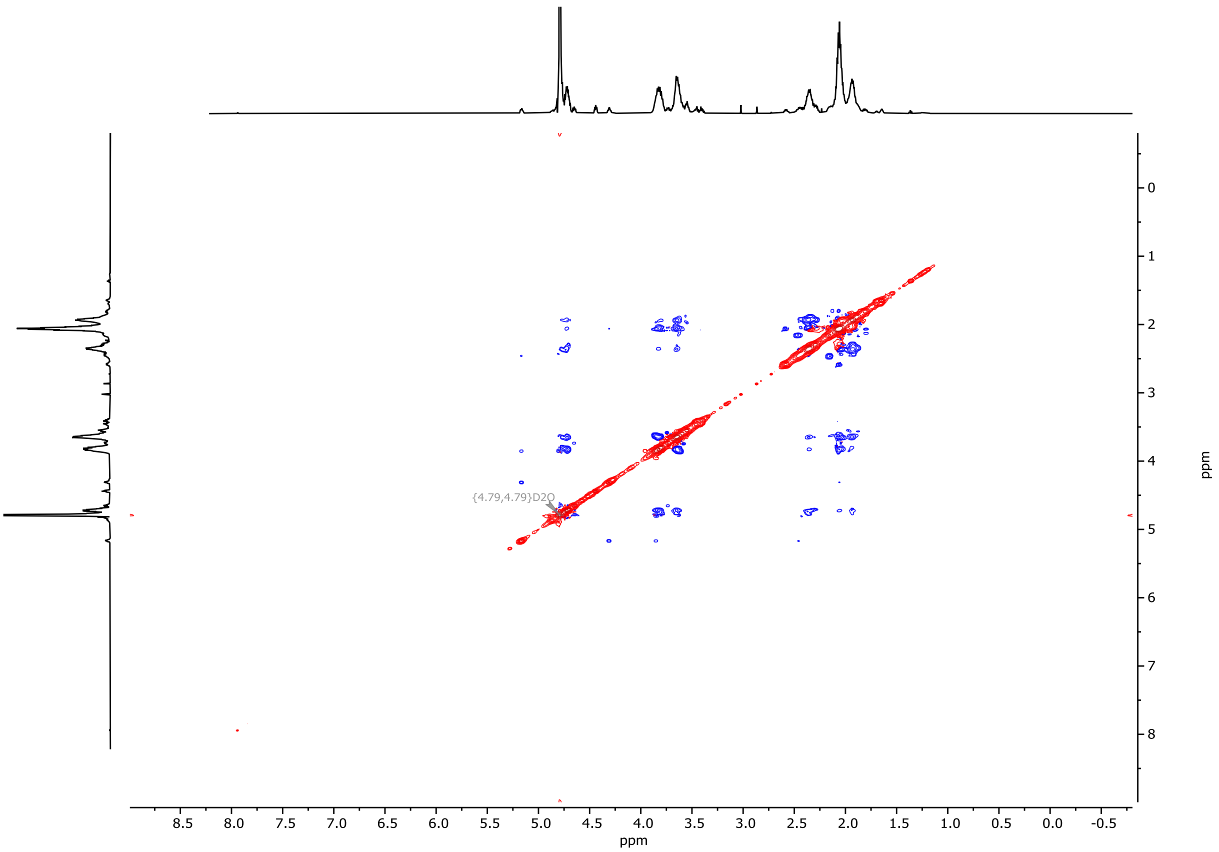


**Figure S12** $^1H$-$^1H$ ROESY NMR spectrum of **1** (600 MHz, $D_2O$, 298 K).

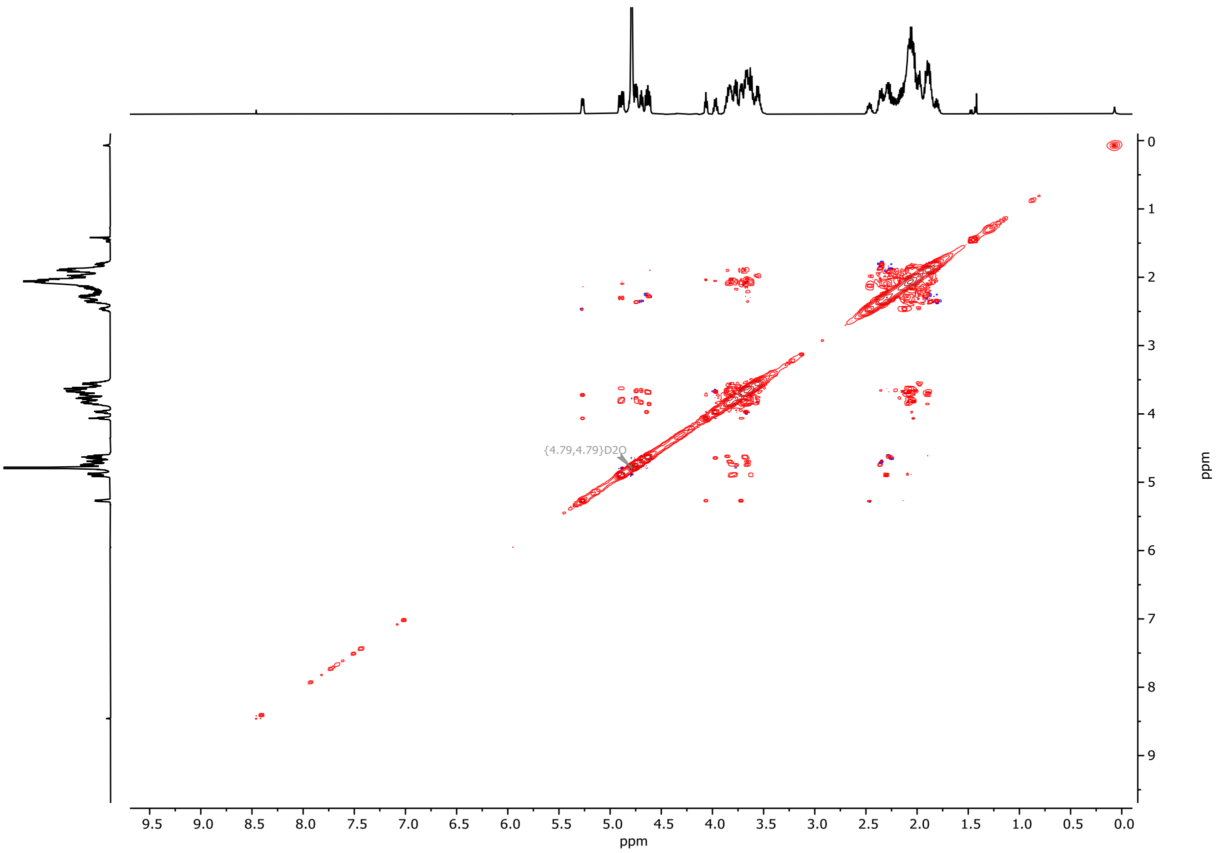


**Figure S13** $^1H$-$^1H$ NOESY NMR spectrum of **1** (600 MHz, $D_2O$, 298 K).

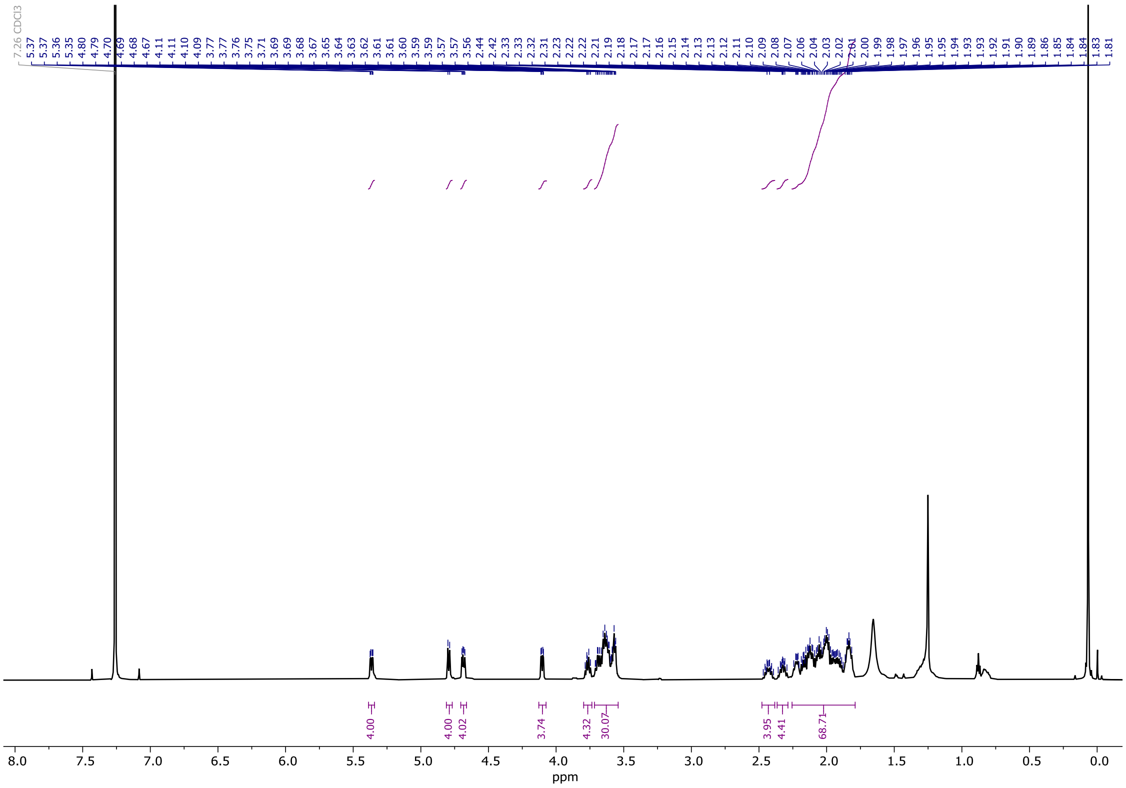

**Figure S14** $^{1}$H-NMR spectrum of **CP[4,4]** (600 MHz, $CDCl_3$, 298 K).

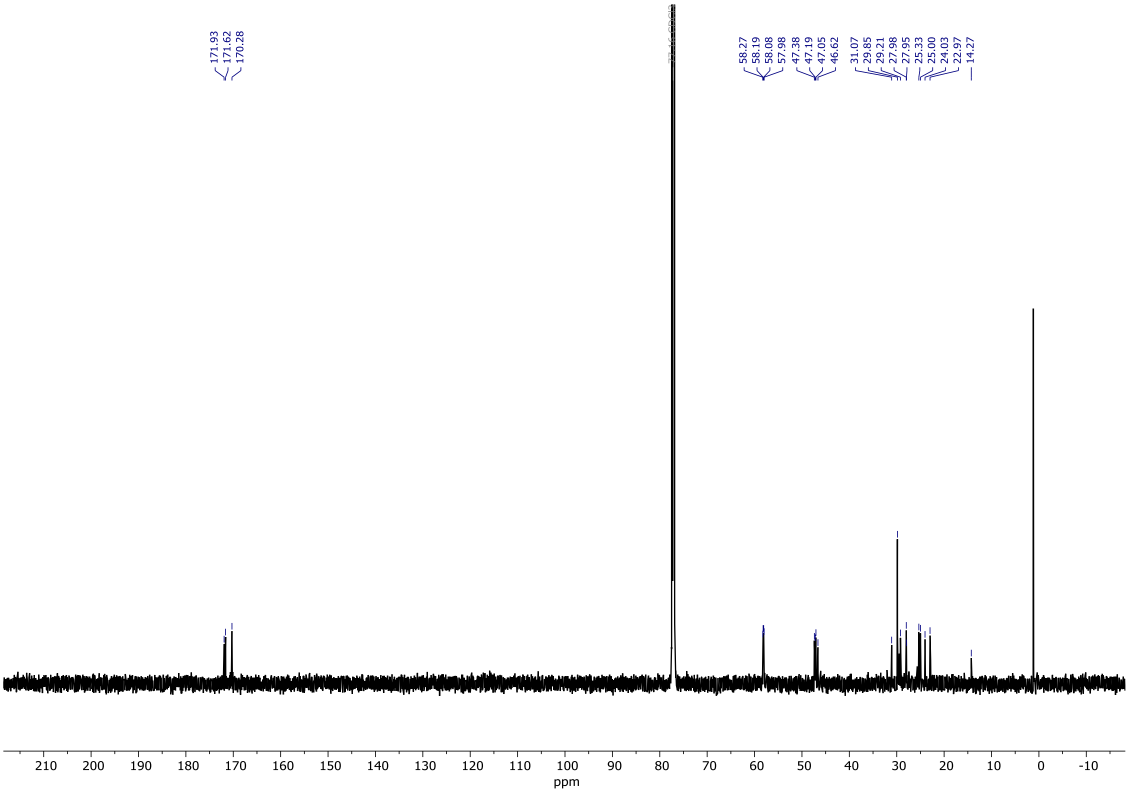

**Figure S15** $^{13}$C-NMR spectrum of **CP[4,4]** (151 MHz, $CDCl_3$, 298 K).

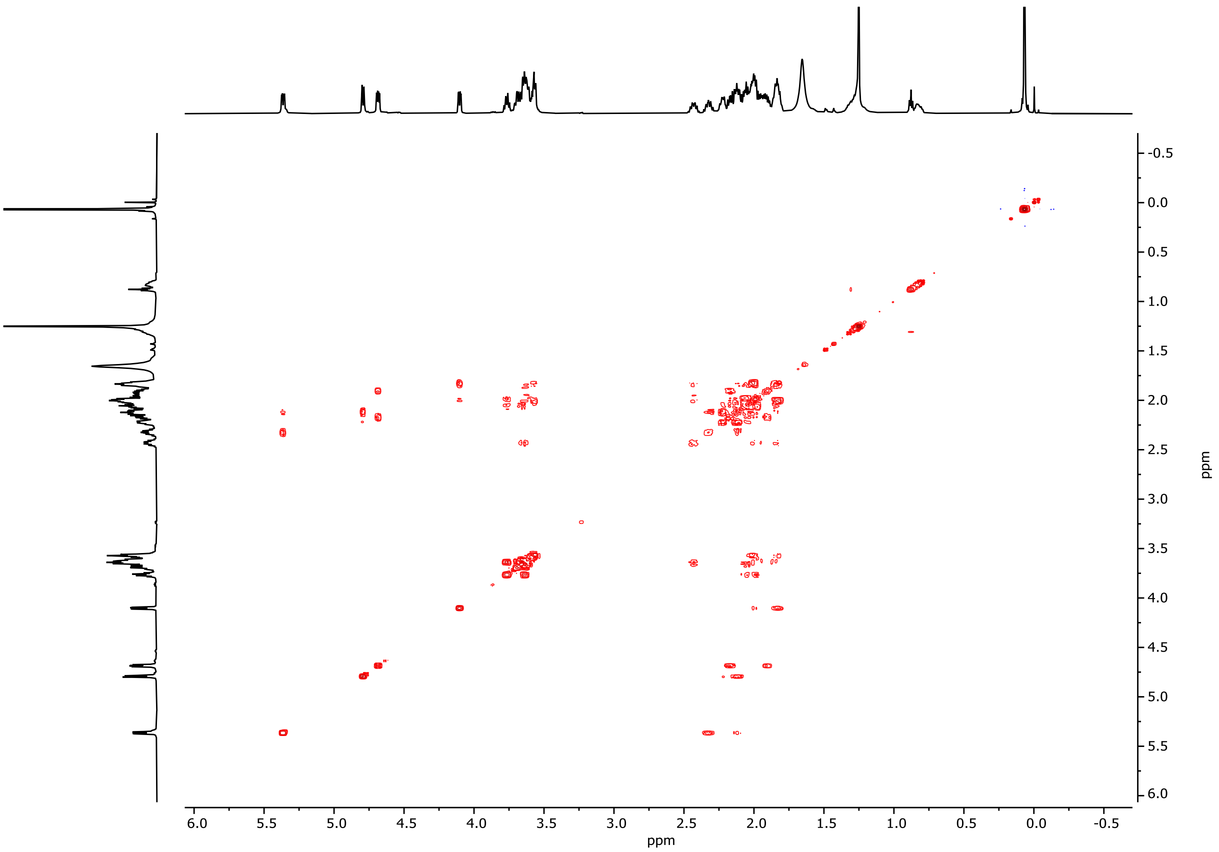


**Figure S16** $^1$H-$^1$H COSY NMR spectrum of **CP[4,4]** (600 MHz, $CDCl_3$, 298 K).

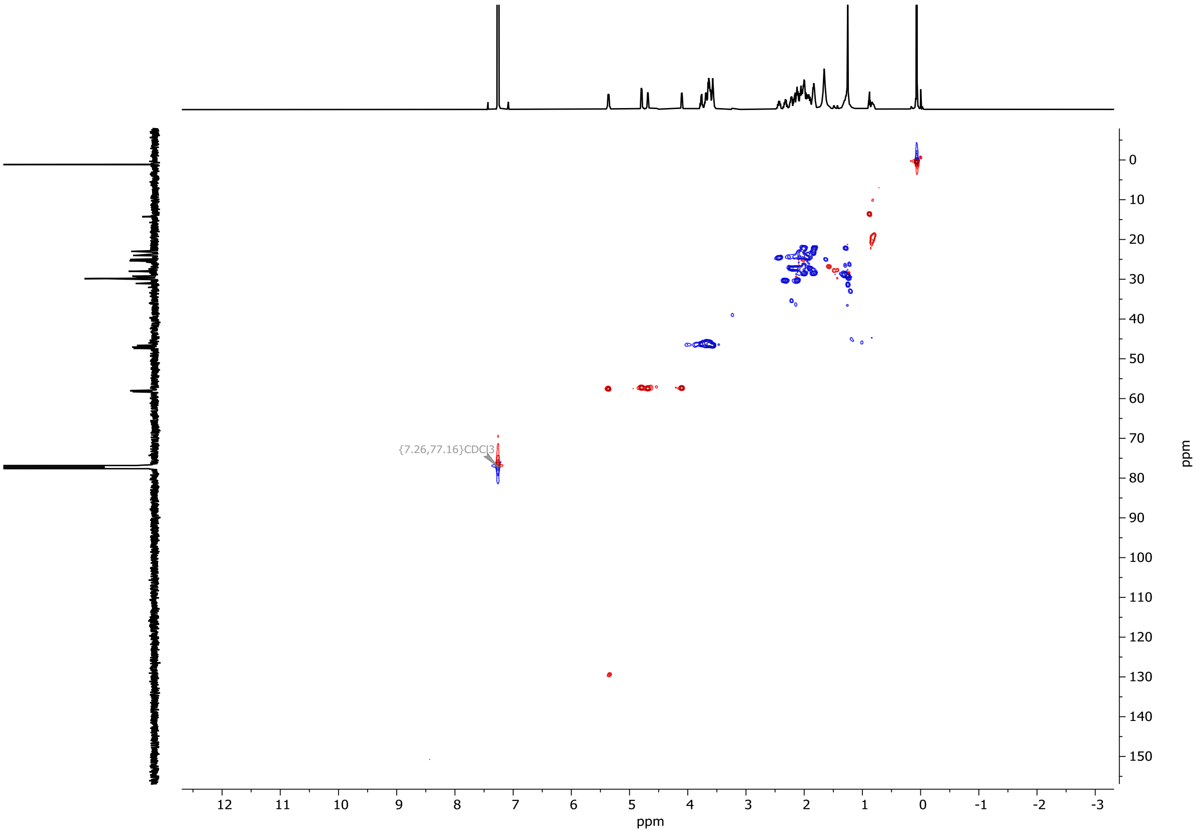


**Figure S17** $^1$H-$^{13}$C HSQC NMR spectrum of **CP[4,4]** (600-151 MHz, $CDCl_3$, 298 K).

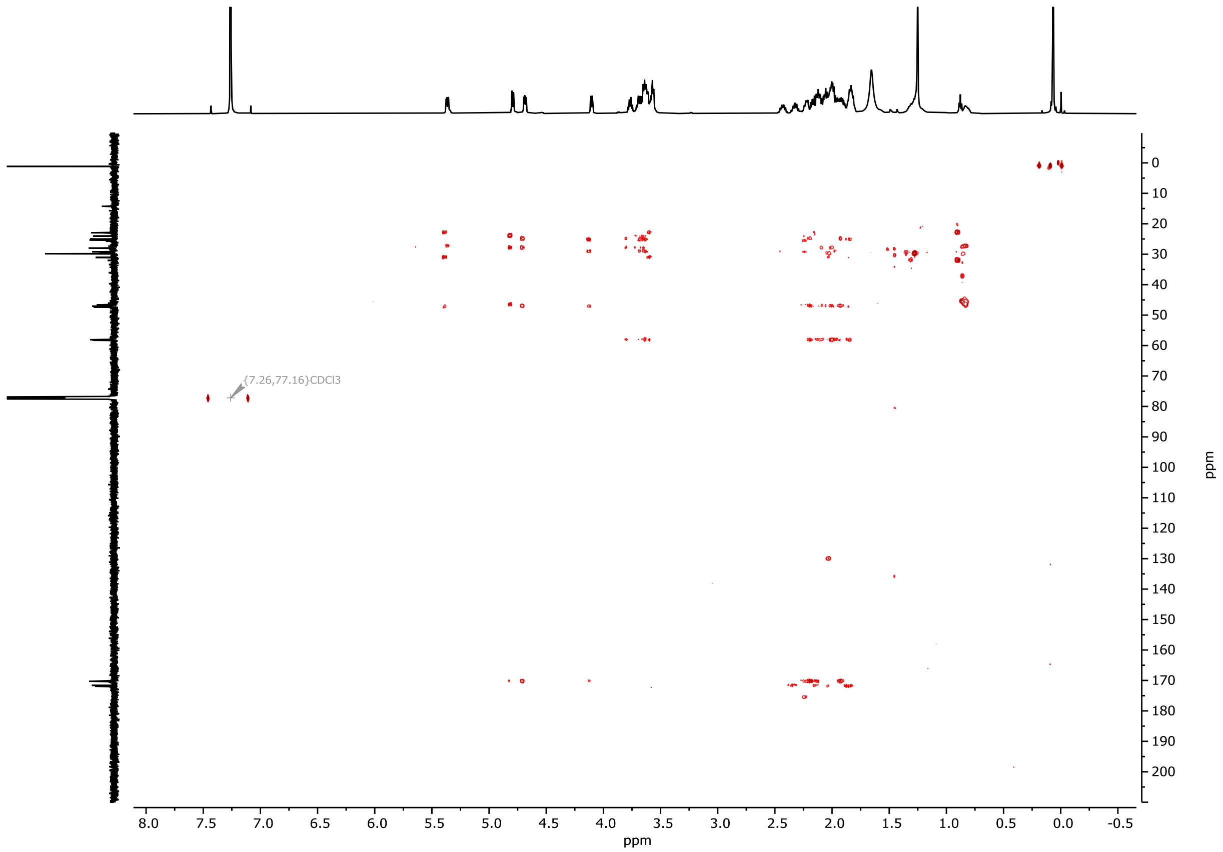


**Figure S18** $^1H$-$^{13}C$ HMBC NMR spectrum of **CP[4,4]** (600-151 MHz, $CDCl_3$, 298 K).

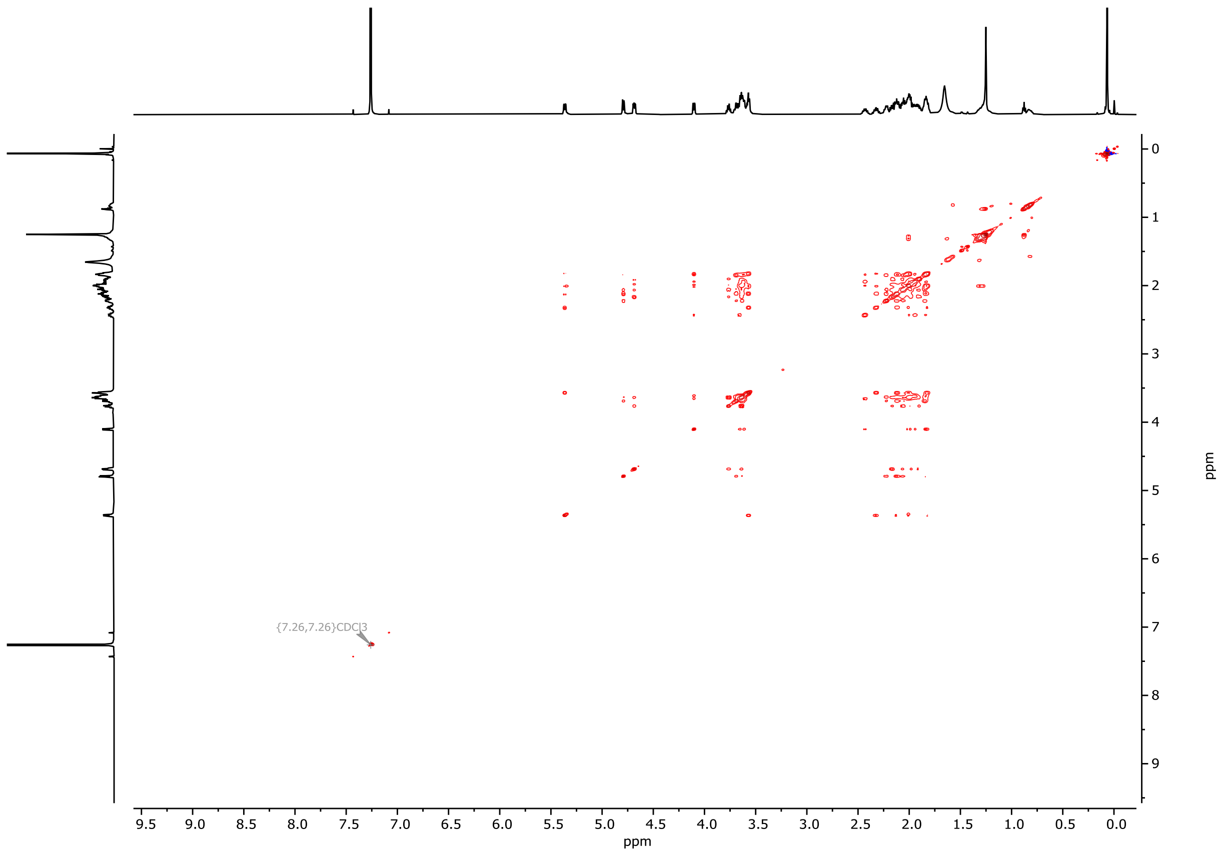


**Figure S19** $^1H$-$^1H$ TOCSY NMR spectrum of **CP[4,4]** (600 MHz, $CDCl_3$, 298 K).

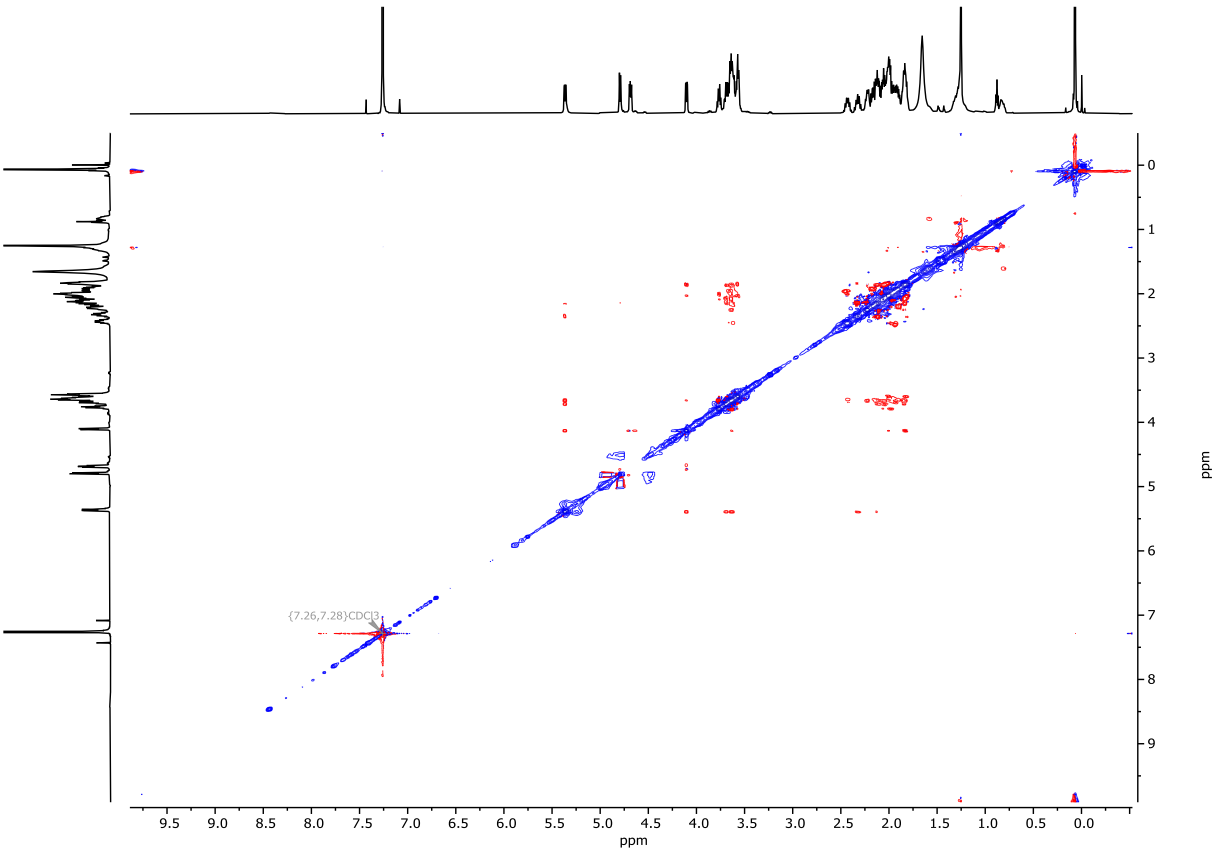


**Figure S20** $^1H$-$^1H$ ROESY NMR spectrum of **CP[4,4]** (600 MHz, $CDCl_3$, 298 K).

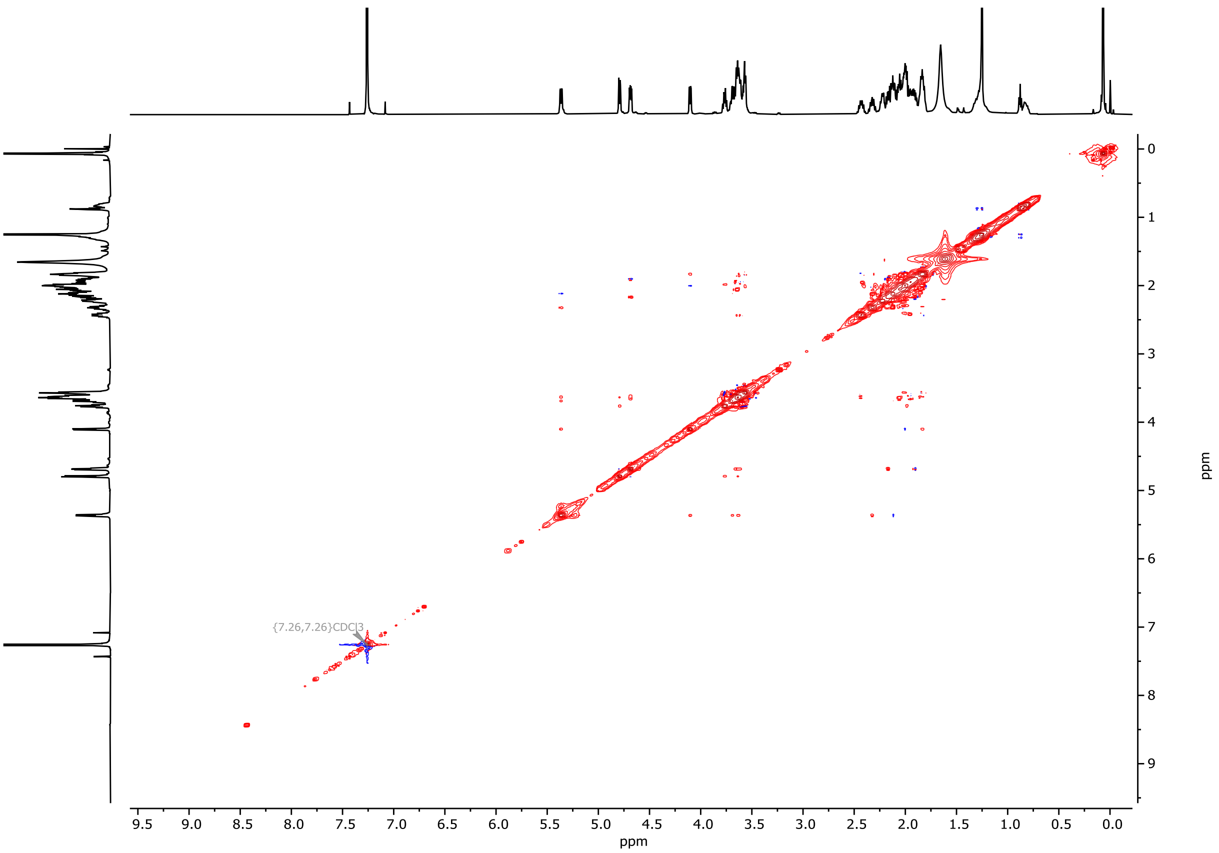


**Figure S21** $^1H$-$^1H$ NOESY NMR spectrum of **CP[4,4]** (600 MHz, $CDCl_3$, 298 K).

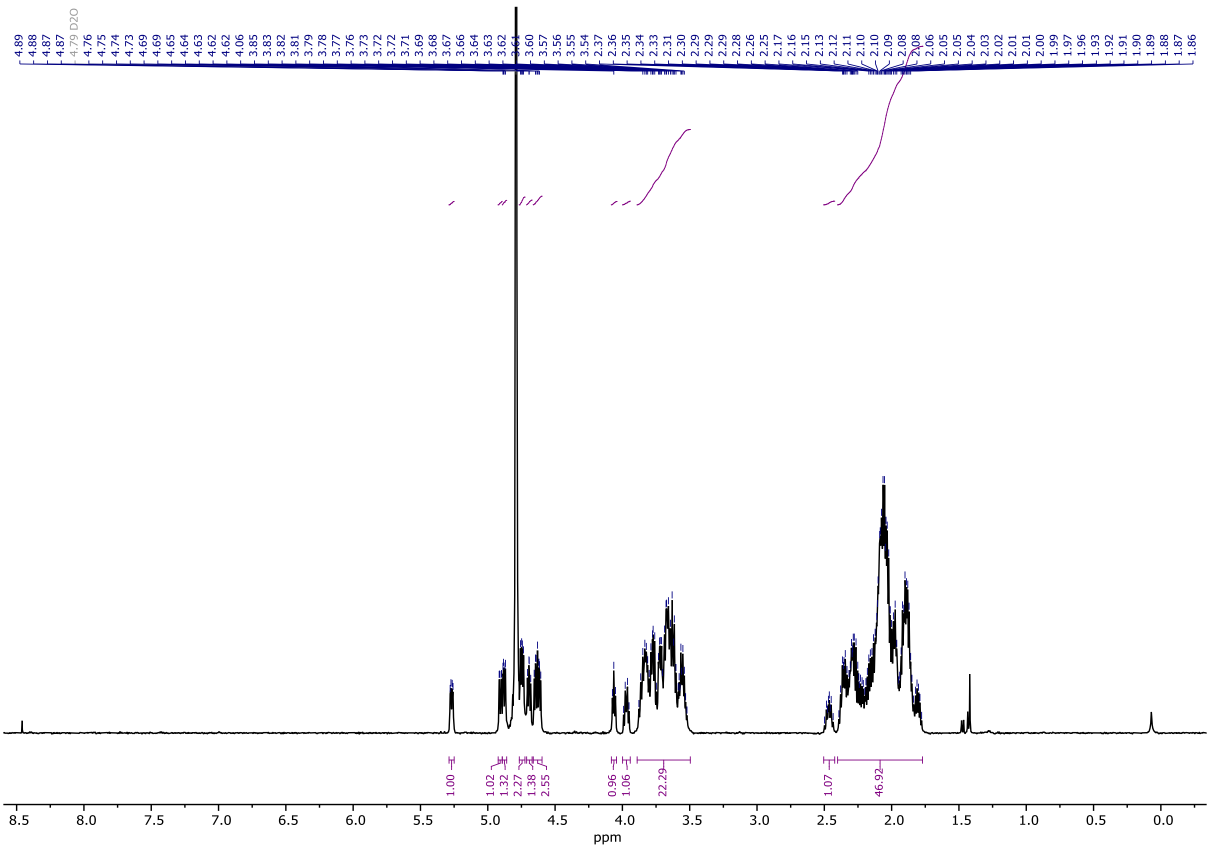


**Figure S22** $^{1}$H-NMR spectrum of **CP[4,4]** (600 MHz, D2O, 298 K).

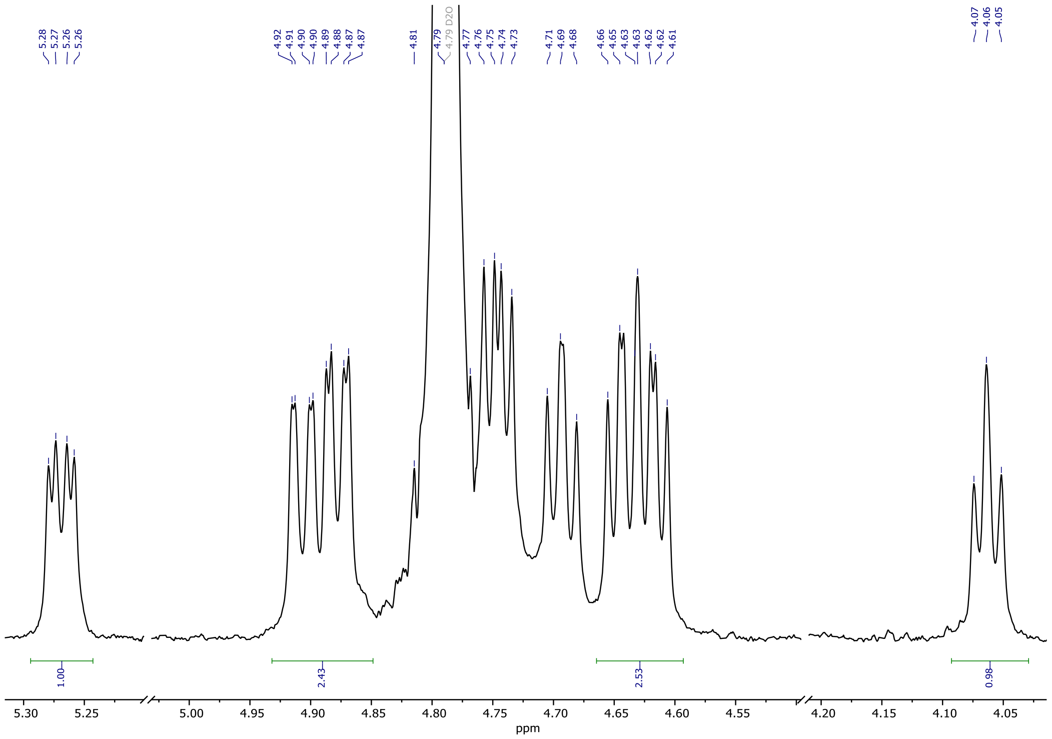


**Figure S23** Expansion of the α-proton region in the $^{1}$H NMR spectrum of **CP[4,4]** (in $D_2O$ at 298 K), used to calculate the isomers ratio. The signal at 5.27 ppm, corresponding to the all-junctions-*cis* isomer, was normalized to an integral of 1. The signals between 4.92 and 4.87 ppm consist of two doublets of doublets, one from each isomer. Similarly, the signals between 4.66 and 4.61 ppm result from the overlap of peaks from both isomers. Integration of these signals from the quantitative NMR spectrum indicates an isomer ratio of approximately 40% all-junctions-*cis* **CP[4,4]** to 60% all-junctions-*trans* **CP[4,4]**. (600 MHz, D2O, 298 K).

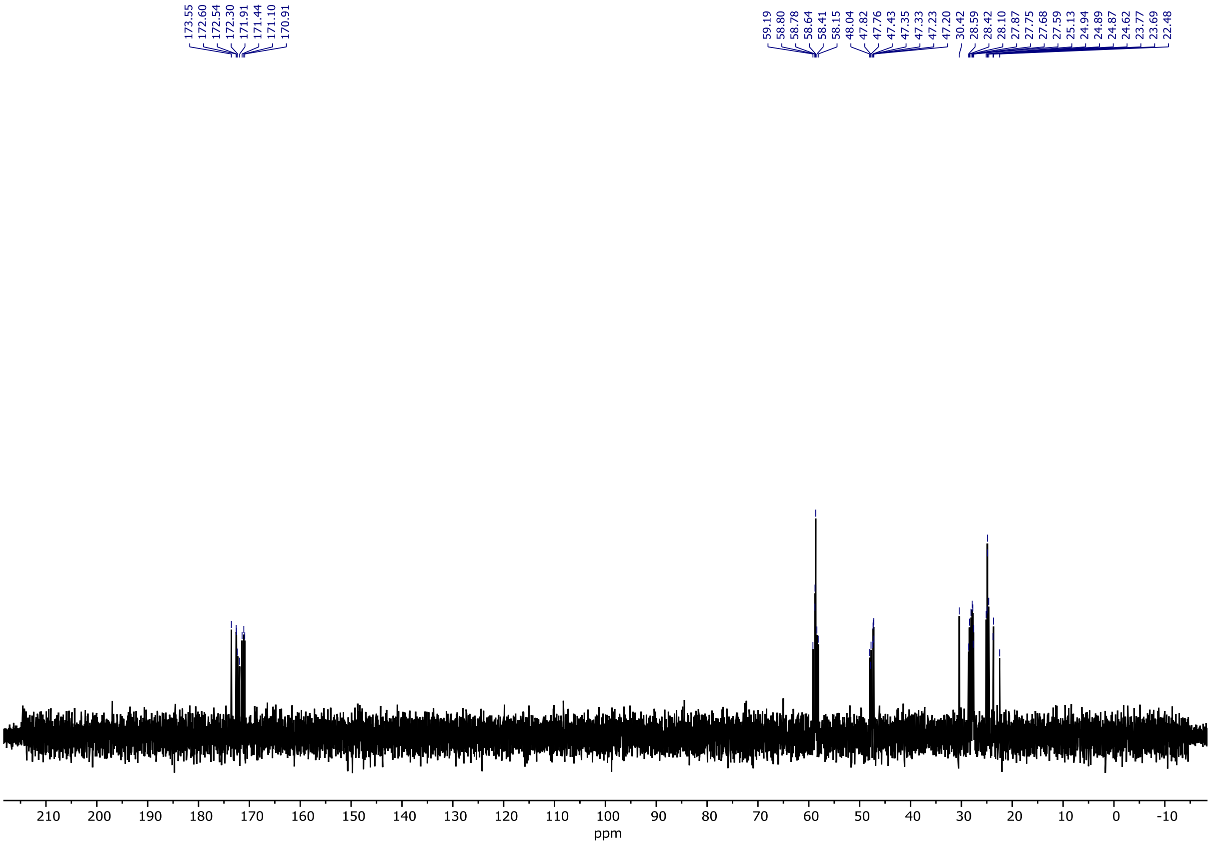


**Figure S24** $^{13}$C-NMR spectrum of **CP[4,4]** (151 MHz, $D_2O$, 298 K).

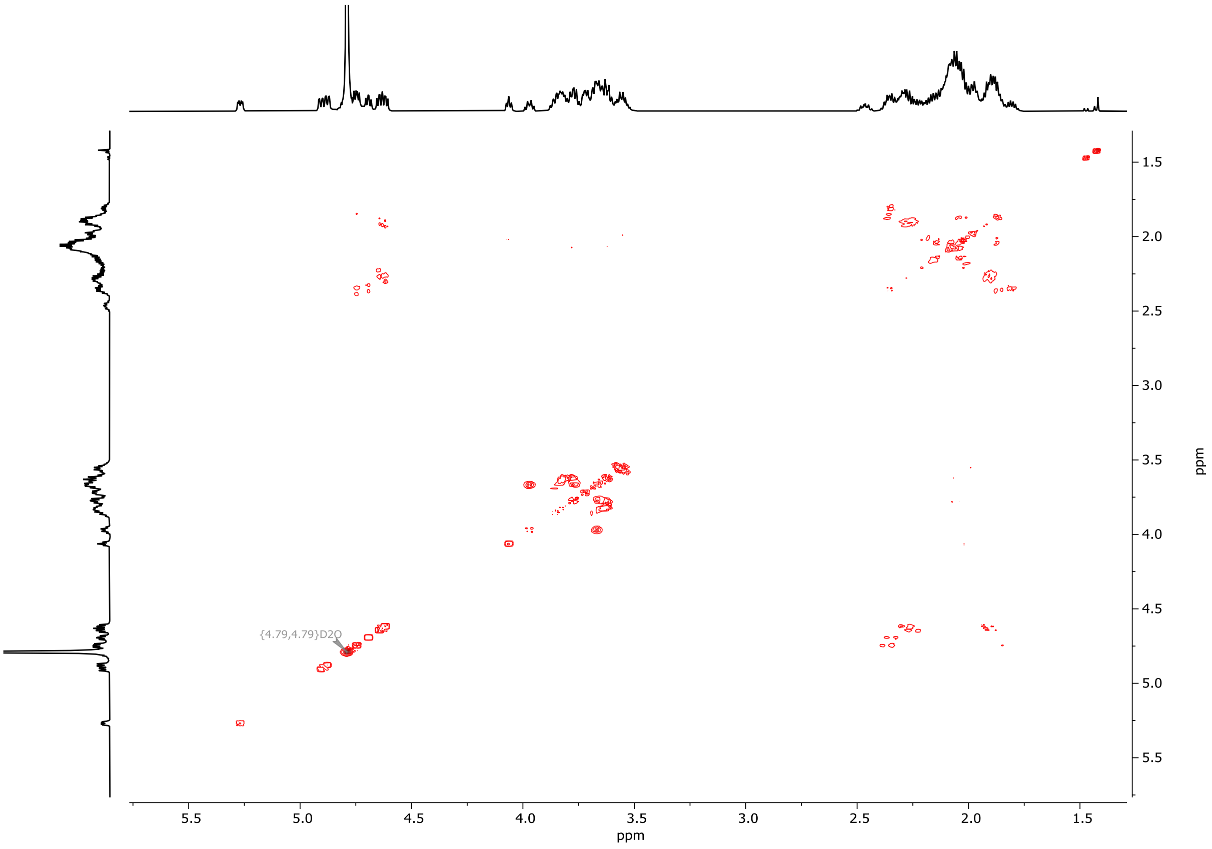


**Figure S25** $^{1}$H-$^{1}$H COSY NMR spectrum of **CP[4,4]** (600 MHz, $D_2O$, 298 K).

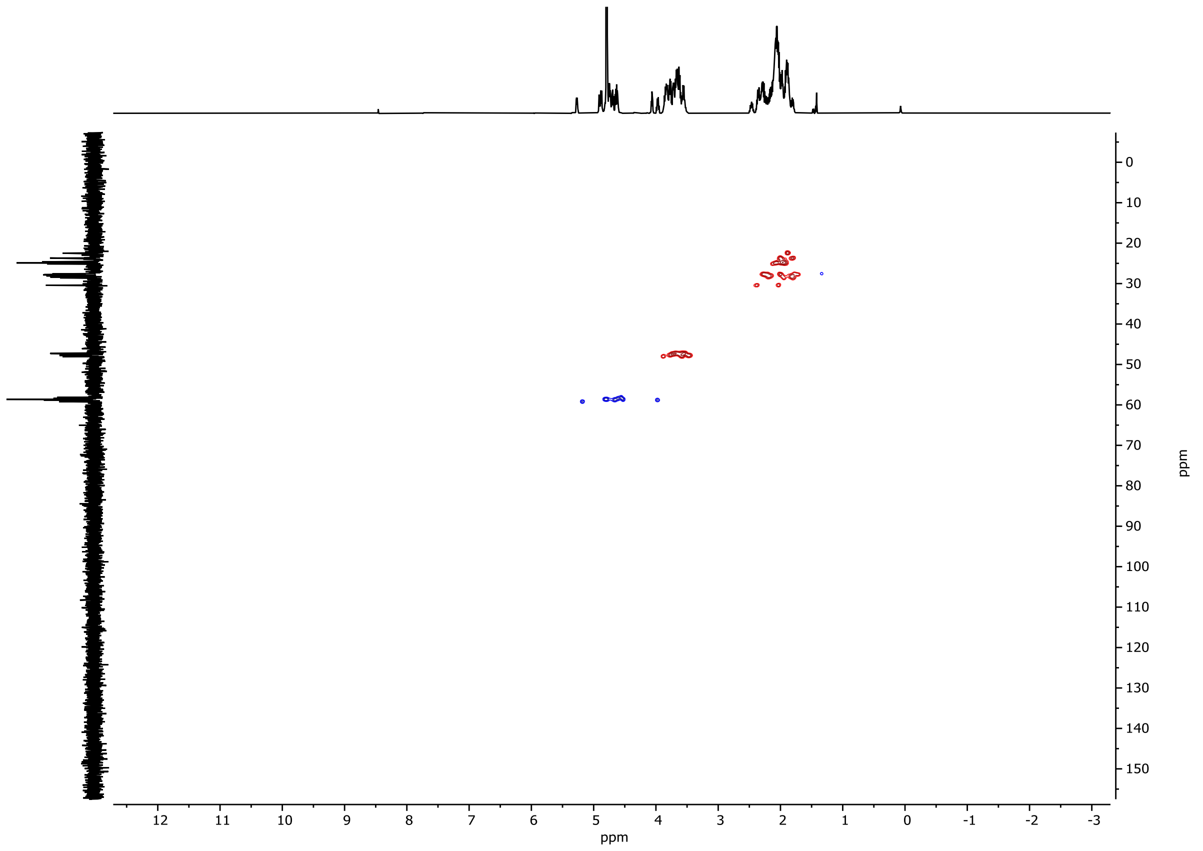


**Figure S26** $^{1}H$-$^{13}C$ HSQC NMR spectrum of **CP[4,4]** (600-151 MHz, $D_2O$, 298 K).

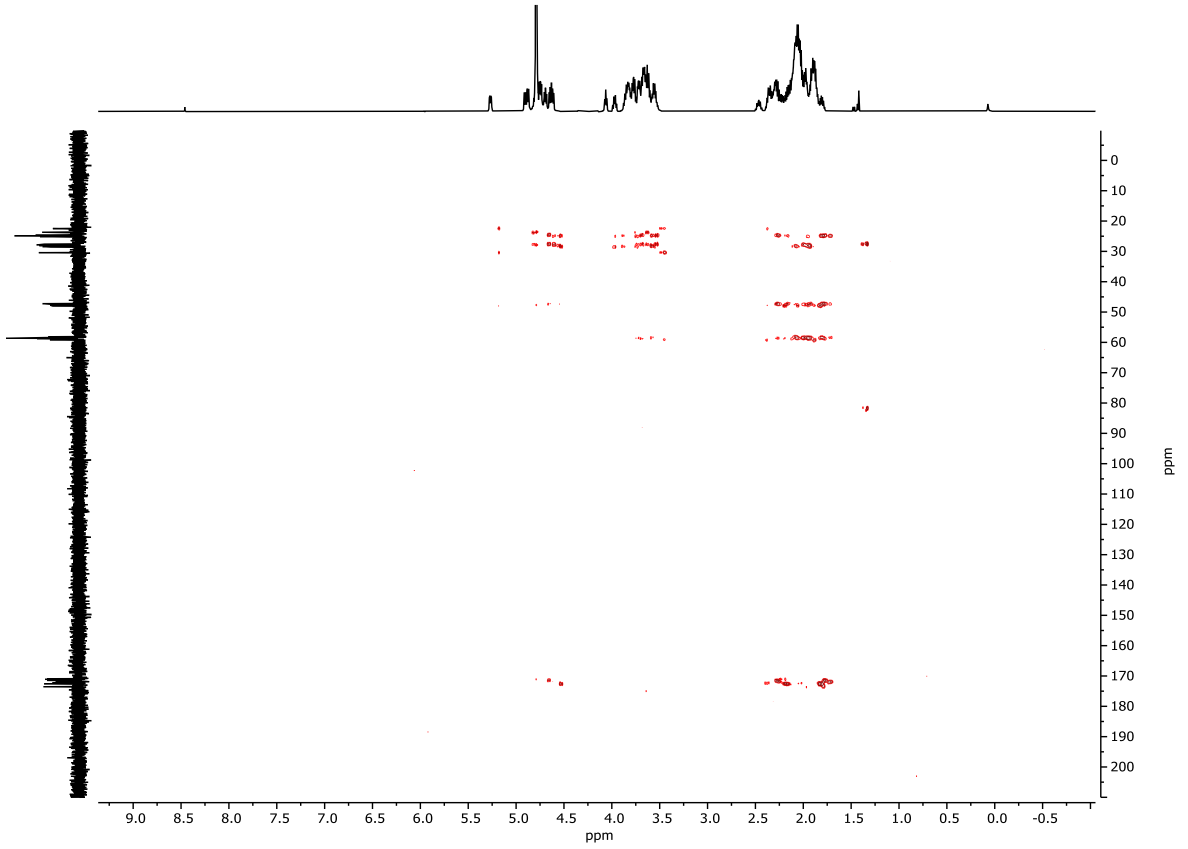


**Figure S27** $^{1}H$-$^{13}C$ HMBC NMR spectrum of **CP[4,4]** (600-151 MHz, $D_2O$, 298 K).

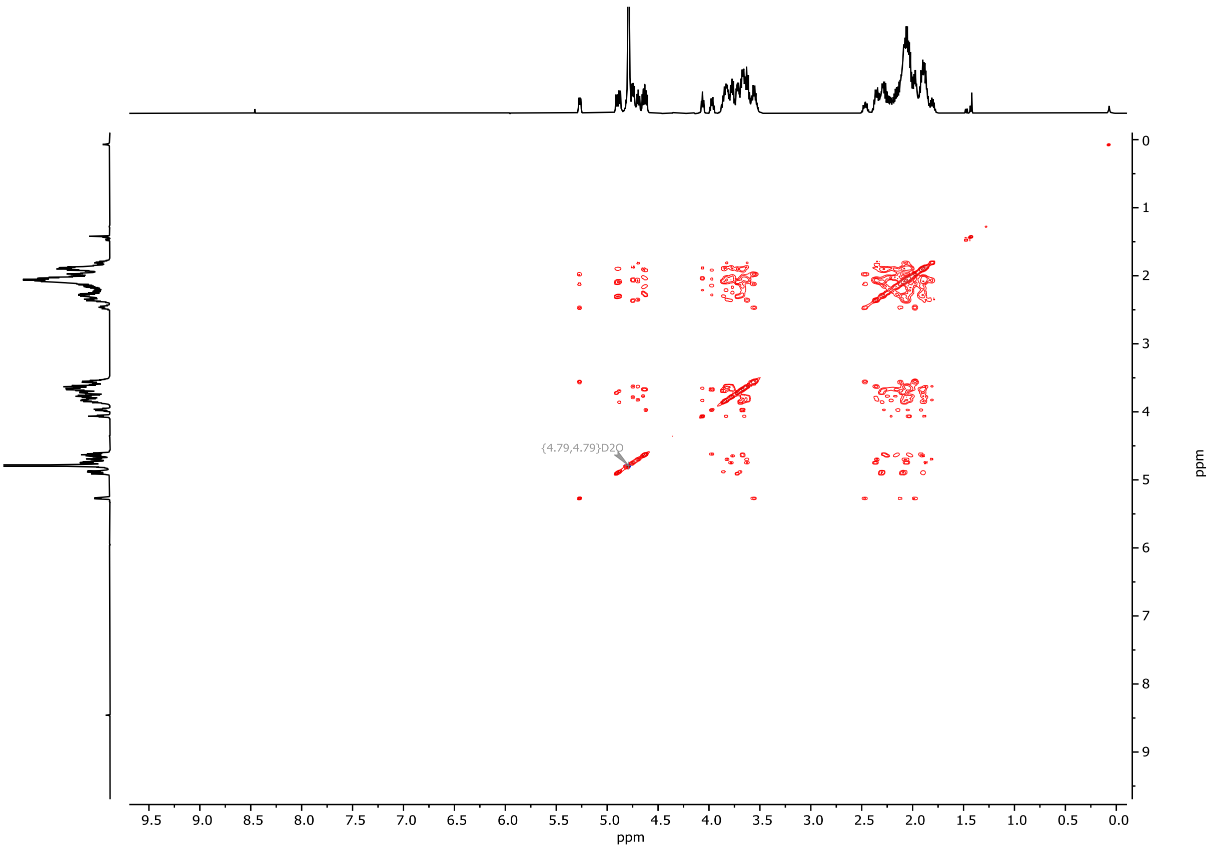


**Figure S28** $^{1}H$-$^{1}H$ TOCSY NMR spectrum of **CP[4,4]** (600 MHz, $D_2O$, 298 K).

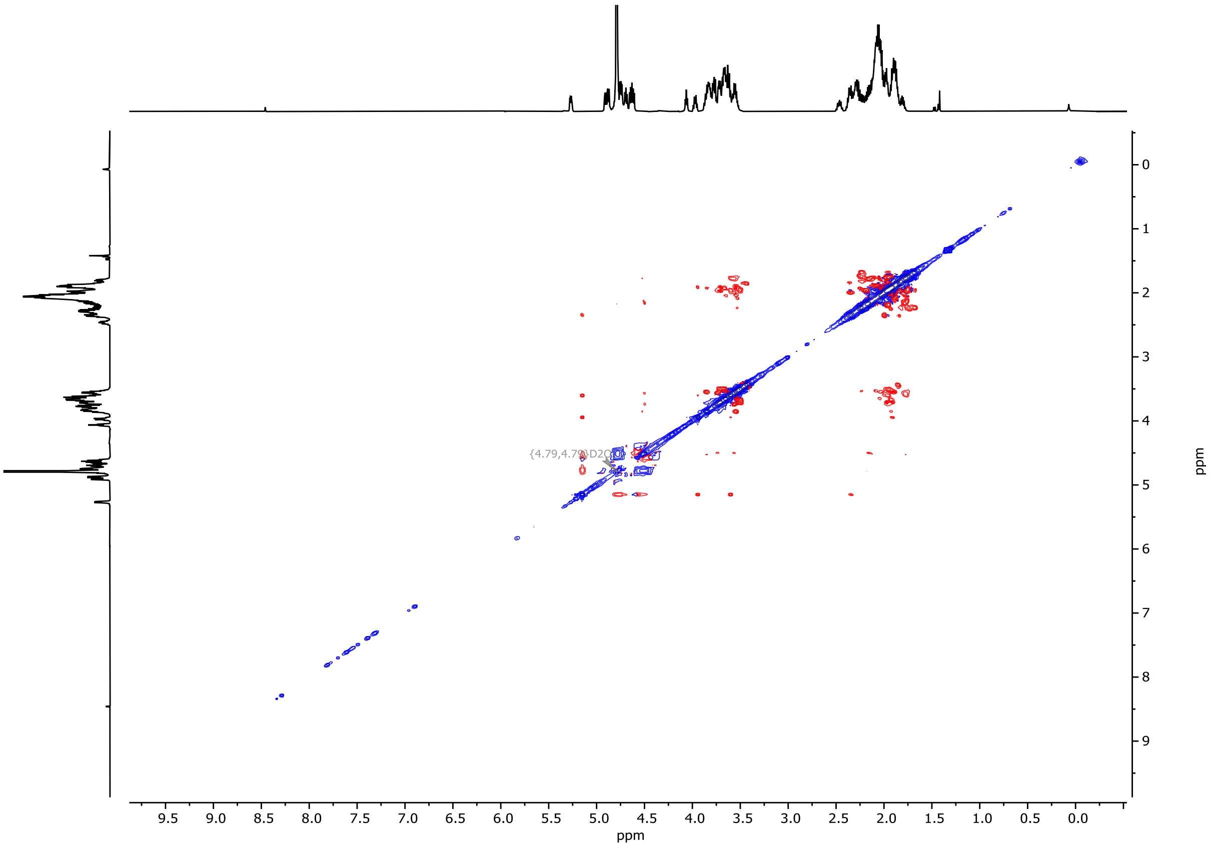


**Figure S29** $^{1}H$-$^{1}H$ ROESY NMR spectrum of **CP[4,4]** (600 MHz, $D_2O$, 298 K).

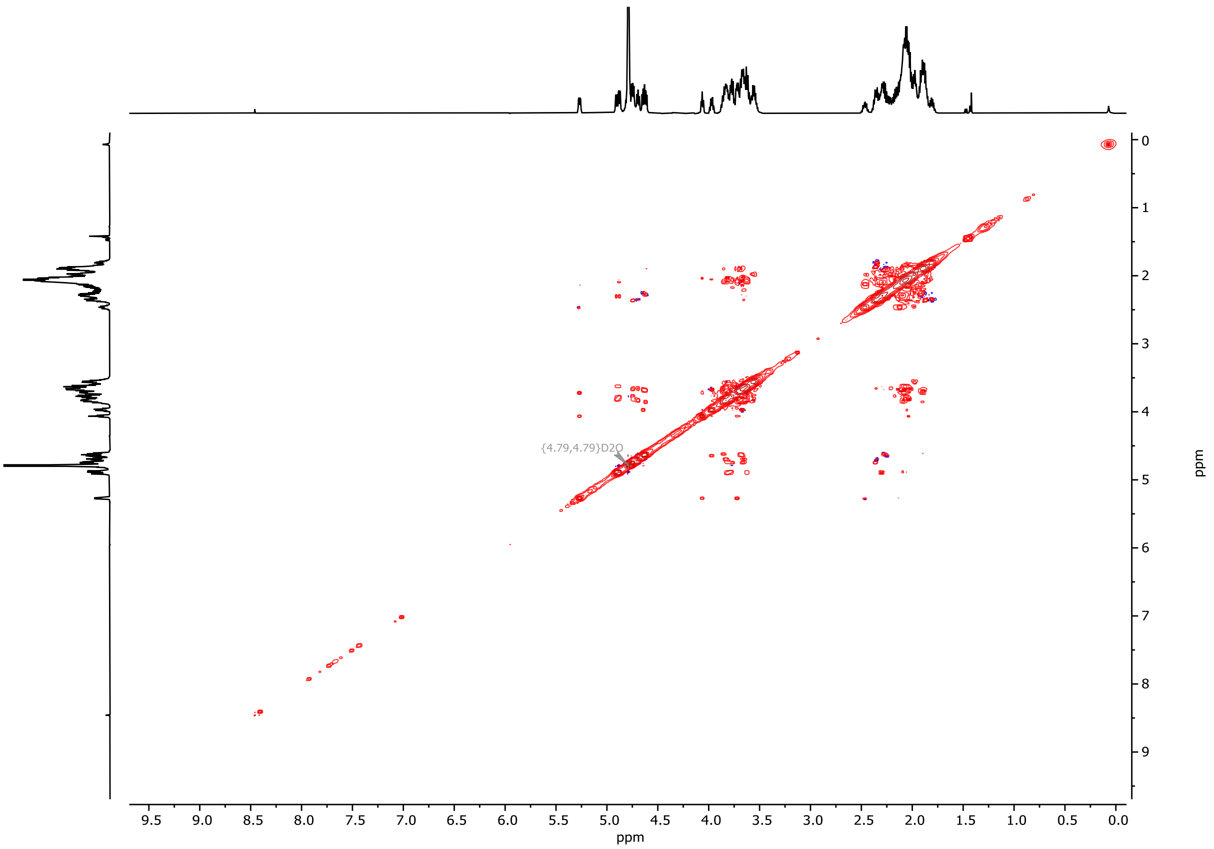


**Figure S30** $^1$H-$^1$H NOESY NMR spectrum of **CP[4,4]** (600 MHz, $D_2O$, 298 K).

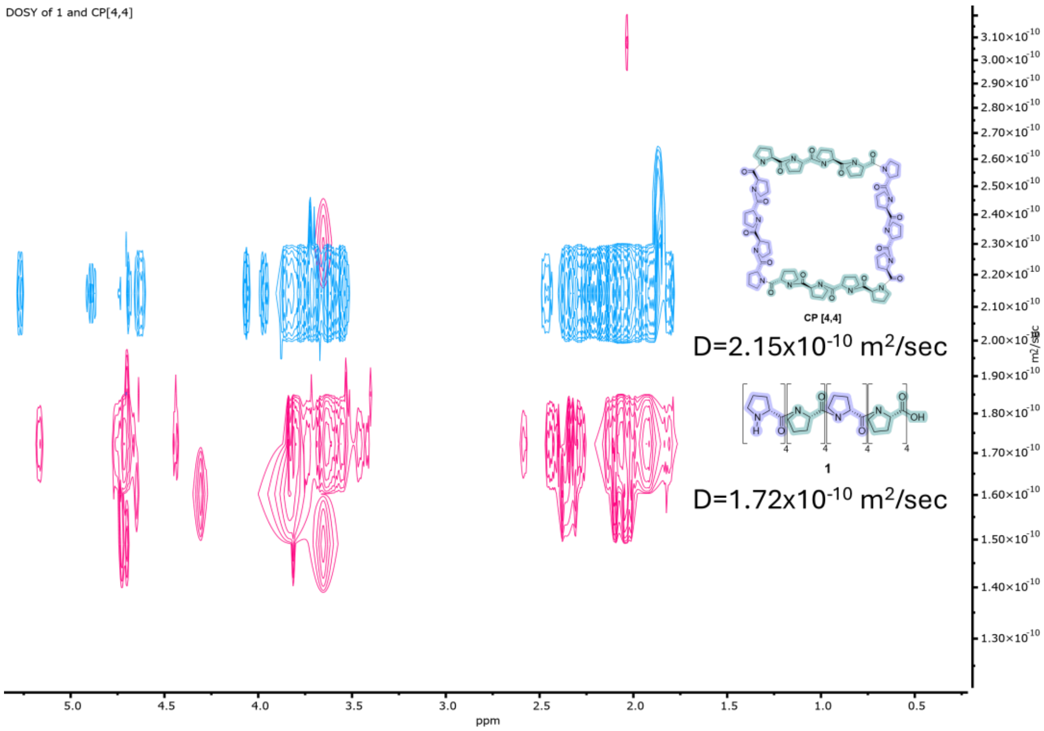


**Figure S31** $^1$H DOSY of **1** and **CP[4,4]** (600MHz, $D_2O$). DOSY *y*-axis scale is logarithmic.

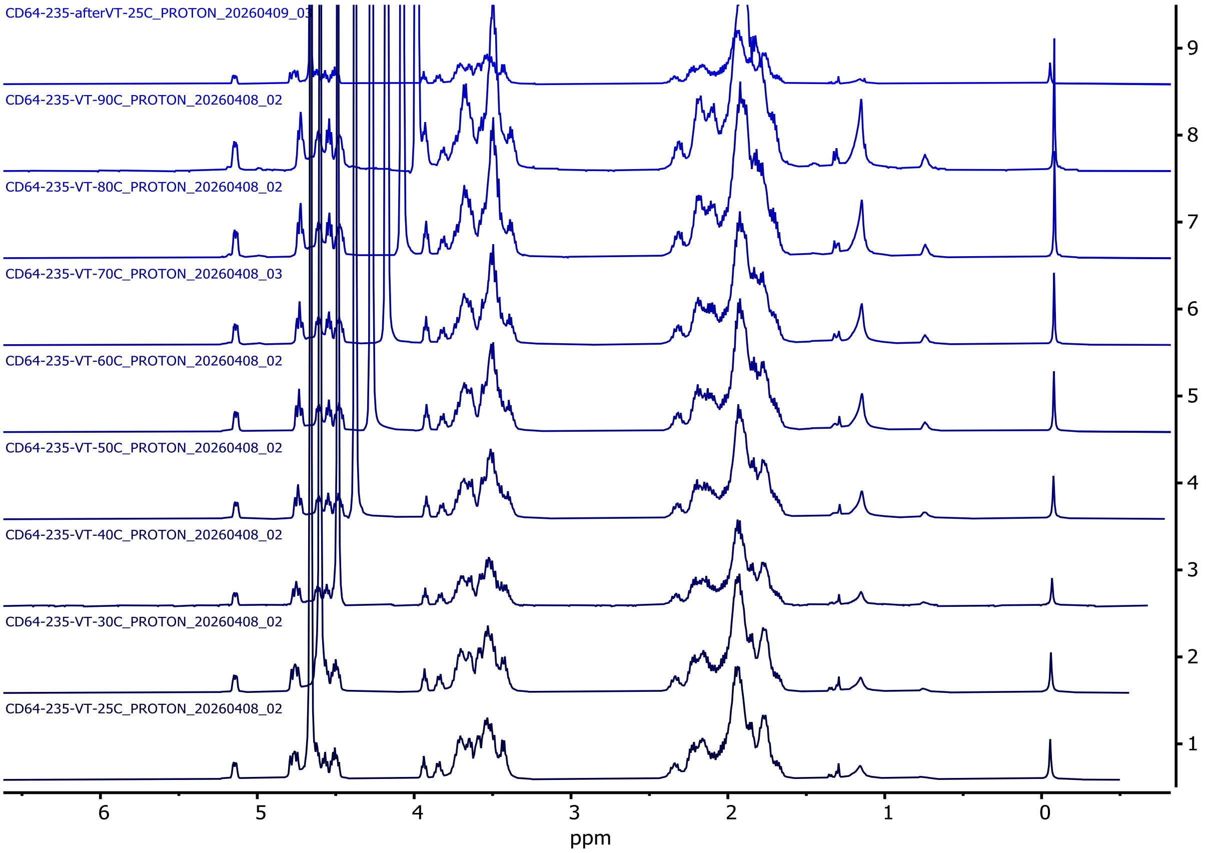


**Figure S32** Variable-temperature (VT) NMR analysis of **CP[4,4]** in $D_2O$. Spectrum 1 recorded at 298 K; Spectrum 2 at 303 K; Spectrum 3 at 313 K; Spectrum 4 at 323 K; Spectrum 5 at 333 K; Spectrum 6 at 343 K; Spectrum 7 at 353 K; Spectrum 8 at 363 K; Spectrum 9 at 298 K after recording spectrum 8.(500 MHz, $D_2O$, 298–363 K).

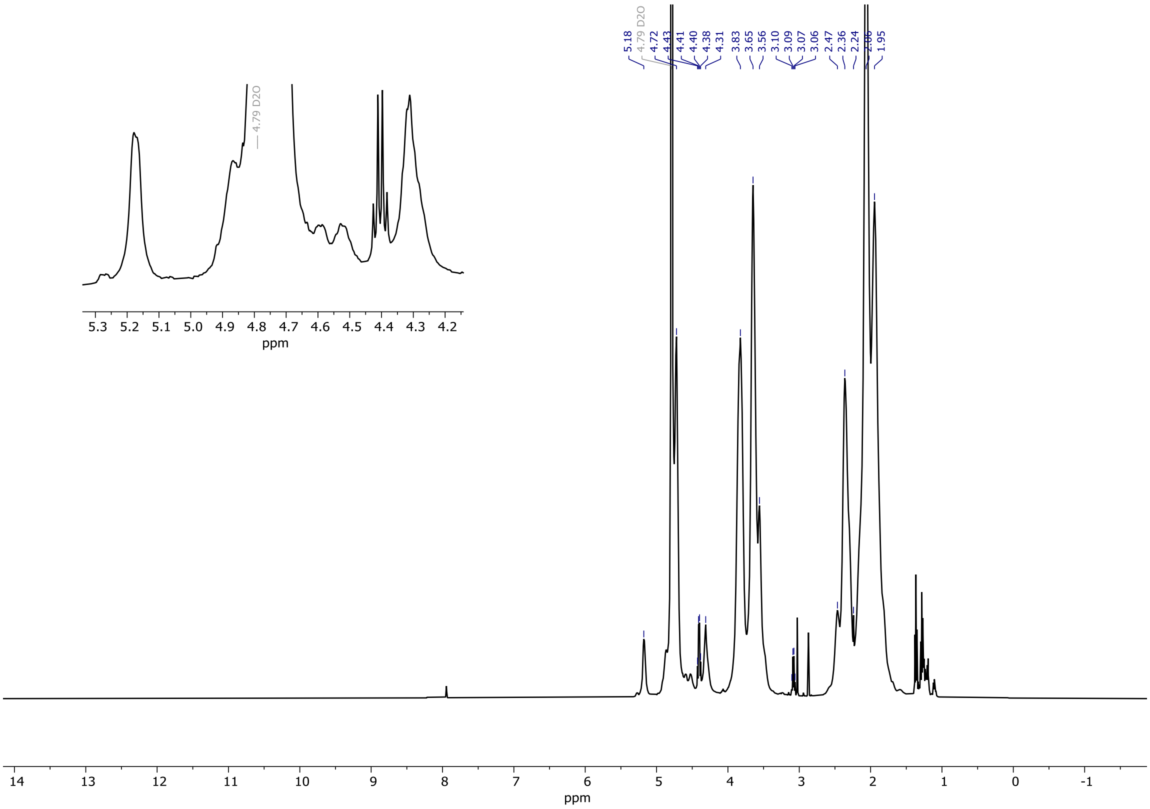


**FigureS33** $^{1}$H-NMR spectrum of crude **CP[4,4]**. DIC and Oxyma were used to perform the macrocyclization reaction, only oligomeric species are observed (500 MHz, $D_2O$, 298 K).

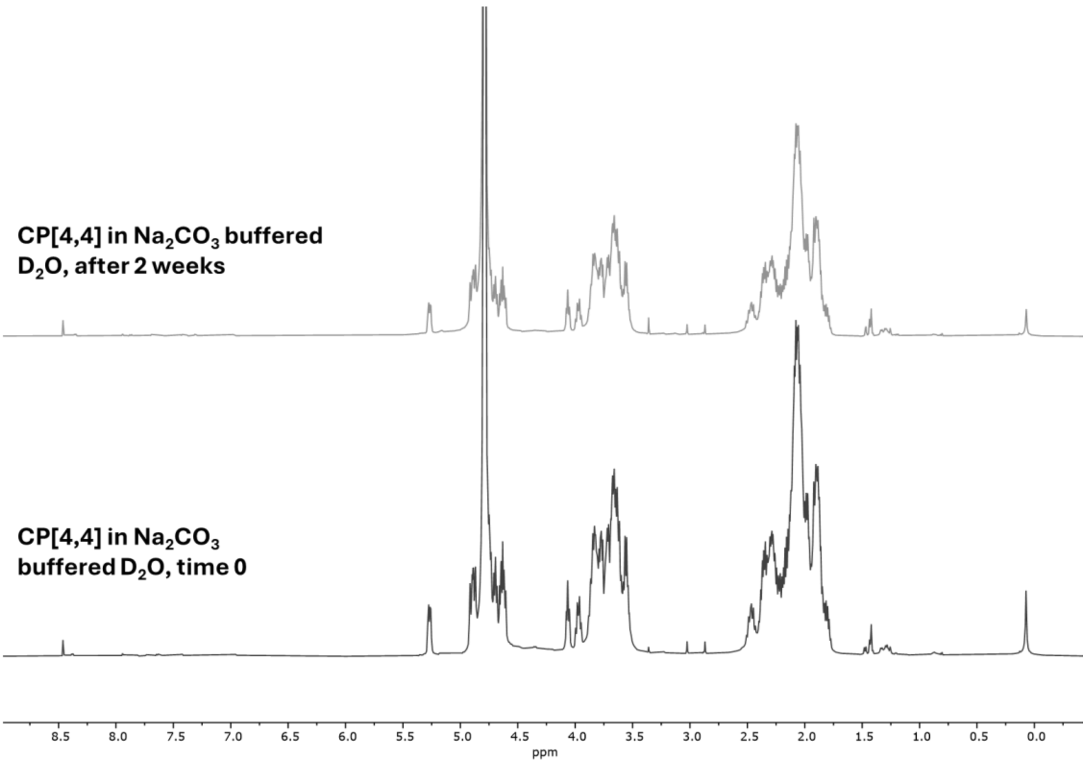


**Figure S34** Stacked $^{1}$H-NMRs of **CP[4,4]** in basic buffered $D_2O$ (to show its stability at pH = 11.6, 14 days at room temperature (500 MHz, $D_2O$, 298 K). To prepare this sample, 3mg of $Na_2CO_3$ were dissolved in 1ml with $D_2O$. This solution was used to prepare the 2.25mM solution of **CP[4,4]**.

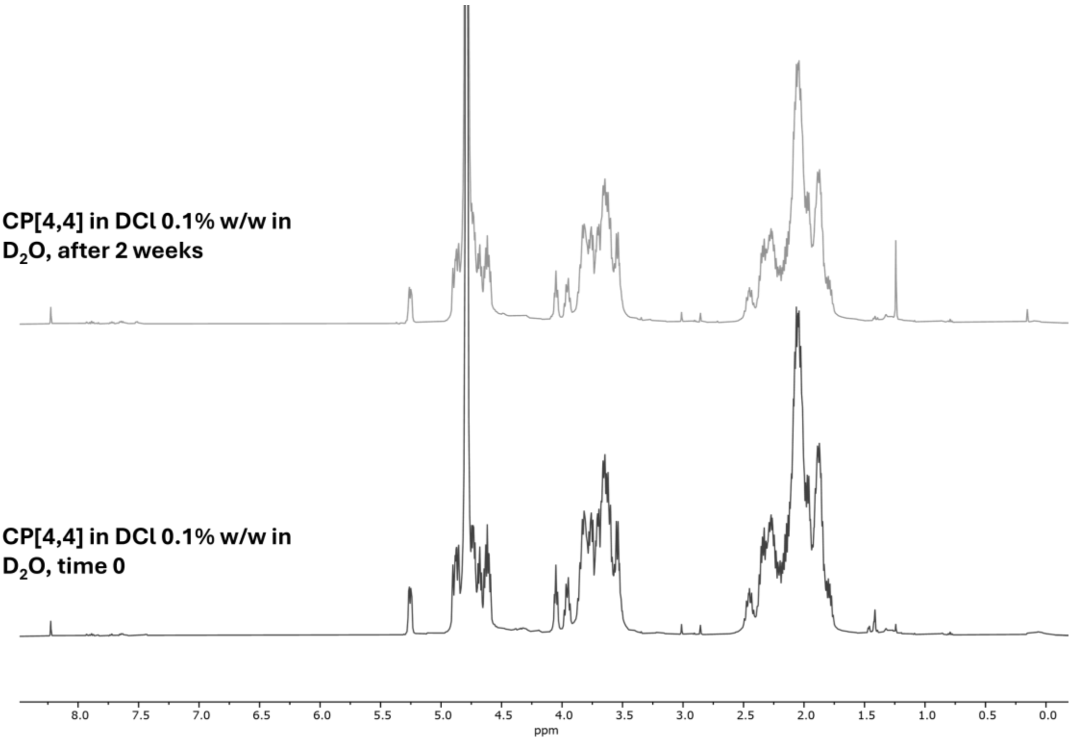


**Figure S35** Stacked $^{1}$H-NMRs of **CP[4,4]** in DCl 20% w/w in $D_2O$ to show its stability at pH = 0.80, 14 days at room temperature (500 MHz, $D_2O$, 298 K). To prepare this sample, 5μl of DCl/$D_2O$ 20% w/w was brought up to 1ml with $D_2O$. This solution (01% w/w final concentration of DCl) was used to prepare the 2.25mM solution of **CP[4,4]**.

# 4. NMR Titrations Data

**$^{1}$H-NMR Titration of $\mathbf{2}_{TFA}$ with CP[4,4]:** The $^1$H-NMR titration was performed at 298 K on a Bruker Avance Neo 600MHz spectrometer using $CDCl_3$ as solvent. A solution of the compound **$\mathbf{2}_{TFA}$**, the host (2.25mM), was titrated with successive additions of a solution (128.9 mM) of the guest in DMSO-$d_6$ (0.5-2.0 equiv). After each addition, the sample was homogenized before acquisition. Spectra were processed in TopSpin with manual phasing and baseline correction. For the titration of **$\mathbf{2}_{TFA}$** with **CP[4,4]**, the appearance of peaks in the all-junctions-*cis* **CP[4,4]** $^1$H-NMR (2.25mM in $CDCl_3$) at 4.70 ppm, 4.48 ppm, 4.37 ppm, 3.91 ppm, 3.85 ppm and 3.17 ppm upon addition of **$\mathbf{2}_{TFA}$** are diagnostic of the all-junctions-*trans* isomer. This result confirmed that titrating **$\mathbf{2}_{TFA}$** into a solution of all-junctions-*cis* **CP[4,4]** lead to the formation of the desired **CP[4,4]⊂$\mathbf{2}_{TFA}$** complex. The resulting apparent association constant is ~650 $M^{-1}$. It was calculated considering the integration of the peaks at 4.07 ppm and 4.40 ppm, respectively diagnostic of all-junctions-*cis* **CP[4,4]** (55%) and **CP[4,4]⊂$\mathbf{2}_{TFA}$** complex (45%), when 1 equivalent of the guest was added to the host solution.

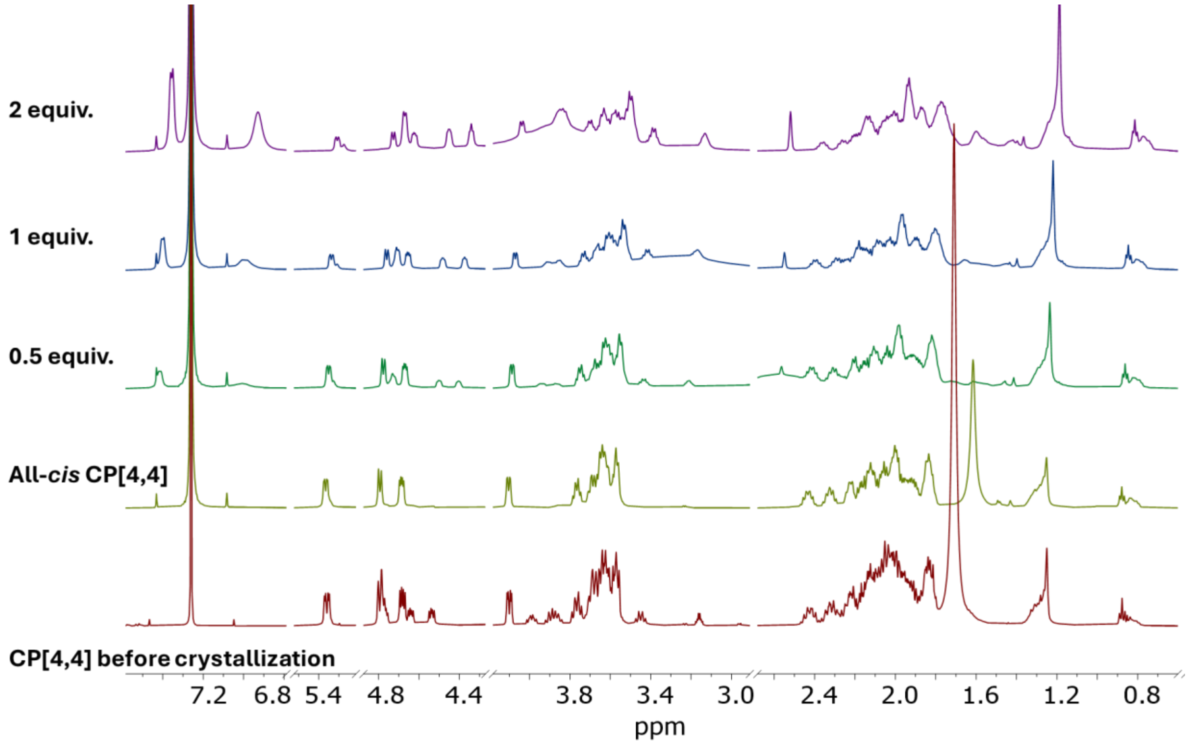


**Figure S36** Stack of $^1$H-NMRs showing the titration of **$\mathbf{2}_{TFA}$** with **CP[4,4]**. A new set of peaks, diagnostic of the successful formation of **CP[4,4]⊂$\mathbf{2}_{TFA}$** complex, is visible. The spectrum of **CP[4,4]** acquired before crystallization from $CDCl_3$, displaying a mixture of isomers, is included for clarity (600 MHz, $CDCl_3$, 298 K).

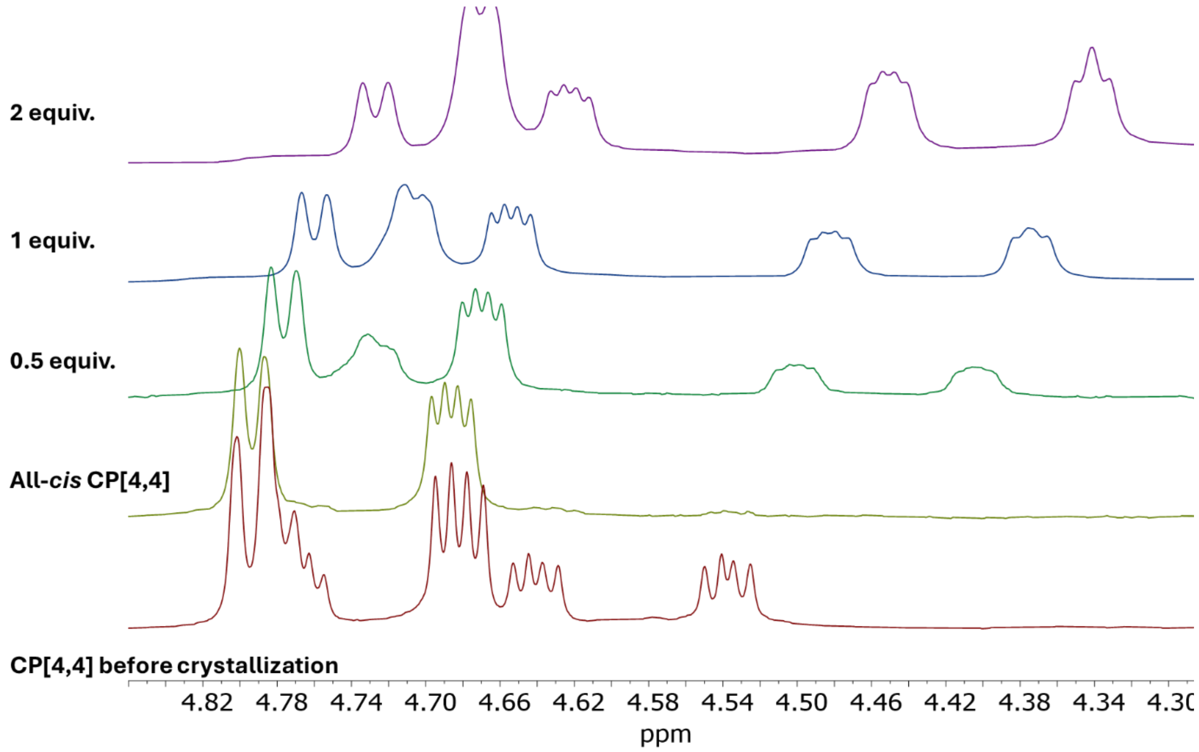


**Figure S37** Expansion of significant region of stacked $^{1}$H-NMRs showing titration of $\mathbf{2}_{TFA}$ with **CP[4,4]**. The spectrum of **CP[4,4]** acquired before crystallization from $CDCl_3$, displaying a mixture of isomers, is included for clarity (600 MHz, $CDCl_3$, 298 K).

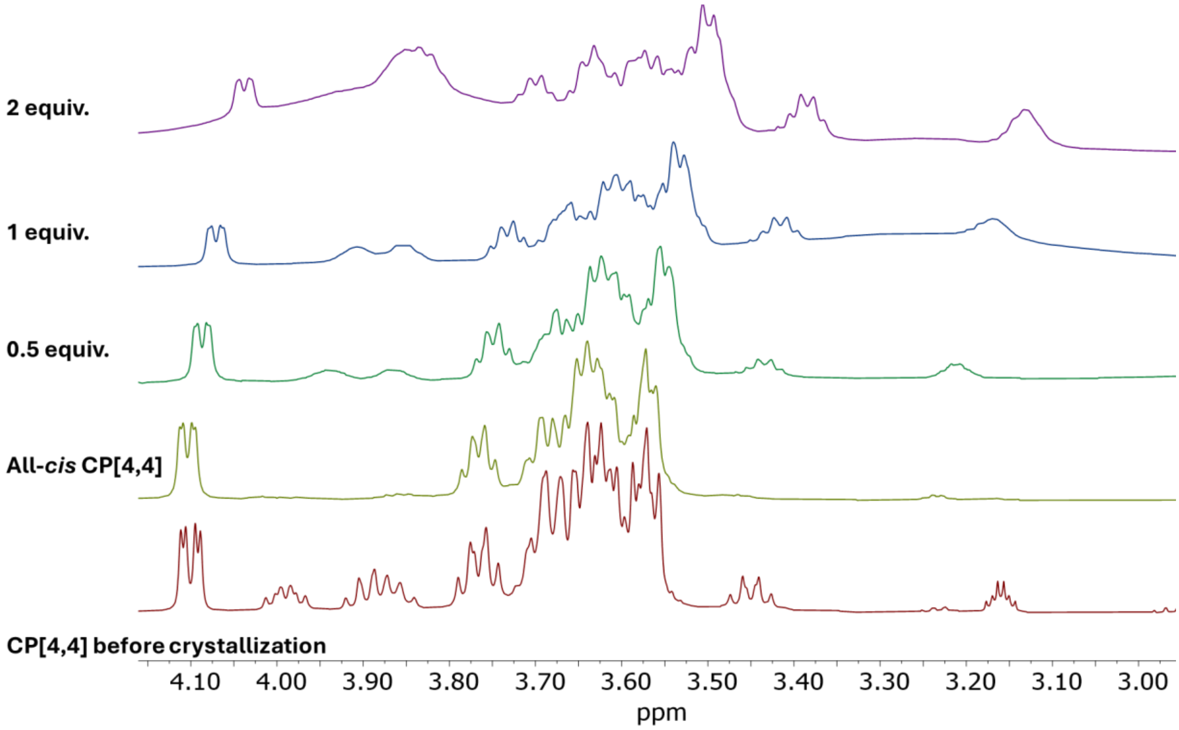


**Figure S38** Expansion of significant region of stacked $^{1}$H-NMR showing titration of $\mathbf{2}_{TFA}$ with **CP[4,4]**. The spectrum of **CP[4,4]** acquired before crystallization from $CDCl_3$, displaying a mixture of isomers, is included for clarity (600 MHz, $CDCl_3$, 298 K).

**$^{1}$H-NMR Titration of $2_{Cl}$ with CP[4,4]:** The $^{1}$H-NMR titration was performed at 298 K on a Bruker Avance Neo 600MHz spectrometer using $D_2O$ as solvent. A solution of the compound **$2_{Cl}$**, the host (2.25mM), was titrated with successive additions of a solution (128.6 mM) of the guest in $D_2O$ (1-12 equiv). After each addition, the sample was homogenized before acquisition. Spectra were processed in TopSpin with manual phasing and baseline correction. For the titration of **$2_{Cl}$** with **CP[4,4]**, the chemical shift change (Δδ) for the α-proton stand-alone peak (4.63ppm → 4.49ppm ) was monitored and fitted to a 1:1 binding model using the BindFit web tool. The resulting apparent association constant of 70.82±3.99% $M^{-1}$ was found.

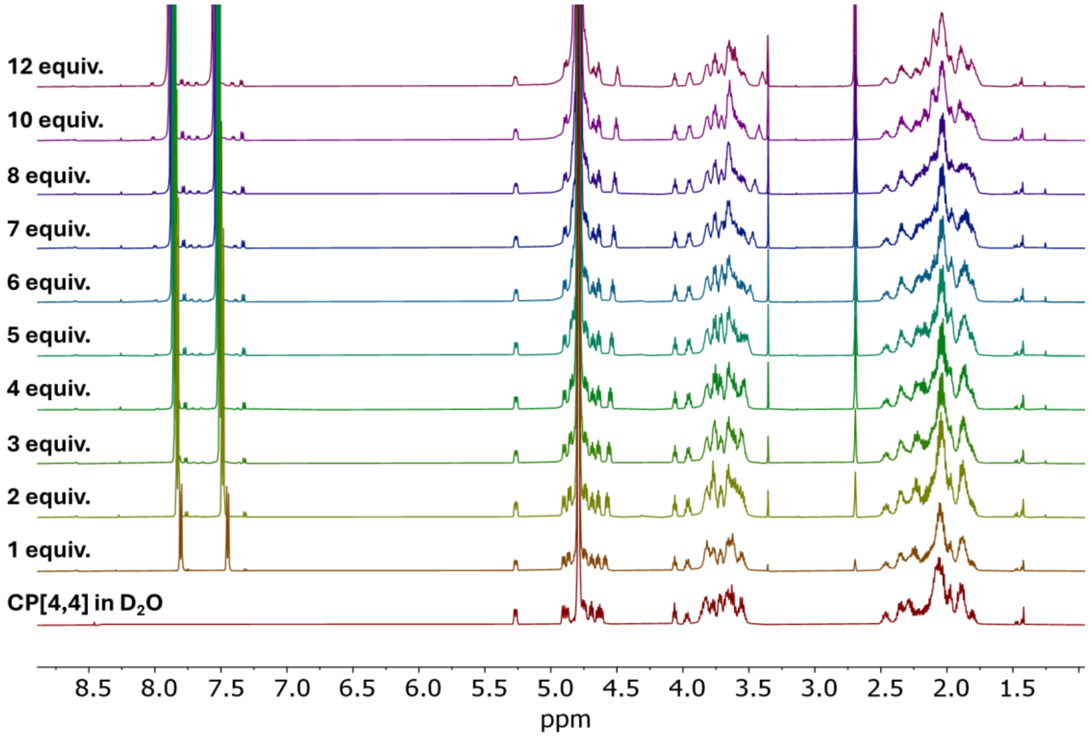


**Figure S39** Overview of stacked $^{1}$H-NMRs showing titration of **$2_{Cl}$** with **CP[4,4]** in $D_2O$ (600 MHz, $D_2O$, 298 K).

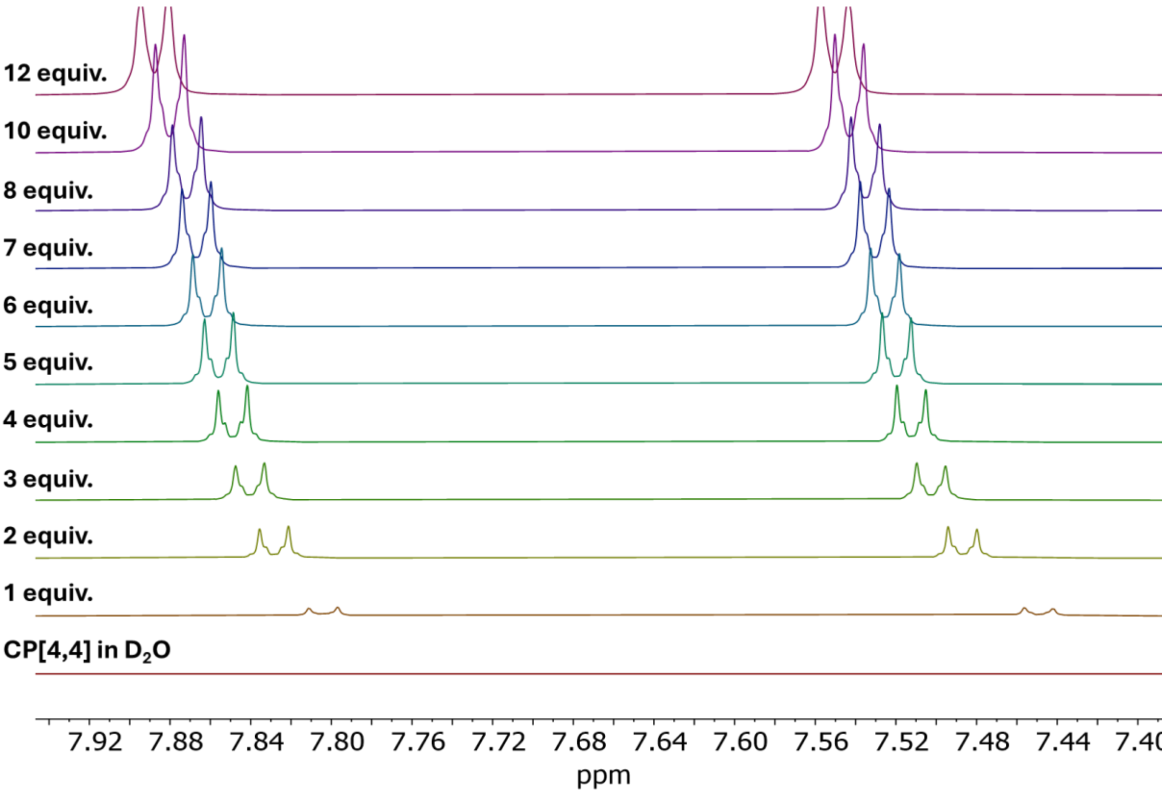


**Figure S40** Expansion of stacked $^{1}$H-NMRs showing titration of **2**$_{Cl}$ with **CP[4,4]** in $D_2O$ showing a significant region of shifting peaks (600 MHz, $D_2O$, 298 K).

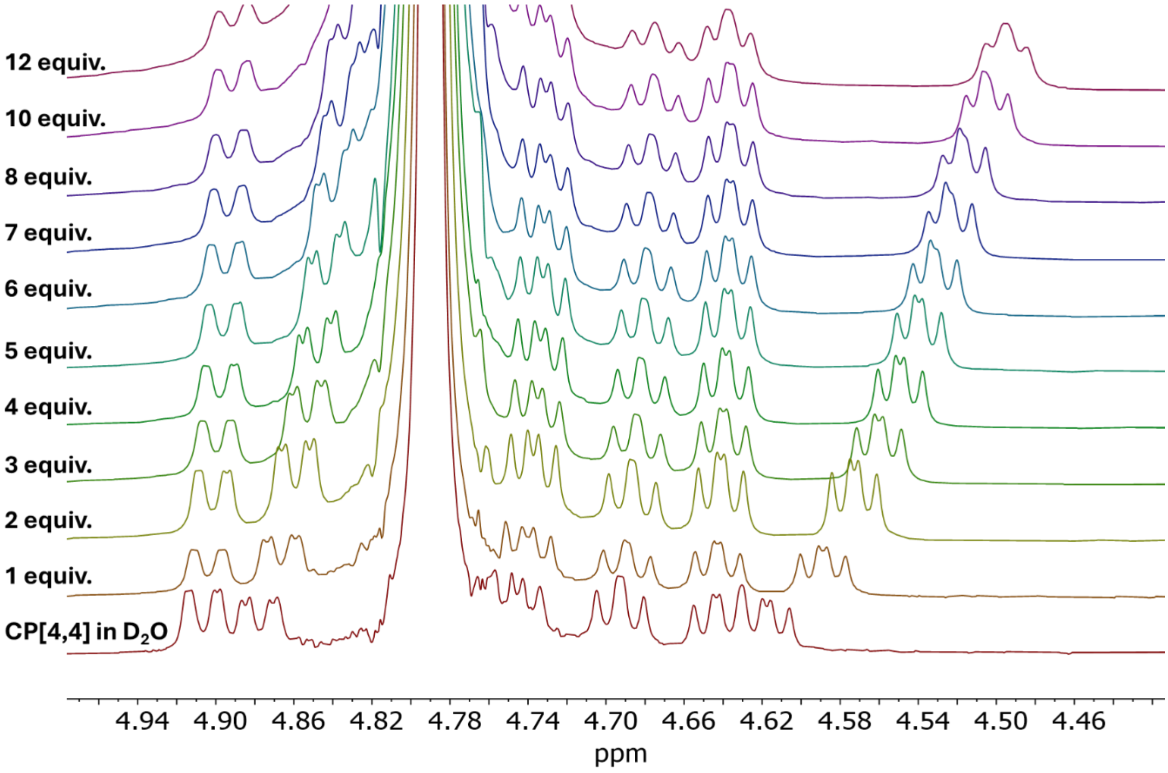


**Figure S41** Expansion of a significant region of stacked $^{1}$H-NMRs showing titration of **2**$_{Cl}$ with **CP[4,4]** in $D_2O$ where the shifting stand-alone peak (4.63 ppm → 4.49 ppm) was the one used to calculate the binding affinity value (600 MHz, $D_2O$, 298 K).

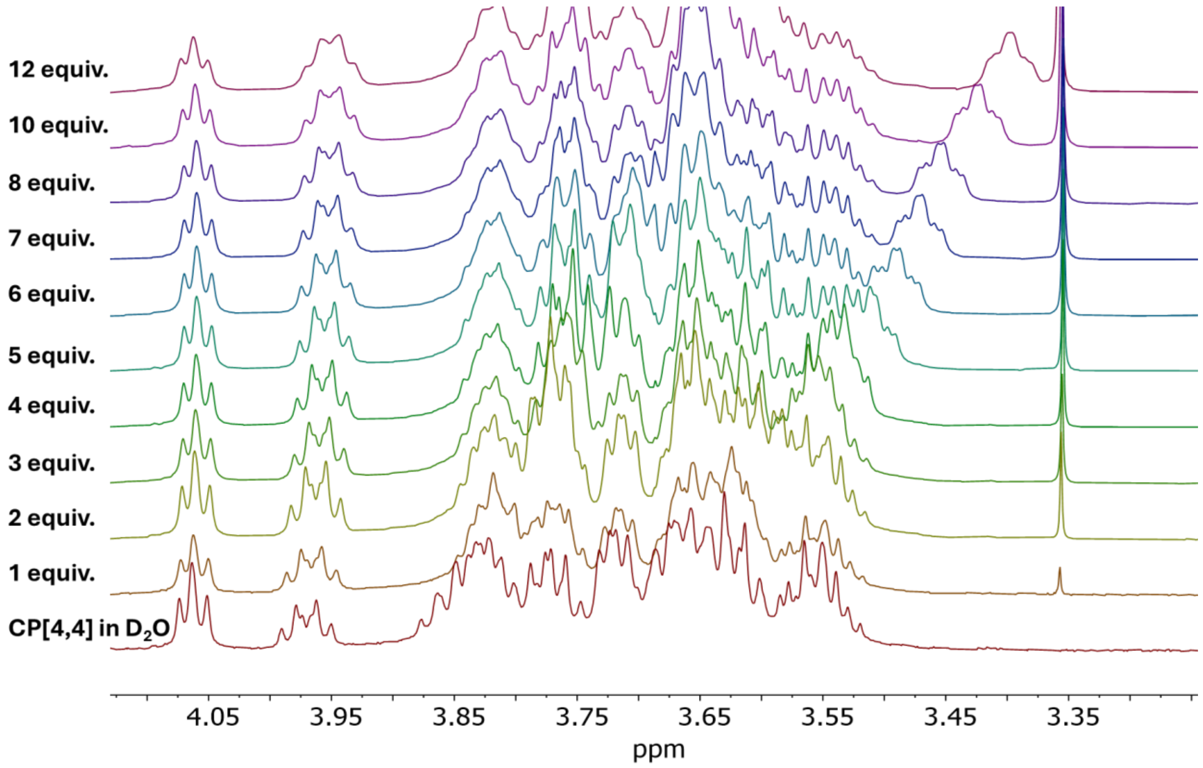


**Figure S42** Expansion of a significant region in the stacked $^{1}$H-NMRs of the titration of $\mathbf{2}_{Cl}$ with **CP[4,4]** in $D_2O$ where some shifting peaks are visible (600 MHz, $D_2O$, 298 K).

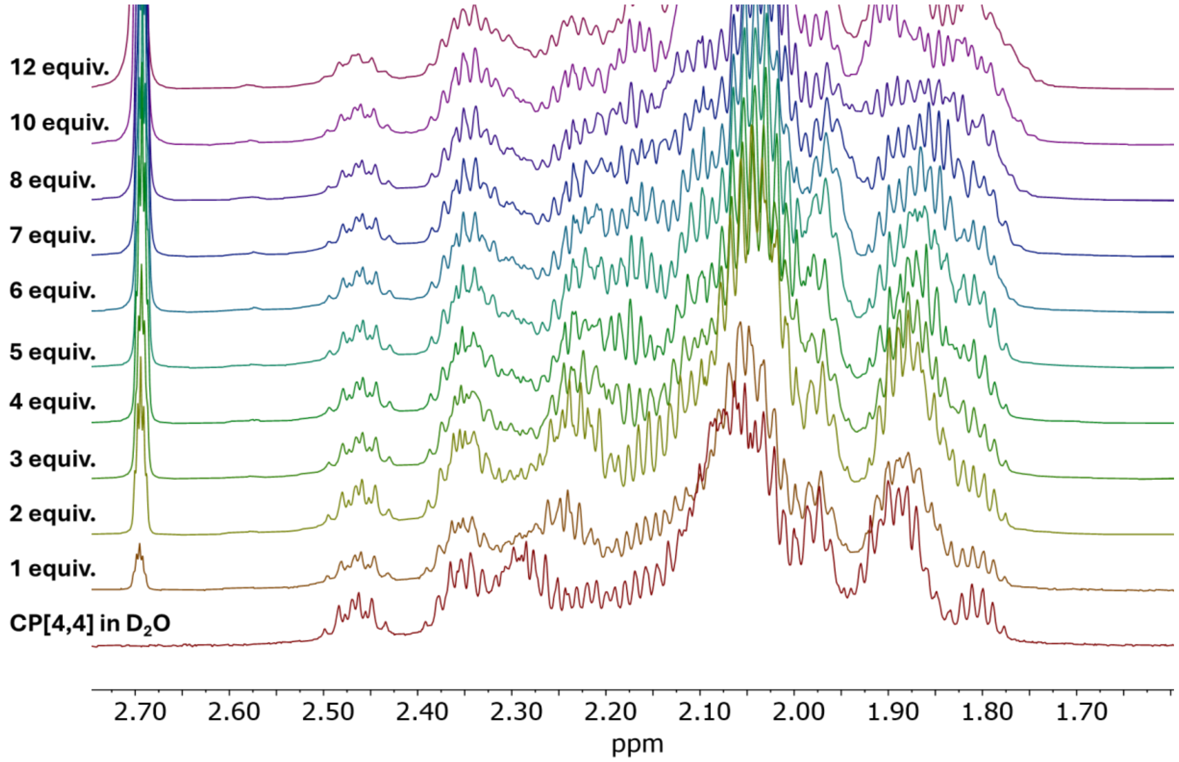


**Figure S43** Expansion of stacked $^{1}$H-NMRs of the titration of $\mathbf{2}_{Cl}$ with **CP[4,4]** in $D_2O$ showing a significant and complex region of shifting peaks (600 MHz, $D_2O$, 298 K).

**$^1$H-NMR Titration of 3 with CP[4,4]:** $^1$H-NMR one-point titration was performed at 298 K on a Bruker Avance Neo 600MHz spectrometer using $D_2O$ as solvent. A solution of **CP[4,4]** (2.37 mM) was titrated adding one aliquot (1 eq) of a 226.6 mM solution of the guest **3** in DMSO-$d_6$. After the addition, the sample was homogenized before acquisition. Spectra were processed in TopSpin with manual phasing and baseline correction.

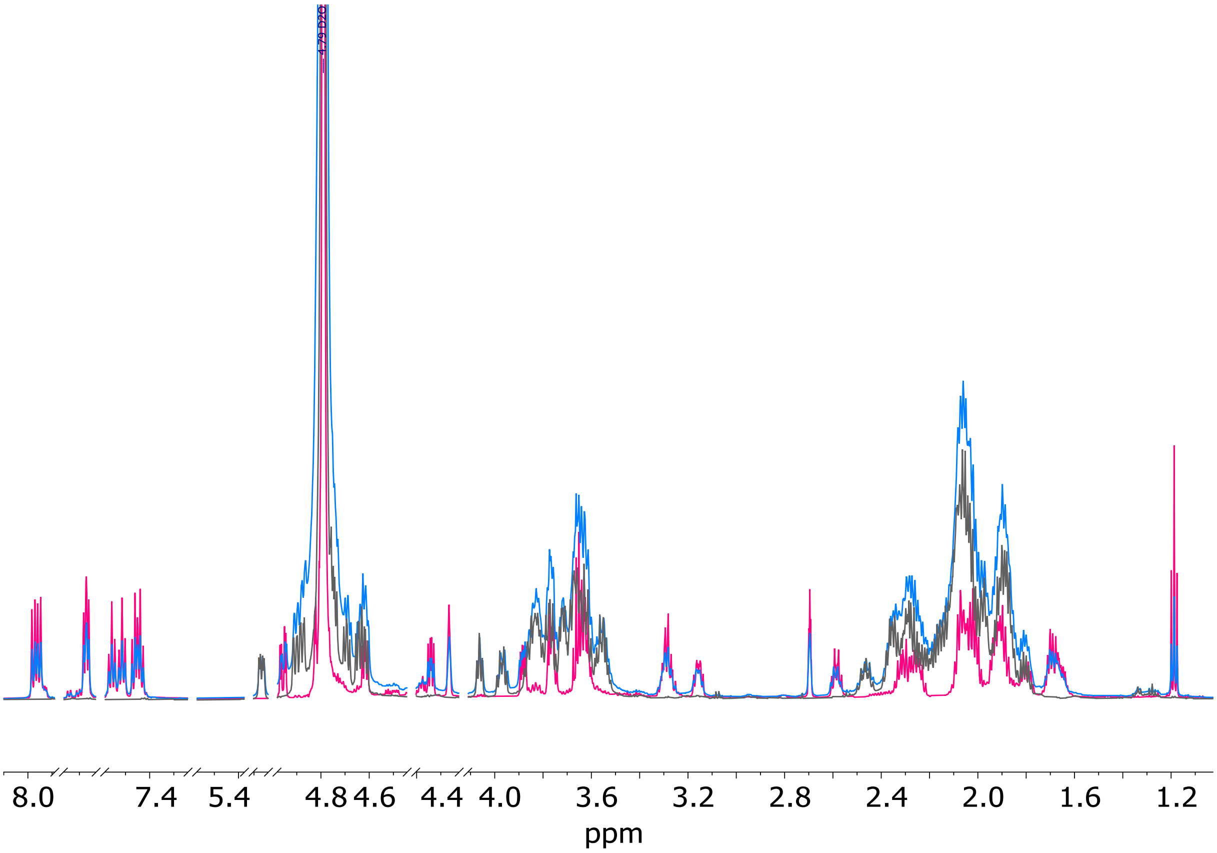


**Figure S44** Overlay of $^1$H-NMRs, **3** (pink trace), **CP[4,4]** (grey trace) and a 1:1 mixture of the **3** and **CP[4,4]** (blue trace). All the spectra are acquired in $D_2O$ (600 MHz, $D_2O$, 298 K).

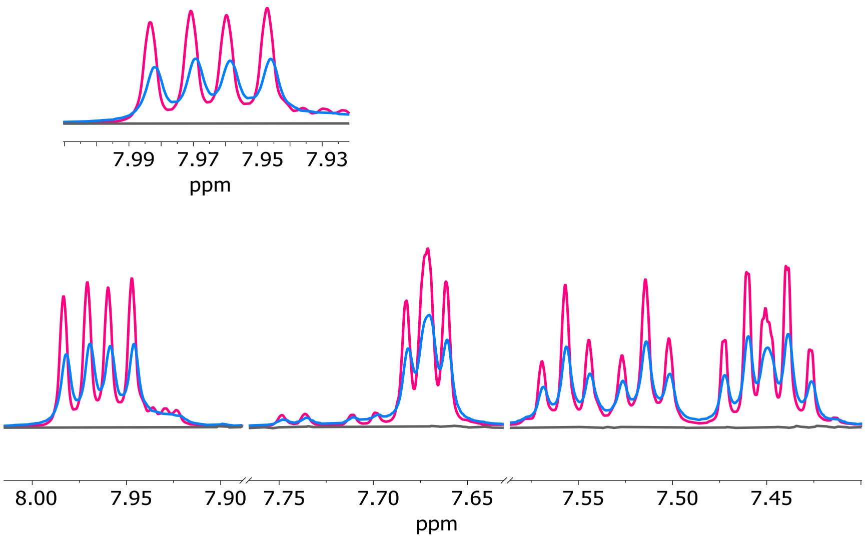


**Figure S45** Expansion of a region of overlaid $^1$H-NMRs, **3** (pink trace), **CP[4,4]** (grey trace – as expected no peaks in the aromatic region are observed) and a 1:1 mixture of the **3** and **CP[4,4]** (blue trace). All the spectra were acquired in $D_2O$ (600 MHz, $D_2O$, 298 K).

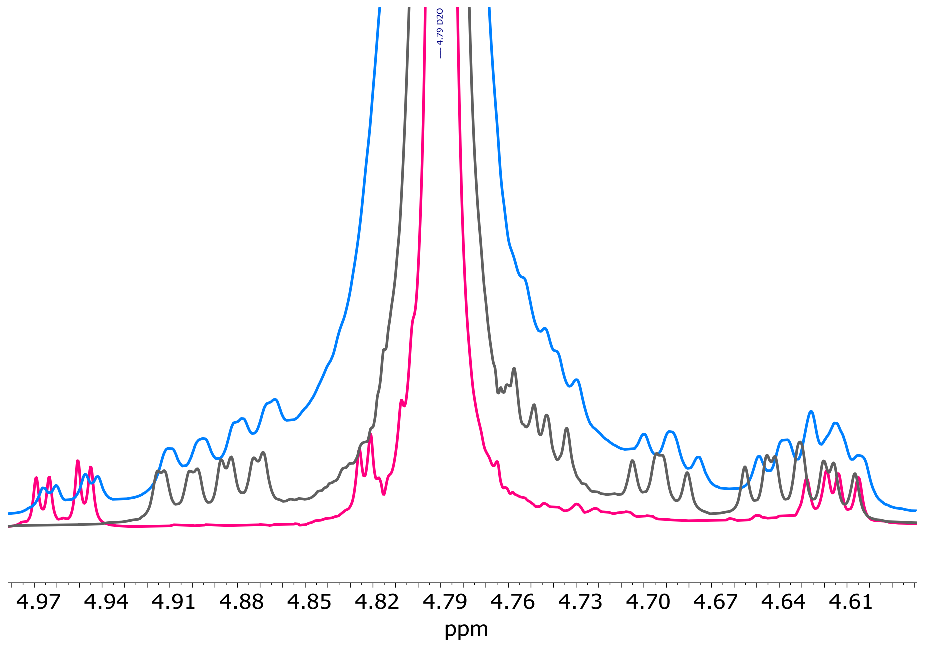


**Figure S46** Expansion of a region of the overlay of $^{1}$H-NMRs of **3** (pink trace), **CP[4,4]** (grey trace) and a 1:1 mixture of the **3** and **CP[4,4]** (blue trace). All the spectra were acquired in $D_2O$ (600 MHz, $D_2O$, 298 K).

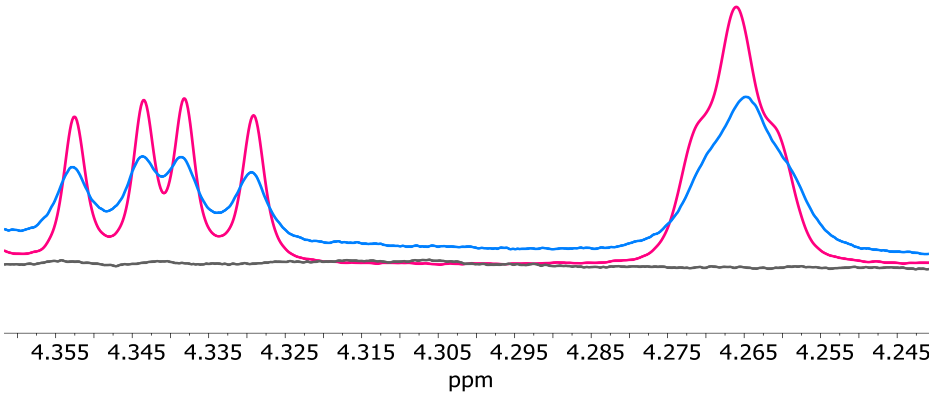


**Figure S47** Expansion of a region of overlaid $^{1}$H-NMRs, **3** (pink trace), **CP[4,4]** (grey trace) and a 1:1 mixture of the **3** and **CP[4,4]** (blue trace). All the spectra were acquired in $D_2O$ (600 MHz, $D_2O$, 298 K).

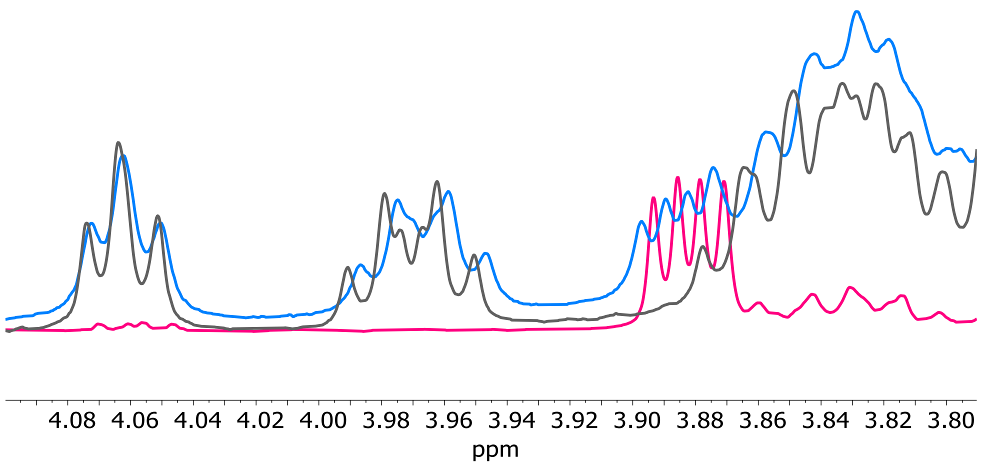


**Figure S48** Expansion of a region of overlaid $^{1}$H-NMRs, **3** (pink trace), **CP[4,4]** (grey trace) and a 1:1 mixture of the **3** and **CP[4,4]** (blue trace). All the spectra were acquired in $D_2O$ (600 MHz, $D_2O$, 298 K).

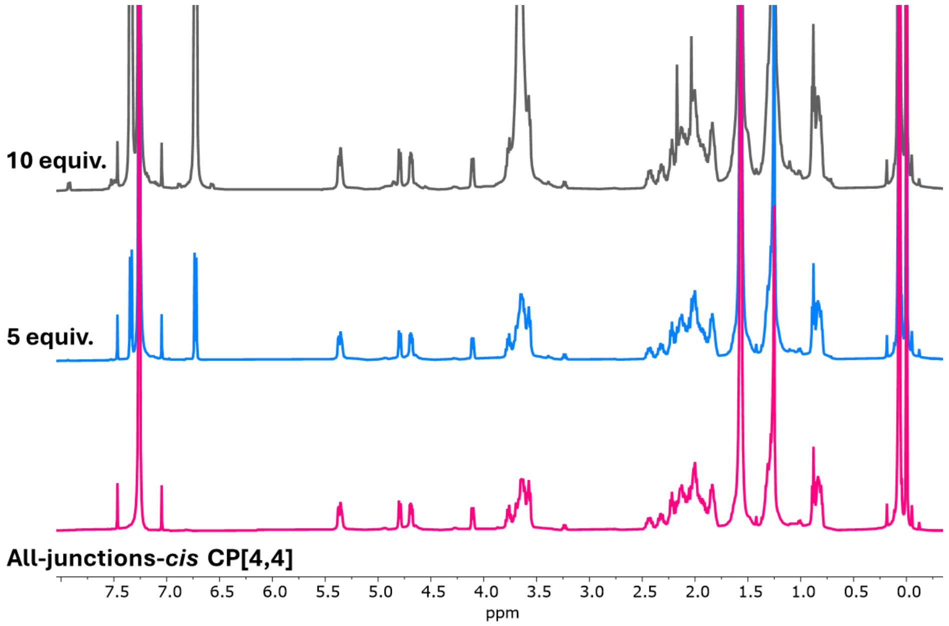


**Figure S49** $^{1}$H-NMR stack of all-junctions-*cis* **CP[4,4]** with unprotonated benzidine **2** (5 and 10 equivalents) showing no induced-fit complexation (500MHz, $CDCl_3$, 298k).

# 5. LC-HRMS Data

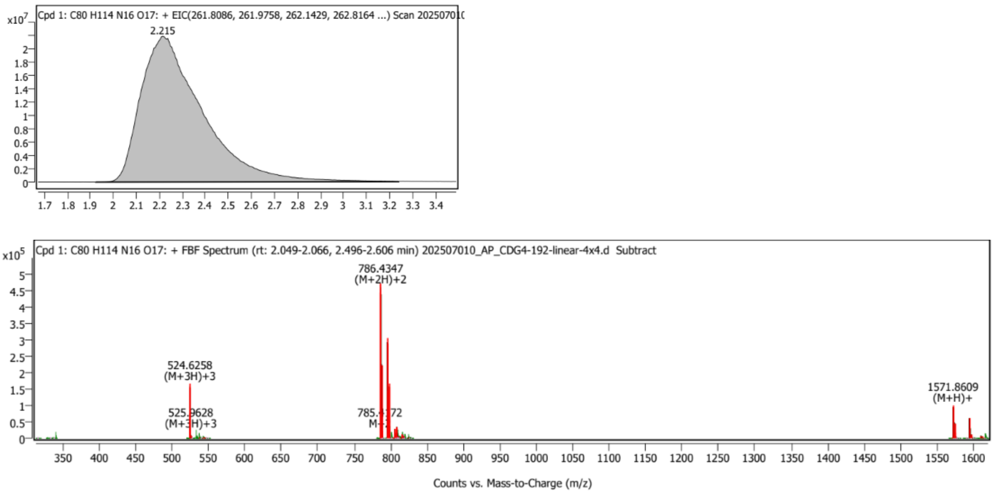


**Figure S50** EIC and HRMS and HRMS for **1**; HRMS: calcd for $[M+H]^+$: $C_{80}H_{114}N_{16}O_{17}$, 1571.8581; found; $[M+H]^+$ 1571.8609.

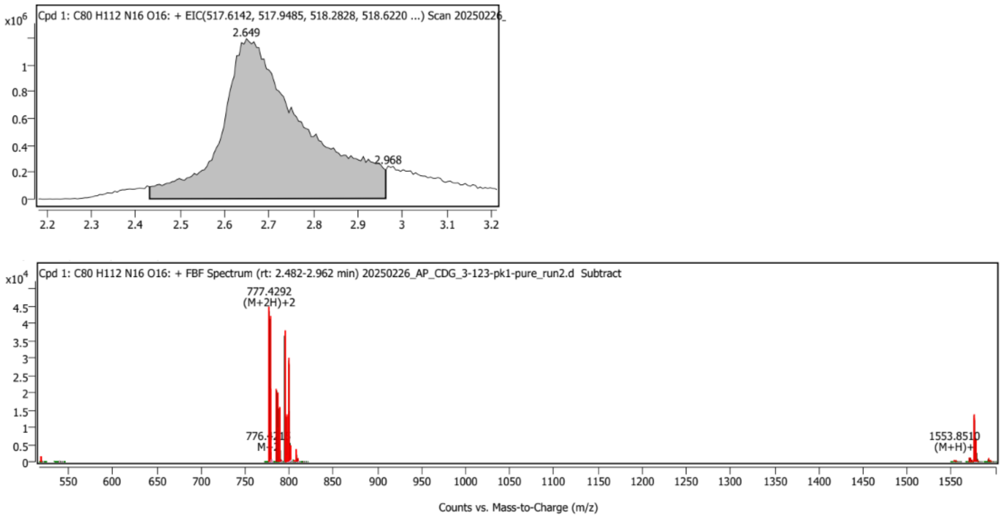


**Figure S51** EIC and HRMS for **CP[4,4]**; HRMS: calcd for $[M+H]^+$: $C_{80}H_{112}N_{16}O_{16}$, 1553.8515; found; $[M+H]^+$ 1553.8510.

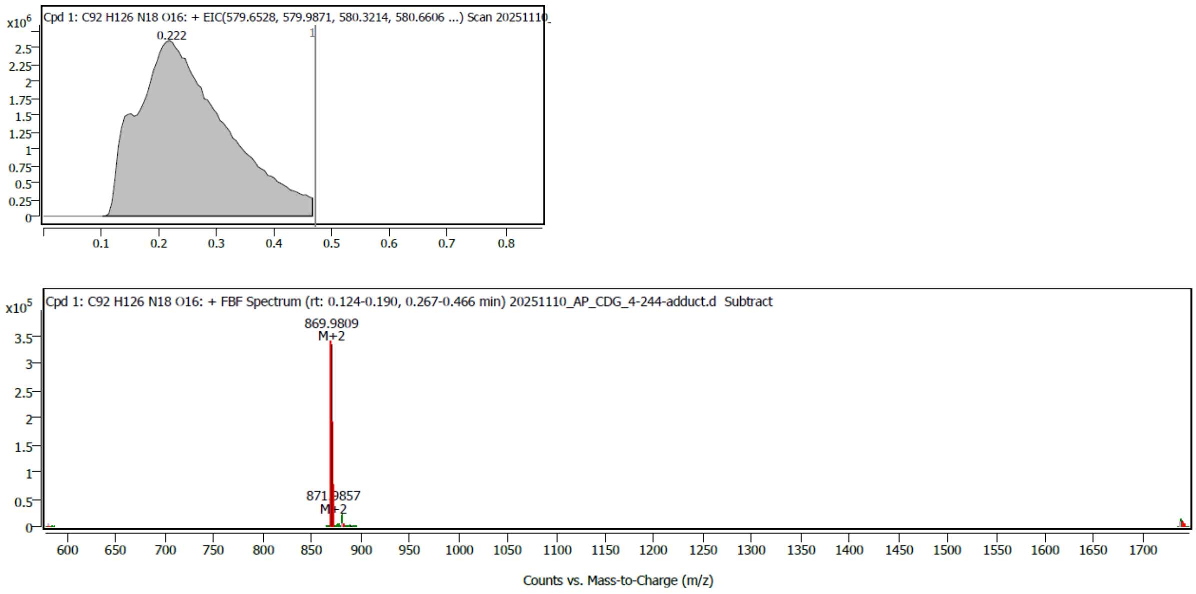


**Figure S52** EIC and HRMS of the **CP[4,4]⊂2$_{TFA}$** complex; HRMS: calcd for $[M+2H]^{2+}$: C92H126N18O16, 869.4794; found; $[M+2H]^{2+}$ 869.9809.

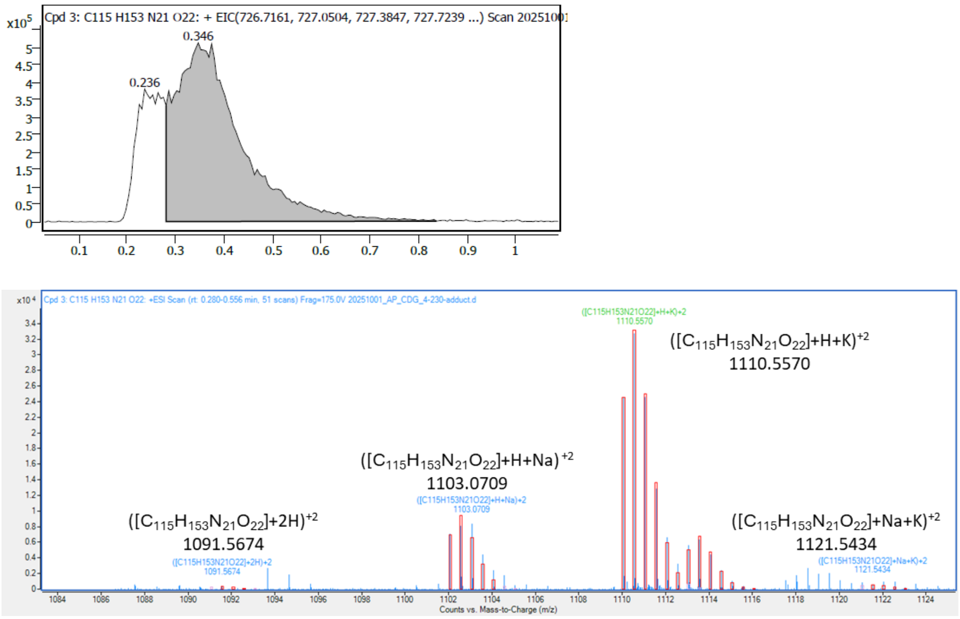


**Figure S53** EIC and HRMS of the **CP[4,4]⊂3** complex; HRMS: calcd for $[M+H+K]^{2+}$: C115H154N21O22K, 1110.5619; found; $[M+H+K]^{2+}$: 1110.5570.

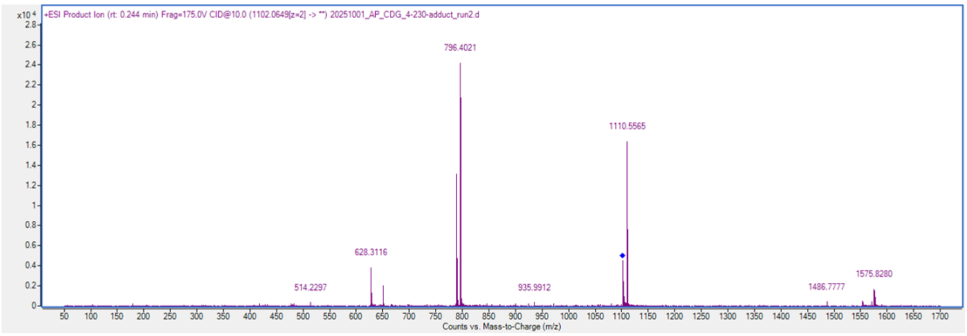


**Figure S54** MS/MS of the **CP[4,4]⊂3** complex was carried out using targeted precursor isolation at 1102.065 Da and a wide precursor isolation window of 9 Da at a retention time of 0.35min, at collision energies of 10, 20 and 40V with an MSMS range of 50-1700Da and a scan rate of 4 spectra/s. Source conditions were as previously stated, LC conditions incorporated a restriction capillary with an unbuffered isocratic Aqueous/MeOH mix at 1:1 ratio.

# 6. Docking Studies

All docking studies were performed using AutoDock Vina[1] to gain qualitative information about the geometry of the host-guest complexes. Analysis of **CP[4,4]** using MoloVol, gave a measurement of 97.6 Å for the accessible volume. [2]

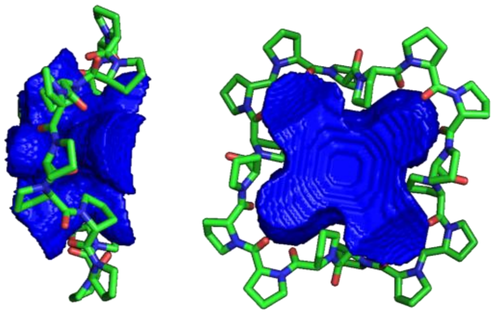

**Figure S55** Accessible volume of **CP[4,4]** calculated using MoloVol, side and front views.

### CP[4,4] Host Preparation:

The SC-XRD structure of all-junctions-*trans* **CP[4,4]** (CCDC 2534359) was used. The protein structure was prepared for docking using AutoDockTools v1.5.7 and UCSF ChimeraX.[1,3] All co-crystallized water molecules were removed. Polar hydrogens were added and Kollman partial charges were assigned to the macromolecule. The final receptor file was saved in PDBQT format.

**Table S2** Grid box parameters and AutoDock Vina settings used for molecular docking of **CP[4,4]**.

| Host | Grid Center (x, y, z) in Å | Grid Dimensions (x, y, z) in Å | Exhaustiveness | Number of Modes |
|---|---|---|---|---|
| All-junctions-*trans* **CP[4,4]** | X= 11.901, y= 11.901, z= 5.202 | X= 40, y= 40, z= 40 | 10 | 9 |

### Ligands Preparation:

**2:** The 2D structures of the synthesized compounds were drawn using ChemDraw. The 3D geometries were generated and energy-minimized using Avogadro and MOPAC employing the MMFF94 force field and SQM at the PM6 level. Gasteiger charges were assigned, and all rotatable bonds were defined using AutoDockTools v1.5.7. The prepared ligands were saved in PDBQT format.

**3:** The 3D structure for this ligand was retrieved by the Cambridge Crystallographic database (CCDC 2156434).[4] Gasteiger charges were assigned, and all rotatable bonds were defined using AutoDockTools v1.5.7. The prepared ligands were saved in PDBQT format.

**Docking output info and poses for CP[4,4]⊂$2_X$**

| CP[4,4]⊂$2_X$ Mode: | Binding Affinity (Kcal/mol) | Poses |
|---|---|---|
| 1 | -4.3 | 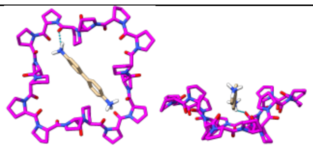 |
| 2 | -4.3 | 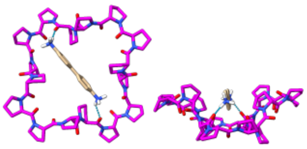 |
| 3 | -4.3 | 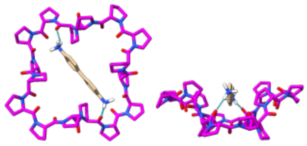 |
| 4 | -4.3 | 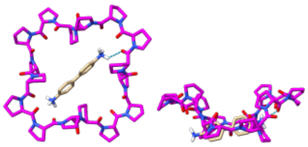 |
| 5 | -4.3 | 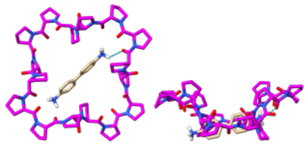 |
| 6 | -4.3 | 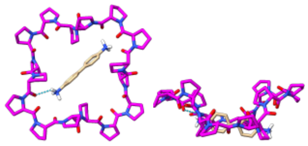 |
| 7 | -4.3 | 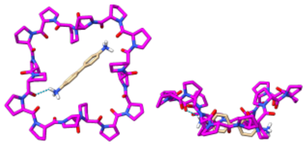 |
| 8 | -4.3 | 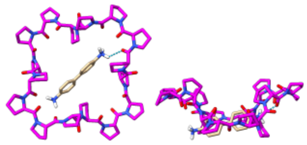 |
| 9 | -4.2 | 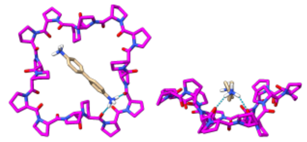 |

## Docking output info and poses for CP[4,4]⊂3

| CP[4,4]⊂3 Mode: | Binding Affinity (Kcal/mol) | Poses |
|---|---|---|
| 1 | -6.7 | 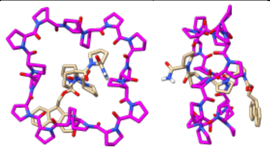 |
| 2 | -6.7 | 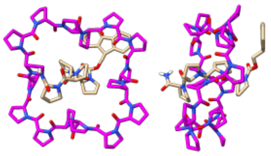 |
| 3 | -6.7 | 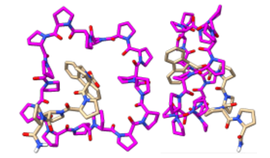 |
| 4 | -6.6 | 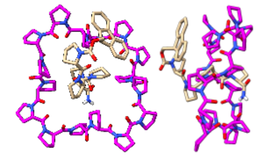 |
| 5 | -6.6 | 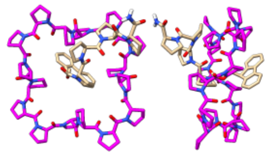 |
| 6 | -6.6 | 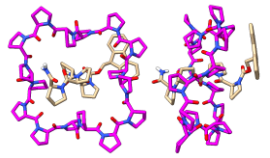 |
| 7 | -6.6 | 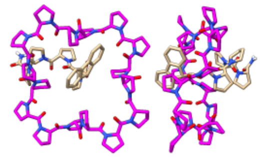 |
| 8 | -6.6 | 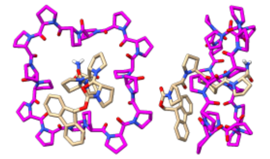 |
| 9 | -6.6 | 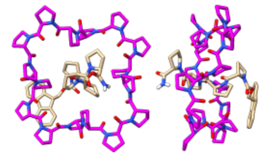 |

# 7. Binding Affinity Studies

The binding affinity between **CP[4,4]** and **2**$_{Cl}$ was determined by $^1$H-NMR titration where we monitored the perturbation of the **CP[4,4]** signal at 4.52 ppm as a function of the guest concentration (Figure 4d, 4.52 ppm → 4.40 ppm). The data was fitted using a 1:1 host:guest binding model using BindFit v0.5.[5–7] The **K error** obtained is <4% which is combined with a **SSR** (Sum of Square Residuals) of 4.372E-05. These data combined with the analysis of the Output Fit Quality suggests a very high-quality fit with both **Covariance** and **RMS** extremely closed to 0.

**Input Data:**

| Host concentration / M | Guest concentration / M | Proton |
|---|---|---|
| 0.002253703 | 0 | 4.5176 |
| 0.002253703 | 0.002214941 | 4.4887 |
| 0.002253703 | 0.004354981 | 4.4728 |
| 0.002253703 | 0.006423855 | 4.4601 |
| 0.002253703 | 0.008425056 | 4.4493 |
| 0.002253703 | 0.010361851 | 4.4399 |
| 0.002253703 | 0.012237299 | 4.4318 |
| 0.002253703 | 0.01405427 | 4.4241 |
| 0.002253703 | 0.015815456 | 4.4172 |
| 0.002253703 | 0.019470432 | 4.4055 |
| 0.002253703 | 0.023016536 | 4.3958 |

**Input Options:**

| | |
|---|---|
| **Fitter** | nmr1to1 |
| **K** | 100 |
| **Dilute** | FALSE |
| **Subtract initial values** | TRUE |
| **Method** | Nelder-Mead |
| **Flavour** | |

**Output Parameters:**

| K | K error (%) | SSR | Datapoints fitted | Params fitted | H coeffs | HG coeffs | Raw coeffs 1 | Raw coeffs 2 |
|---|---|---|---|---|---|---|---|---|
| 70.8235002 | 3.993679469 | 4.372E-05 | 11 | 2 | 4.5176 | 4.319734 | 4.5176 | 4.319733995 |

**Output Fit:**

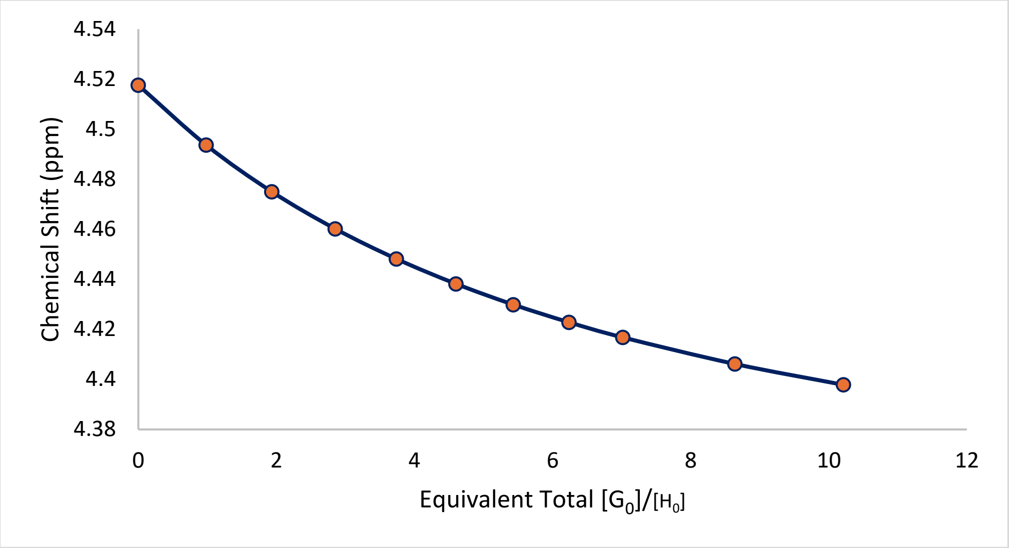

4.54
4.52
4.5
4.48
4.46
4.44
4.42
4.4
4.38
Chemical Shift (ppm)
0
2
4
6
8
10
12
Equivalent Total [G0]/[H0]


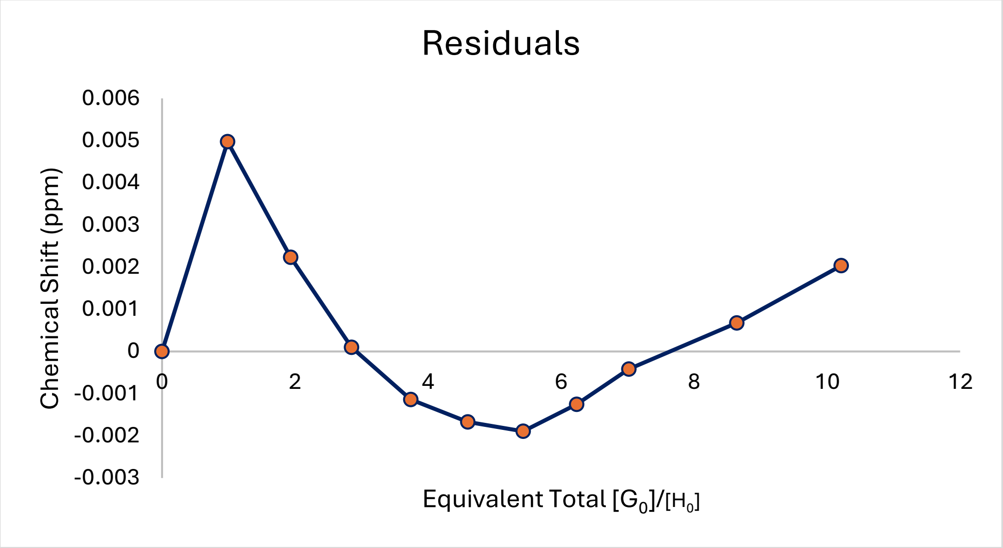

Residuals
0.006
0.005
0.004
0.003
0.002
0.001
0
-0.001
-0.002
-0.003
Chemical Shift (ppm)
0
2
4
6
8
10
12
Equivalent Total [G0]/[H0]


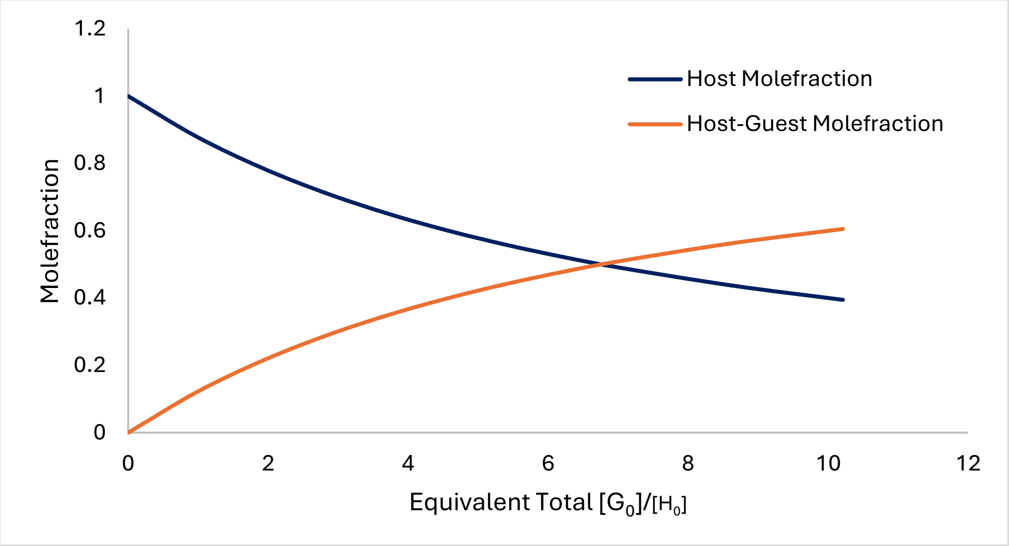

1.2
1
0.8
0.6
0.4
0.2
0
Molefraction
Host Molefraction
Host-Guest Molefraction
0
2
4
6
8
10
12
Equivalent Total [G0]/[H0]

**Output Fit Quality:**

| | |
|---|---|
| **RMS: Proton 1** | 0.001993622 |
| **RMS: Total** | 0.001993622 |
| **Covariance: Proton 1** | 0.00312958 |
| **Covariance: Total** | 0.00312958 |

# 8. Single Crystal X-Ray Diffraction Data

**SC-XRD of all-junctions-*trans* CP[4,4] (CCDC 2534359):** Diffraction data of a translucent, light-colourless block-shaped crystal were measured at 108.2 (8) K using a SuperNova Dual Cu four-circle diffractometer (Atlas detector) equipped with a micro-focus sealed-tube X-ray source Cu Kα radiation (λ = 1.54184 Å) and a mirror monochromator. Data integration was performed with CrysAlisPro and a Gaussian absorption correction using SCALE3 ABSPACK was applied. The structure was solved by dual methods using SHELXT and refined by full-matrix least-squares methods against $F^2$ with SHELXL in Olex2. All non-hydrogen atoms were refined with anisotropic displacement parameters. All C-bound hydrogen atoms were placed in calculated positions and refined using a riding model, with $U_{iso}$ values set at 1.5 × $U_{eq}$ for terminal sp³ carbon atoms and 1.2 × $U_{eq}$ for all other carbon atoms.

The title compound crystallizes in the space group $I\bar{4}$ with unit-cell parameters a = 23.802(2) Å, b = 23.802(3) Å, c = 10.4038(10) Å, V = 5894.1(11) Å³. The asymmetric unit contains several water molecules, some of which are positionally disordered; for these disordered molecules, hydrogen atoms could not be located and were not included in the refinement.

A probe-based solvent-accessible volume calculation (probe radius = 1.20 Å) indicates that the framework contains interconnected channels with a total accessible volume of 5139.3 Å³ (87.19% of the unit cell). The largest included sphere within these channels has an estimated diameter of 22.83 Å. The ordered water molecules are located within these cavities and interact with the framework via O—H···O hydrogen bonds, while the disordered sites occupy diffuse regions of the channels.

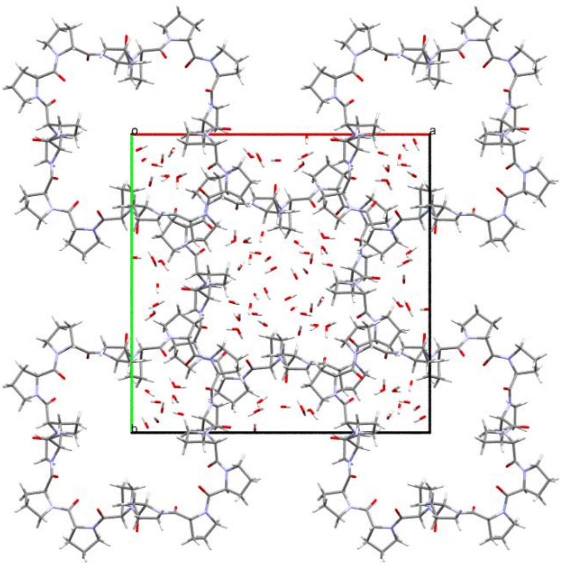

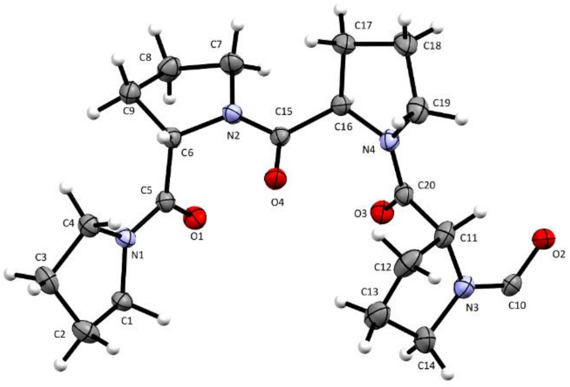


<u>Crystal data and structure refinement all-junctions-*trans* **CP[4,4]**</u>

| | |
|---|---|
| Empirical formula | $C_{80}H_{112}N_{16}O_{16}$ |
| Formula weight | 1553.85 |
| Temperature [K] | 108.2(8) |
| Crystal system | tetragonal |
| Space group (number) | $I\bar{4}$ (82) |
| *a* [Å] | 23.802(2) |

| | |
|---|---|
| *b* [Å] | 23.802(3) |
| *c* [Å] | 10.4038(10) |
| α [°] | 90 |
| β [°] | 90 |
| γ [°] | 90 |
| Volume [Å$^3$] | 5894.1(11) |
| *Z* | 2 |
| $\rho_{calc}$ [gcm$^{-3}$] | 0.876 |
| $\mu$ [mm$^{-1}$] | 0.506 |
| *F*(000) | 1664 |
| Crystal size [mm$^3$] | 0.49×0.25×0.19 |
| Crystal colour | translucent light colorless |
| Crystal shape | block |
| Radiation | Cu $K_\alpha$ (λ=1.54184 Å) |
| 2θ range [°] | 9.28 to 153.72 (0.79 Å) |
| Index ranges | −30 ≤ h ≤ 29<br>−30 ≤ k ≤ 29<br>−13 ≤ l ≤ 9 |
| Reflections collected | 23282 |
| Independent reflections | 5967<br>$R_{int}$ = 0.0310<br>$R_{sigma}$ = 0.0262 |
| Completeness to<br>θ = 67.684° | 99.4 % |
| Data / Restraints / Parameters | 5967/0/253 |
| Absorption correction<br>$T_{min}/T_{max}$ (method) | 0.507/1.000<br>(gaussian) |
| Goodness-of-fit on $F^2$ | 1.045 |
| Final *R* indexes<br>[$I \geq 2\sigma(I)$] | $R_1$ = 0.0476<br>w$R_2$ = 0.1387 |
| Final *R* indexes<br>[all data] | $R_1$ = 0.0517<br>w$R_2$ = 0.1427 |
| Largest peak/hole [eÅ$^{-3}$] | 0.27/−0.27 |
| Flack X parameter | 0.43(5) |

**SC-XRD of all-junctions-*cis* CP[4,4] (CCDC 2532549):** A translucent light colourless crystal of compound CP[4,4] in crystallized in the tetragonal space group $I\bar{4}$ from a solution in $CDCl_3$. Diffraction data were collected on a Rigaku Oxford Diffraction Synergy-S diffractometer using a microfocus copper X-radiation source Kα radiation (λ = 1.54184 Å) from multilayer focussing optics. The sample was maintained at 150.0 (2) K throughout, using an Oxford Cryosystems Cryostream 800, to increase higher angle diffraction resolution and minimize sample damage. The diffraction data collection processing and corrections have been completed using the CrysAlisPro software suite applying a Gaussian absorption correction using SCALE3

ABSPACK based on the full crystal shape generated from sample face indexing. The structure was solved by dual space methods using SHELXT and refined by full-matrix least-squares methods against $F^2$ with SHELXL within the Olex2 graphical interface. All non-hydrogen atoms were refined with anisotropic displacement parameters. All C-bound hydrogen atoms were placed in calculated positions and refined using a riding model, with $U_{iso}$ values set at 1.5 × $U_{eq}$ for terminal $sp^3$ carbon atoms and 1.2 × $U_{eq}$ for all other carbon atoms. The refined model has been used to unambiguously determine the full structure of the compound, although the chloroform present in the crystals occupies locations that only have weak interactions and therefore display significant disorder in the final structural model.

Crystal data and structure refinement all-junctions-*cis* **CP[4,4]**

| | |
|---|---|
| Identification code | half_frames_ani_1 |
| Empirical formula | $C_{93}H_{125}Cl_{39}N_{16}O_{16}$ |
| Formula weight | 3105.63 |
| Temperature/K | 150.00(10) |
| Crystal system | tetragonal |
| Space group | $I\bar{4}$ |
| a/Å | 26.7054(3) |
| b/Å | 26.7054(3) |
| c/Å | 9.55515(10) |
| α/° | 90 |
| β/° | 90 |
| γ/° | 90 |
| Volume/Å$^3$ | 6814.52(16) |
| Z | 2 |
| $\rho_{calc}$g/cm$^3$ | 1.514 |
| μ/mm$^{-1}$ | 7.614 |
| F(000) | 3172.0 |
| Crystal size/mm$^3$ | 0.245 × 0.066 × 0.045 |
| Radiation | Cu Kα (λ = 1.54184) |
| 2Θ range for data collection/° | 4.68 to 154.854 |
| Index ranges | -24 ≤ h ≤ 29, -28 ≤ k ≤ 31, -11 ≤ l ≤ 12 |
| Reflections collected | 19964 |

| | |
|---|---|
| Independent reflections | 5956 [$R_{int}$ = 0.0710, $R_{sigma}$ = 0.0386] |
| Data/restraints/parameters | 5956/377/396 |
| Goodness-of-fit on $F^2$ | 1.069 |
| Final R indexes [I>=2σ (I)] | $R_1$ = 0.0905, $wR_2$ = 0.2532 |
| Final R indexes [all data] | $R_1$ = 0.0958, $wR_2$ = 0.2585 |
| Largest diff. peak/hole / e $Å^{-3}$ | 1.19/-0.63 |
| Flack parameter | 0.017(14) |

# 9. HPLC Studies – Isomers Equilibration

The two **CP[4,4]** isomers (confirmed by LCMS) were purified on a C18 (SiliaChrom® Plus HPLC Column, 10 x 250 mm, 5 µm, 300 Å) at 1.9 mL/min flow rate monitored at 225/240/254 nm wavelengths. The method used for this separation was as follows: the mobile phases of 0.1% formic acid in water *vs* methanol on a gradient from 50% aqueous to 60% methanol over 12min, with an isocratic hold of 3min at 60% methanol and an isocratic hold at 100% methanol for 3min. Reinjection of the individually collected fractions at 10.320 min and13.637 min ($\Delta t$ = 198 s), handled without delay and using the same method, yielded chromatograms in which both isomers reappeared, consistent with rapid equilibration of the two isomers in aqueous media.

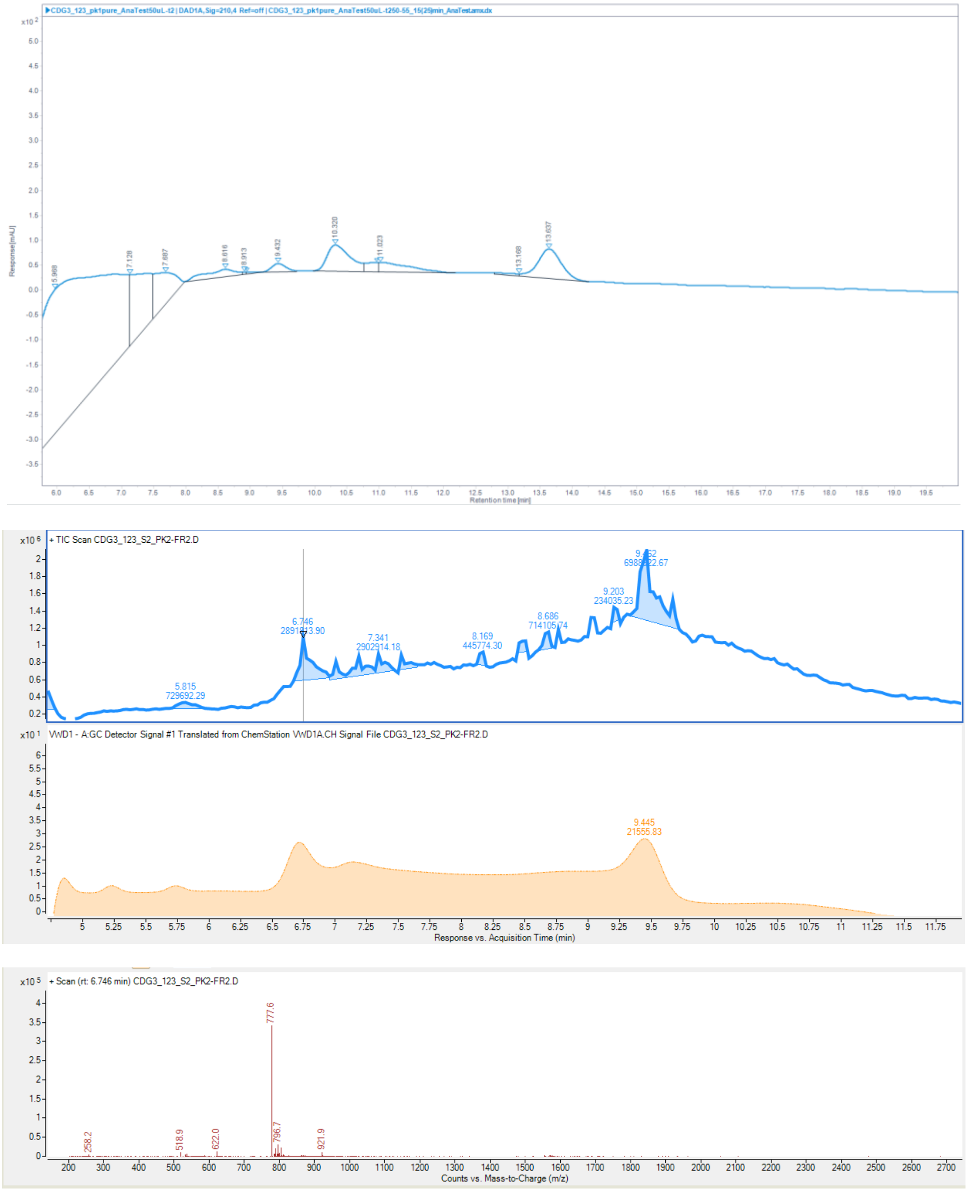


**Figure S56** Top panel: UV chromatogram (225 nm) of peak 1 pure fraction (r.t. = 10. 320 min) showing the two peaks of **CP[4,4]** isomers in equilibrium; bottom panel: TIC chromatogram, UV chromatogram (225 nm) and mass fragmentation pattern of pure peak 1 having the same mass as peak 2 (Figure S45).

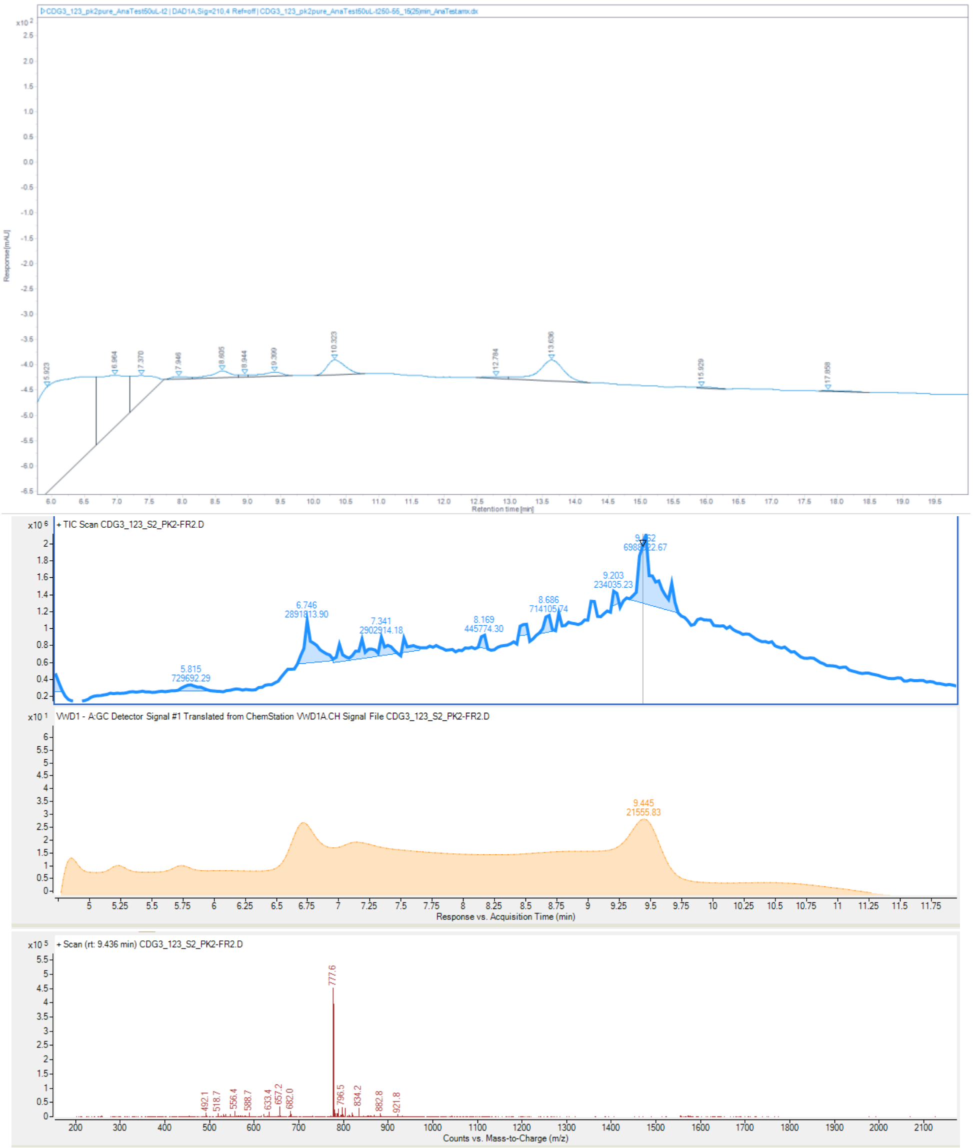


**Figure S57** Top panel: UV chromatogram (225 nm) of peak 2 pure fraction (r.t. = 13. 637 min) showing the two peaks of **CP[4,4]** isomers in equilibrium; bottom panel: TIC chromatogram, UV chromatogram (225 nm) and mass fragmentation pattern of pure peak 2 having the same mass as peak 1 (Figure S44).

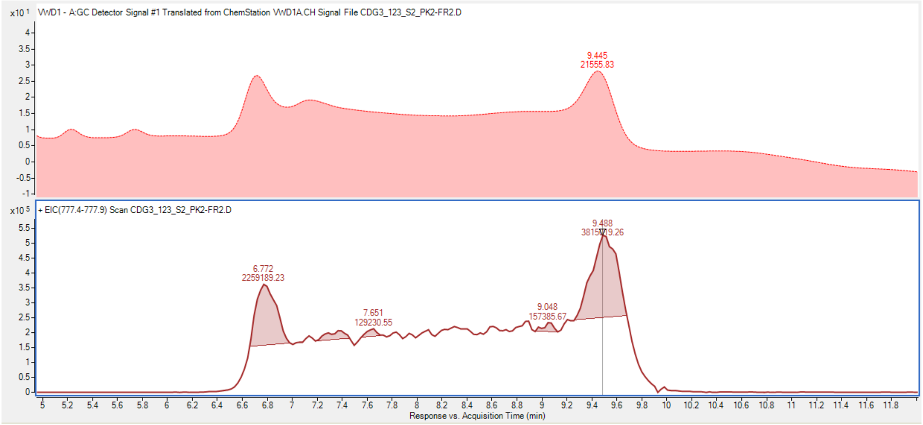


**Figure S58** Extracted Ion count Chromatogram (EIC) for the *m/z* 777.4 - 777.9 mass range ([**CP[4,4]**+2H]$^{2+}$), showing the two major isomer peaks at 6.772 min and 9.488 min. The broad region between the two major peaks shows a low ion count for the target mass, which is likely due to other isomers present in the aqueous-organic solvent mixture (56–58% MeOH).

# 10. Molecular Dynamics and DFT Calculations

The initial structure of the proline crow was obtained from a PDB file (add the source of the crystal). System preparation was carried out using the tleap module of the AMBERTools1 software package. The system was parameterized using the ff14SB2 force field and solvated in an octahedral periodic box of explicit TIP3P 3 water molecules, with a minimum buffer distance of 10 Å between the solute and the box boundaries. The Molecular Dynamics (MD) simulations were performed using the Amber 244,5 software suite under periodic boundary conditions. A cut-off distance of 10 Å was applied to short-range non-bonded interactions, while long-range electrostatic interactions were treated using the Particle Mesh Ewald (PME)6 method. Covalent bonds involving hydrogen atoms were constrained using the SHAKE7 algorithm, allowing a time step of 2 fs. Energy minimization was conducted in two stages. In the first stage, solvent molecules were minimized while the proline crow was restrained using a harmonic potential with a force constant of 10.0 kcal·mol$^{-1}$·Å$^{-2}$. This step comprised of 5,000 minimization cycles, including 2,500 steps of steepest descent followed by 2,500 steps of conjugate gradient minimization, performed under constant volume conditions. In the second stage, all restraints were removed and the entire system was subjected to an additional 5,000 cycles of energy minimization using the same minimization scheme.

Following minimization, the system was equilibrated by means a 20 ps MD simulation under constant volume conditions. The simulation was initiated with zero initial velocities, and the temperature was gradually increased to 300 K using Langevin 8 dynamics with a collision frequency of 1.0 ps$^{-1}$. During this phase, weak positional restraints (10.0 kcal·mol$^{-1}$·Å$^{-2}$) were applied to the solute to prevent large structural deviations while allowing solvent relaxation. Trajectory coordinates were saved every 50 steps, and energy information was written every 100 steps. The equilibrated system was subsequently subjected to a 100 ns production MD simulation under NPT conditions at 300 K and 1 atm. Temperature was maintained using Langevin dynamics with a collision frequency of 1.0 ps$^{-1}$, and pressure was regulated using isotropic coupling with relaxation time of 2.0 ps. No positional restraints were applied during the production run. The simulation comprised 50,000,000 integration steps with a time step of 2 fs. Trajectory coordinates and energetic information were written every 500 steps (1 ps), and restart files were generated at regular intervals. Trajectory analysis was performed primarily using CPPTRAJ9. Molecular dynamics simulations were performed to investigate the interaction of the proline crow with explicit water molecules. The trans conformation was maintained throughout the 100 ns simulation, as confirmed by the RMSD profile (Figure SI1). A recurring structural feature observed during the simulation is the presence of a single water molecule bridging two intercalated proline fragments via hydrogen bonding (Figure 1). This water-mediated interaction is associated with an oxygen–oxygen distance of approximately 4 Å between the two proline residues involved (Figure 2). The formation and stability of this motif are strongly influenced by key dihedral angles that define the pinch-like architecture of the proline crow. Alterations in these dihedral angles disrupt the characteristic ~4 Å oxygen–oxygen separation, thereby preventing the formation of the bridging hydrogen-bonded interaction (Figure 2). Analysis of the simulation trajectories shows that the ~4 Å oxygen–oxygen distance predominates in the majority of frames (Figure SI2). In addition, a systematic decrease in the relevant dihedral angles relative to the initial structure is observed and maintained throughout the simulation (Figure SI3). This conformational adjustment promotes a geometry that is more favourable for the formation and persistence of the water-mediated hydrogen-bonded motif.

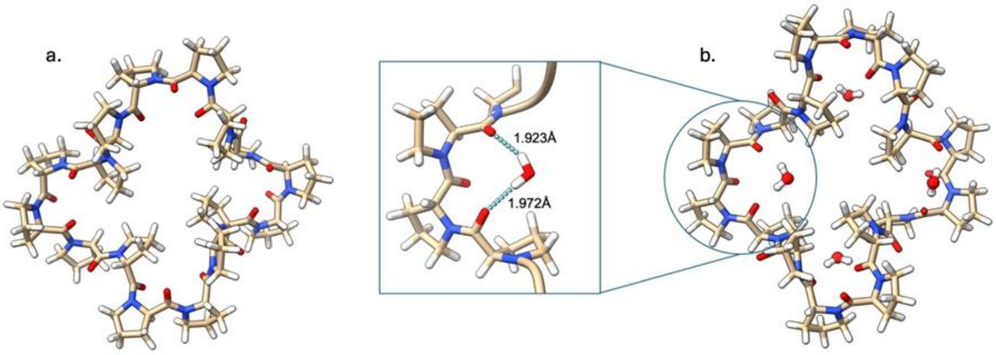


**Figure S59** (a) Initial structure of the proline crow comprising 16 proline fragments. (b) Structure of the proline crow showing four water molecules hydrogen-bonded to the system.

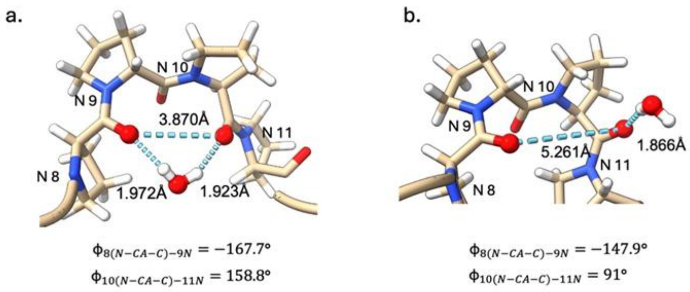


**Figure S60** Bond lengths (Å) and dihedral angles characterizing the (a) hydrogen-bonded motif and the (d) disrupted structure.

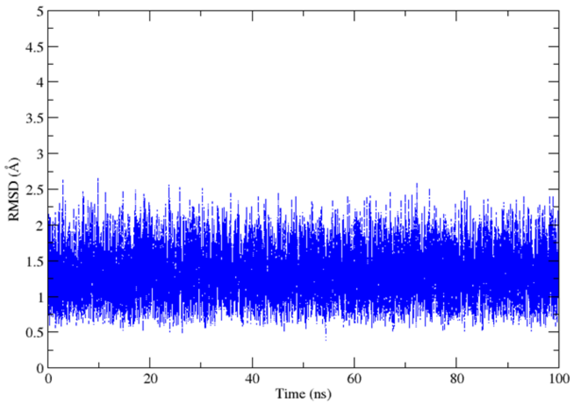


**Figure S61** Time series data for the RMSD with respect to the first simulation frame.

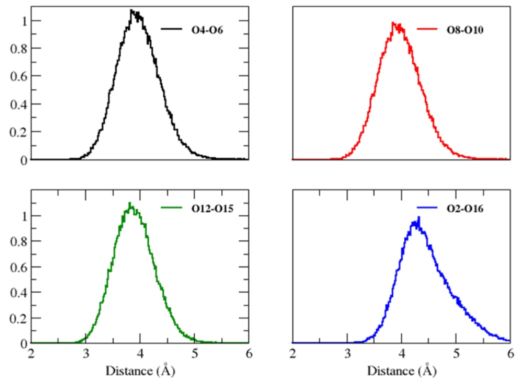


**Figure S62** Histogram showing the distribution of oxygen–oxygen distances sampled during the 100 ns molecular dynamics simulation.

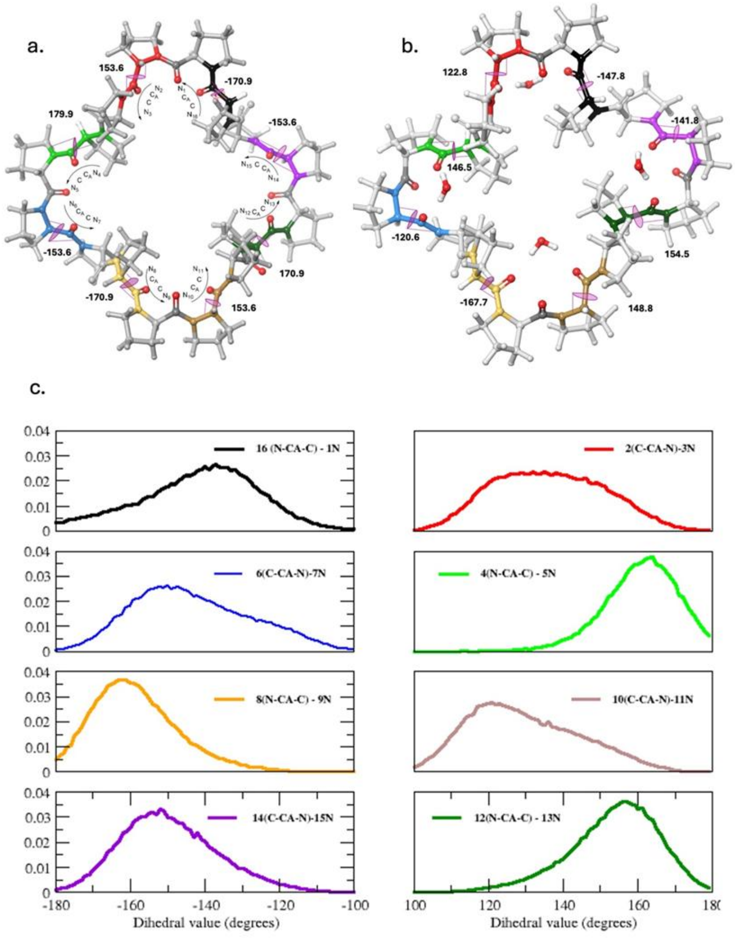


**Figure S63** Dihedral angle analysis. (a) Dihedral angles of the initial structure. (b) Dihedral angles of the structure hydrogen-bonded to four water molecules. (c) Distribution of dihedral angles over the 100 ns molecular dynamics simulation. The color scheme corresponds to the structures shown in panels (a) and (b).

# 11. References


[1] O. Trott, A. J. Olson, "AutoDock Vina: improving the speed and accuracy of docking with a new scoring function, efficient optimization and multithreading" *J. Comput. Chem.* **2010**, *31*, 455.

[2] J. B. Maglic, R. Lavendomme, "MoloVol: an easy-to-use program for analyzing cavities, volumes and surface areas of chemical structures" *J Appl Crystallogr. 1600-5767* **2022**, *55*, 1033–1044.

[3] E. C. Meng, T. D. Goddard, E. F. Pettersen, G. S. Couch, Z. J. Pearson, J. H. Morris, T. E. Ferrin, "UCSF ChimeraX: Tools for structure building and analysis" *Protein Sci.* **2023**, *32*, DOI 10.1002/pro.4792.

[4] D. F. Brightwell, G. Truccolo, K. Samanta, E. J. Fenn, S. J. Holder, H. J. Shepherd, C. S. Hawes, A. Palma, "A Reversibly Porous Supramolecular Peptide Framework" *Chem. - A Eur. J.* **2022**, *28*, e202202368.

[5], "Supramolecular chemistry tools | supramolecular.org," can be found under https://supramolecular.org/(accessed 9 March 2026), **n.d.**

[6] D. Brynn Hibbert, P. Thordarson, "The death of the Job plot, transparency, open science and online tools, uncertainty estimation methods and other developments in supramolecular chemistry data analysis" *Chem. Commun.* **2016**, *52*, 12792–12805.

[7] P. Thordarson, "Determining association constants from titration experiments in supramolecular chemistry" *Chem. Soc. Rev.* **2011**, *40*, 1305–1323.
