# Supplementary material for: Cyclo‐Polyproline: Chameleonic All‐Peptide Macrocycles With Induced‐Fit Host‐Guest Recognition
Source: Angew Chem Int Ed Engl. 2026 May 14;65(27):e8698780. doi: 10.1002/anie.8698780 (PMC13327576; doi:10.1002/anie.8698780)

# checkCIF/PLATON report

Structure factors have been supplied for datablock(s) half_frames_ani_1

THIS REPORT IS FOR GUIDANCE ONLY. IF USED AS PART OF A REVIEW PROCEDURE FOR PUBLICATION, IT SHOULD NOT REPLACE THE EXPERTISE OF AN EXPERIENCED CRYSTALLOGRAPHIC REFEREE.

No syntax errors found.     CIF dictionary     Interpreting this report

# Datablock: half_frames_ani_1

---

| | | | |
|---|---|---|---|
| Bond precision: | C-C = 0.0171 A | | Wavelength=1.54184 |
| Cell: | a=26.7054(3) | b=26.7054(3) | c=9.55515(10) |
| | alpha=90 | beta=90 | gamma=90 |
| Temperature: | 150 K | | |

| | Calculated | Reported |
|---|---|---|
| Volume | 6814.53(17) | 6814.52(16) |
| Space group | I -4 | I -4 |
| Hall group | I -4 | I -4 |
| Moiety formula | C80 H112 N16 O16, 13(C H Cl3) | 13(C H Cl3), C80 H112 N16 O16 |
| Sum formula | C93 H125 Cl39 N16 O16 | C93 H125 Cl39 N16 O16 |
| Mr | 3105.64 | 3105.63 |
| Dx,g cm-3 | 1.514 | 1.514 |
| Z | 2 | 2 |
| Mu (mm-1) | 7.614 | 7.614 |
| F000 | 3172.0 | 3172.0 |
| F000’ | 3206.53 | |
| h,k,lmax | 33,33,12 | 29,31,12 |
| Nref | 7241[ 3847] | 5956 |
| Tmin,Tmax | 0.575,0.710 | 0.231,0.916 |
| Tmin’ | 0.147 | |

Correction method= # Reported T Limits: Tmin=0.231 Tmax=0.916
AbsCorr = GAUSSIAN

Data completeness= 1.55/0.82 Theta(max)= 77.427

R(reflections)= 0.0905( 5337) wR2(reflections)= 0.2585( 5956)

S = 1.069 Npar= 396

---

The following ALERTS were generated. Each ALERT has the format
**test-name_ALERT_alert-type_alert-level.**
Click on the hyperlinks for more details of the test.

---

### Alert level B

```
PLAT340_ALERT_3_B Low Bond Precision on  C-C Bonds ..............    0.01709 Ang.
```

---

### Alert level C

```
PLAT029_ALERT_3_C _diffrn_measured_fraction_theta_full value Low .      0.968 Why?
PLAT042_ALERT_1_C Calc. and Reported MoietyFormula Strings  Differ     Please Check
              Calc: C80 H112 N16 O16, 13(C H Cl3)
              Rep.: 13(C H Cl3), C80 H112 N16 O16
PLAT084_ALERT_3_C High wR2 Value (i.e. > 0.25) ..................       0.26 Report
PLAT241_ALERT_2_C High   'MainMol' Ueq as Compared to Neighbors of       C19 Check
PLAT244_ALERT_4_C Low    'Solvent' Ueq as Compared to Neighbors of       C3S Check
PLAT250_ALERT_2_C Large U3/U1 Ratio for <U(i,j)> Tensor(Resd    2)       2.2 Note
PLAT260_ALERT_2_C Large Average Ueq of Residue Including      Cl4S      0.135 Check
PLAT336_ALERT_2_C Long Bond Distance for ..... C3S      -Cl7S           1.851 Ang.
PLAT790_ALERT_4_C Centre of Gravity not Within Unit-Cell: Resd.  #          1 Note
              C80 H112 N16 O16
PLAT906_ALERT_3_C Large K Value in the Analysis of Variance ......      2.106 Check
PLAT911_ALERT_3_C Missing FCF Refl Between Thmin & STh/L=    0.600        105 Report
               12 22  0,  -7 27  0, -10 28  0,  -8 28  0,  -6 28  0,  -4 28  0,
               -2 28  0,   0 28  0,   2 28  0,   4 28  0,   6 28  0, -13 29  0,
              -11 29  0,  -9 29  0,  -7 29  0,  -5 29  0,  -3 29  0,  -1 29  0,
                1 29  0,   3 29  0,   5 29  0,   7 29  0,   9 29  0,  11 29  0,
               13 29  0, -10 30  0,  -8 30  0,  -6 30  0,  -4 30  0,  -2 30  0,
                   (   75 More Missing: see the .ckf listing file)
```

---

### Alert level G

```
PLAT002_ALERT_2_G Number of Distance or Angle Restraints on AtSite         31 Note
PLAT072_ALERT_2_G SHELXL First  Parameter in WGHT  Unusually Large       0.14 Report
PLAT083_ALERT_2_G SHELXL Second Parameter in WGHT  Unusually Large      27.55 Why ?
PLAT171_ALERT_4_G The CIF-Embedded .res File Contains EADP Records          9 Report
PLAT176_ALERT_4_G The CIF-Embedded .res File Contains SADI Records         24 Report
PLAT187_ALERT_4_G The CIF-Embedded .res File Contains RIGU Records          1 Report
PLAT191_ALERT_3_G A Non-default SADI Restraint Value has been used     0.0400 Report
PLAT191_ALERT_3_G A Non-default SADI Restraint Value has been used     0.0400 Report
PLAT191_ALERT_3_G A Non-default SADI Restraint Value has been used     0.0400 Report
PLAT191_ALERT_3_G A Non-default SADI Restraint Value has been used     0.0400 Report
PLAT191_ALERT_3_G A Non-default SADI Restraint Value has been used     0.0400 Report
PLAT191_ALERT_3_G A Non-default SADI Restraint Value has been used     0.0400 Report
PLAT191_ALERT_3_G A Non-default SADI Restraint Value has been used     0.0400 Report
PLAT191_ALERT_3_G A Non-default SADI Restraint Value has been used     0.0400 Report
PLAT299_ALERT_4_G Atom Site Occupancy Constrained at ............         0.5 Check
```

```
              C14     C14A    H13A    H13B    H13C    H13D    H14A    H14B
              H14C    H14D    H15A    H15B    H15C    H15D    Cl7S    Cl8S
              Cl9S    Cl10    Cl11    Cl12    H3SA    H3S
PLAT300_ALERT_4_G Atom Site Occupancy of Cl4S       Constrained at       0.25 Check
PLAT300_ALERT_4_G Atom Site Occupancy of Cl5S       Constrained at       0.25 Check
PLAT300_ALERT_4_G Atom Site Occupancy of Cl6S       Constrained at       0.25 Check
PLAT300_ALERT_4_G Atom Site Occupancy of C2S        Constrained at       0.25 Check
PLAT300_ALERT_4_G Atom Site Occupancy of H2S        Constrained at       0.25 Check
PLAT301_ALERT_3_G Main Residue  Disorder .............(Resd    1)         11% Note
PLAT302_ALERT_4_G Anion/Solvent/Minor-Residue Disorder (Resd    2)         75% Note
PLAT302_ALERT_4_G Anion/Solvent/Minor-Residue Disorder (Resd    4)        100% Note
PLAT302_ALERT_4_G Anion/Solvent/Minor-Residue Disorder (Resd    5)        100% Note
PLAT302_ALERT_4_G Anion/Solvent/Minor-Residue Disorder (Resd    6)        100% Note
PLAT304_ALERT_4_G Non-Integer Number of Atoms in ..... (Resd    4)       4.18 Check
PLAT304_ALERT_4_G Non-Integer Number of Atoms in ..... (Resd    5)       1.25 Check
PLAT304_ALERT_4_G Non-Integer Number of Atoms in ..... (Resd    6)       0.81 Check
PLAT410_ALERT_2_G Short Intra H...H Contact  H7AB     ..H5       .       2.03 Ang.
                                                    x,y,z  =     1_555 Check
PLAT431_ALERT_2_G Short Inter HL..A Contact  Cl1S     ..O3       .       3.08 Ang.
                                                  x,y,1+z  =     1_556 Check
PLAT432_ALERT_2_G Short Inter X...Y Contact  Cl9S     ..C20      .       3.22 Ang.
                                                  x,y,1+z  =     1_556 Check
PLAT432_ALERT_2_G Short Inter X...Y Contact  O2       ..C1S      .       3.01 Ang.
                                                    x,y,z  =     1_555 Check
PLAT432_ALERT_2_G Short Inter X...Y Contact  O4       ..C4S      .       2.72 Ang.
                                                    x,y,z  =     1_555 Check
PLAT434_ALERT_2_G Short Inter HL..HL Contact Cl1S     ..Cl11     .       3.30 Ang.
                                                y,-1-x,-z  =     3_545 Check
PLAT434_ALERT_2_G Short Inter HL..HL Contact Cl2S     ..Cl5S     .       3.36 Ang.
                                   1/2+y,-3/2-x,1/2-z  =     7_535 Check
PLAT720_ALERT_4_G Number of Unusual/Non-Standard Labels .........          9 Note
              H3SA    H7AA    H7AB    H7BC    H7BD    H8AA    H8AB    H9AA
              H9AB
PLAT789_ALERT_4_G Atoms with Negative _atom_site_disorder_group  #          5 Check
PLAT790_ALERT_4_G Centre of Gravity not Within Unit-Cell: Resd.  #          2 Note
              C H Cl3
PLAT790_ALERT_4_G Centre of Gravity not Within Unit-Cell: Resd.  #          3 Note
              C H Cl3
PLAT790_ALERT_4_G Centre of Gravity not Within Unit-Cell: Resd.  #          4 Note
              C H Cl3
PLAT790_ALERT_4_G Centre of Gravity not Within Unit-Cell: Resd.  #          5 Note
              C H Cl3
PLAT790_ALERT_4_G Centre of Gravity not Within Unit-Cell: Resd.  #          6 Note
              C H Cl3
PLAT792_ALERT_1_G Model has Chirality at C5          (Polar  SpGr)          R Verify
PLAT792_ALERT_1_G Model has Chirality at C10         (Polar  SpGr)          R Verify
PLAT792_ALERT_1_G Model has Chirality at C12         (Polar  SpGr)          R Verify
PLAT792_ALERT_1_G Model has Chirality at C17         (Polar  SpGr)          S Verify
PLAT811_ALERT_5_G No ADDSYM Analysis: Too Many Excluded Atoms ....          ! Info
PLAT822_ALERT_4_G CIF-embedded .res Contains Negative PART Numbers          2 Check
PLAT860_ALERT_3_G Number of Least-Squares Restraints ............         377 Note
PLAT912_ALERT_4_G Missing # of FCF Reflections Above STh/L=  0.600        336 Note
PLAT915_ALERT_3_G No Flack x Check Done: Low Friedel Pair Coverage         77 %
PLAT950_ALERT_5_G Calculated (ThMax) and CIF-Reported Hmax Differ           4 Units
PLAT951_ALERT_5_G Calculated (ThMax) and CIF-Reported Kmax Differ           2 Units
PLAT969_ALERT_5_G The 'Henn et al.' R-Factor-gap value ..........      7.674 Note
              Predicted wR2: Based on SigI**2  3.37 or SHELX Weight 24.17
```

```
PLAT978_ALERT_2_G Number C-C Bonds with Positive Residual Density.         2 Info
```

---

```
   0 ALERT level A = Most likely a serious problem - resolve or explain
   1 ALERT level B = A potentially serious problem, consider carefully
  11 ALERT level C = Check. Ensure it is not caused by an omission or oversight
  55 ALERT level G = General information/check it is not something unexpected

   5 ALERT type 1 CIF construction/syntax error, inconsistent or missing data
  15 ALERT type 2 Indicator that the structure model may be wrong or deficient
  16 ALERT type 3 Indicator that the structure quality may be low
  27 ALERT type 4 Improvement, methodology, query or suggestion
   4 ALERT type 5 Informative message, check
```

---

It is advisable to attempt to resolve as many as possible of the alerts in all categories. Often the minor alerts point to easily fixed oversights, errors and omissions in your CIF or refinement strategy, so attention to these fine details can be worthwhile. It is up to the individual to critically assess their own results and, if necessary, seek expert advice.

---

**PLATON version of 26/09/2025; check.def file version of 20/09/2025**

**Datablock half_frames_ani_1** - ellipsoid plot

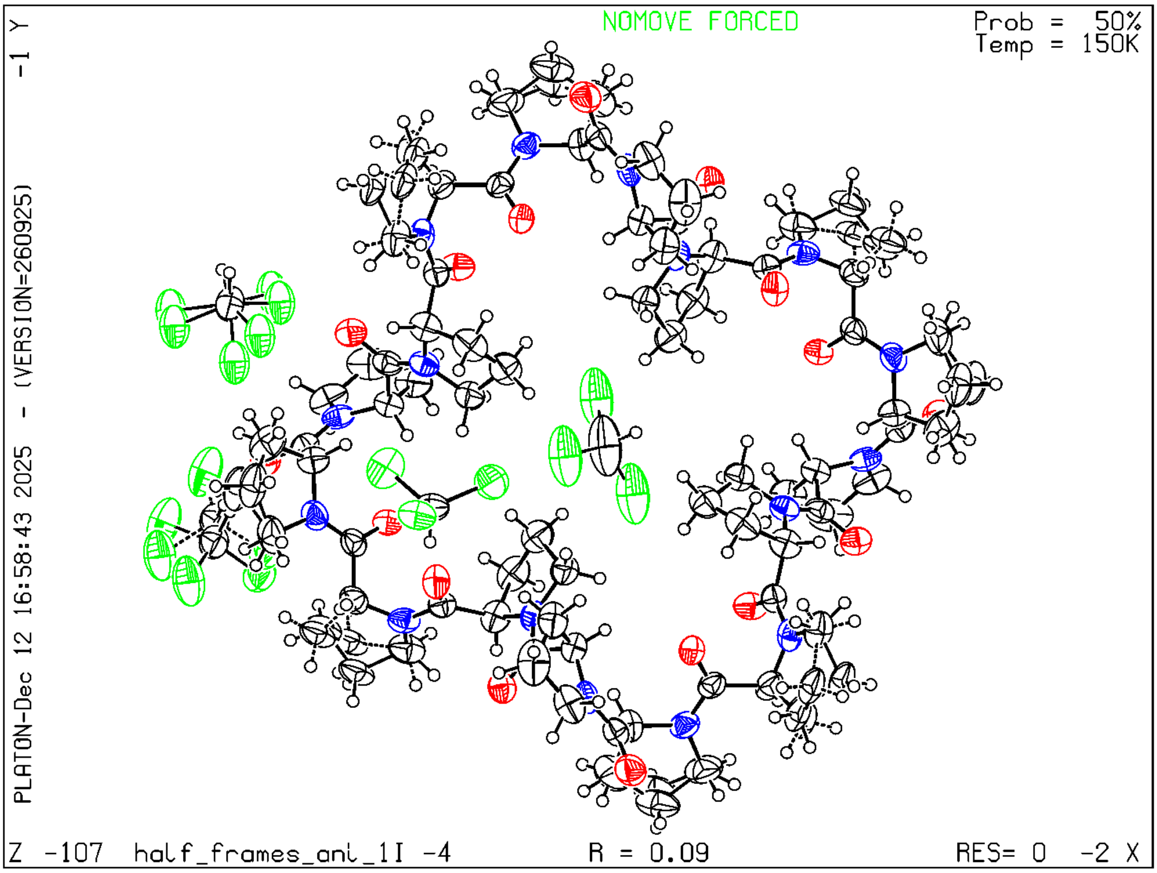

Supplement: Supplementary file 2 — Supporting File 2: anie72664‐sup‐0002‐Data.zip. [file ANIE-65-e8698780-s001.zip › anie72664-sup-0002-Data/checkcif-all-cis-CP[44].pdf]
