# Supplementary material for: Cyclo‐Polyproline: Chameleonic All‐Peptide Macrocycles With Induced‐Fit Host‐Guest Recognition
Source: Angew Chem Int Ed Engl. 2026 May 14;65(27):e8698780. doi: 10.1002/anie.8698780 (PMC13327576; doi:10.1002/anie.8698780)

checkCIF/PLATON report

Structure factors have been supplied for datablock(s) exp_2733_cdg-3-114_t2

THIS REPORT IS FOR GUIDANCE ONLY. IF USED AS PART OF A REVIEW PROCEDURE FOR PUBLICATION, IT SHOULD NOT REPLACE THE EXPERTISE OF AN EXPERIENCED CRYSTALLOGRAPHIC REFEREE.

No syntax errors found. CIF dictionary Interpreting this report

Datablock: exp_2733_cdg-3-114_t2

Bond precision: C-C = 0.0046 A Wavelength=1.54184

Cell:	a=23.802(2)	b=23.802(2)	c=10.4038(10)
	alpha=90	beta=90	gamma=90
Temperature:	108 K		

	Calculated	Reported
Volume	5894.1(11)	5894.1(11)
Space group	I -4	I -4
Hall group	I -4	I -4
Moiety formula	C80 H112 N16 O16, 4(O), 22(H2 O), 14(H O) [+ solvent]	C80 H112 N16 O16, 4(O), 22(H2 O), 13.362(H O), 4[H2O]
Sum formula	C80 H170 N16 O56 [+ solvent]	C80 H170 N16 O56
Mr	2252.32	2252.31
Dx,g cm-3	1.269	1.269
Z	2	2
Mu (mm-1)	0.919	0.919
F000	2420.0	2420.0
F000'	2429.02	
h,k,lmax	30,30,13	30,30,13
Nref	6214[3289]	5974
Tmin,Tmax	0.759,0.840	0.507,1.000
Tmin'	0.637	

Correction method= # Reported T Limits: Tmin=0.507 Tmax=1.000
AbsCorr = GAUSSIAN

Data completeness= 1.82/0.96 Theta(max)= 76.862

```
R(reflections)= 0.0438( 5511)                              wR2(reflections)=
                                                           0.1212( 5974)
S = 1.036                    Npar= 386
```

The following ALERTS were generated. Each ALERT has the format
test-name_ALERT_alert-type_alert-level.
Click on the hyperlinks for more details of the test.

Alert level B

```
PLAT306_ALERT_2_B Isolated Oxygen Atom (H-atoms Missing ?) .......         O5 Check
```

Author Response: Atom corresponds to a disordered water molecule in a symmetry axis.

```
PLAT306_ALERT_2_B Isolated Oxygen Atom (H-atoms Missing ?) .......         O9 Check
```

Author Response: Atom corresponds to a disordered water molecule in a symmetry axis.

Alert level C

```
PLAT260_ALERT_2_C Large Average Ueq of Residue Including       O17      0.122 Check
PLAT340_ALERT_3_C Low Bond Precision on  C-C Bonds ..............    0.00456 Ang.
PLAT417_ALERT_2_C Short Inter D-H..H-D       H7        ..H15      .       2.10 Ang.
                                                x,y,-1+z  =      1_554 Check
PLAT911_ALERT_3_C Missing FCF Refl Between Thmin & STh/L=    0.600          8 Report
                2  2  0,  -1  3  0,   1  3  0,   3  3  0,  -2  4  0,   2  4  0,
                7  7  0,   4 10  0,
```

Alert level G

```
FORMU01_ALERT_1_G  There is a discrepancy between the atom counts in the
            _chemical_formula_sum and _chemical_formula_moiety. This is
            usually due to the moiety formula being in the wrong format.
            Atom count from _chemical_formula_sum:   C80 H170 N16 O56
            Atom count from _chemical_formula_moiety:C80 H177.3619 N16 O59.36200
PLAT007_ALERT_5_G Number of Unrefined Donor-H Atoms .............         16 Report
              H8    H9    H13   H14   H1    H15   H18   H5    H3    H20   H12
              H6    H7    H10   H22   H4
PLAT042_ALERT_1_G Calc. and Reported MoietyFormula Strings  Differ     Please Check
              Calc: C80 H112 N16 O16, 4(O), 22(H2 O), 14(H O)
              Rep.: C80 H112 N16 O16, 4(O), 22(H2 O), 13.362(H O),
                     4[H2O]
PLAT187_ALERT_4_G The CIF-Embedded .res File Contains RIGU Records          1 Report
PLAT299_ALERT_4_G Atom Site Occupancy Constrained at ............        0.5 Check
              O12     H3      H20     O16     O17     H4
PLAT302_ALERT_4_G Anion/Solvent/Minor-Residue Disorder (Resd    9)       100% Note
PLAT302_ALERT_4_G Anion/Solvent/Minor-Residue Disorder (Resd   13)       100% Note
PLAT302_ALERT_4_G Anion/Solvent/Minor-Residue Disorder (Resd   14)       100% Note
PLAT304_ALERT_4_G Non-Integer Number of Atoms in ..... (Resd    2)       0.25 Check
PLAT304_ALERT_4_G Non-Integer Number of Atoms in ..... (Resd    6)       0.25 Check
PLAT304_ALERT_4_G Non-Integer Number of Atoms in ..... (Resd    9)       1.50 Check
```

```
PLAT304_ALERT_4_G Non-Integer Number of Atoms in ..... (Resd   13)       0.50 Check
PLAT311_ALERT_2_G Isolated Disordered Oxygen Atom (No H's ?) .....        O16 Check
PLAT415_ALERT_2_G Short Inter D-H..H-X       H3       ..H41      .       2.05 Ang.
                                              1-x,1-y,z  =      2_665 Check
PLAT417_ALERT_2_G Short Inter D-H..H-D       H1       ..H3       .       2.04 Ang.
                                                  x,y,z  =      1_555 Check
PLAT417_ALERT_2_G Short Inter D-H..H-D       H4       ..H5       .       1.95 Ang.
                                                  x,y,z  =      1_555 Check
PLAT605_ALERT_4_G Largest Solvent Accessible VOID in the Structure         78 A**3
PLAT792_ALERT_1_G Model has Chirality at C1          (Polar  SpGr)          S Verify
PLAT792_ALERT_1_G Model has Chirality at C6          (Polar  SpGr)          S Verify
PLAT792_ALERT_1_G Model has Chirality at C11         (Polar  SpGr)          R Verify
PLAT792_ALERT_1_G Model has Chirality at C16         (Polar  SpGr)          R Verify
PLAT868_ALERT_4_G ALERTS Due to the Use of _smtbx_masks Suppressed          ! Info
PLAT910_ALERT_3_G Missing FCF Reflection(s) Below Theta(Min)[Deg]=       4.64 Note
               1  1  0,   0  2  0,
PLAT912_ALERT_4_G Missing # of FCF Reflections Above STh/L=  0.600         25 Note
PLAT933_ALERT_2_G Number of HKL-OMIT Records in Embedded .res File          9 Note
              -2  4  0,  -1  3  0,   0  2  0,   1  3  0,   2  2  0,   2  4  0,
               3  3  0,   4 10  0,   7  7  0,
PLAT969_ALERT_5_G The 'Henn et al.' R-Factor-gap value ..........      5.315 Note
              Predicted wR2: Based on SigI**2  2.28 or SHELX Weight 11.69
PLAT978_ALERT_2_G Number C-C Bonds with Positive Residual Density.          1 Info
```

---

0 **ALERT level A** = Most likely a serious problem - resolve or explain
2 **ALERT level B** = A potentially serious problem, consider carefully
4 **ALERT level C** = Check. Ensure it is not caused by an omission or oversight
27 **ALERT level G** = General information/check it is not something unexpected

6 ALERT type 1 CIF construction/syntax error, inconsistent or missing data
10 ALERT type 2 Indicator that the structure model may be wrong or deficient
3 ALERT type 3 Indicator that the structure quality may be low
12 ALERT type 4 Improvement, methodology, query or suggestion
2 ALERT type 5 Informative message, check

It is advisable to attempt to resolve as many as possible of the alerts in all categories. Often the minor alerts point to easily fixed oversights, errors and omissions in your CIF or refinement strategy, so attention to these fine details can be worthwhile. In order to resolve some of the more serious problems it may be necessary to carry out additional measurements or structure refinements. However, the purpose of your study may justify the reported deviations and the more serious of these should normally be commented upon in the discussion or experimental section of a paper or in the "special_details" fields of the CIF. checkCIF was carefully designed to identify outliers and unusual parameters, but every test has its limitations and alerts that are not important in a particular case may appear. Conversely, the absence of alerts does not guarantee there are no aspects of the results needing attention. It is up to the individual to critically assess their own results and, if necessary, seek expert advice.

Publication of your CIF in IUCr journals

A basic structural check has been run on your CIF. These basic checks will be run on all CIFs submitted for publication in IUCr journals (*Acta Crystallographica*, *Journal of Applied Crystallography*, *Journal of Synchrotron Radiation*); however, if you intend to submit to *Acta Crystallographica Section C* or *E* or *IUCrData*, you should make sure that full publication checks are run on the final version of your CIF prior to submission.

Publication of your CIF in other journals

Please refer to the *Notes for Authors* of the relevant journal for any special instructions relating to CIF submission.

PLATON version of 04/06/2025; check.def file version of 30/05/2025

Datablock exp_2733_cdg-3-114_t2 - ellipsoid plot

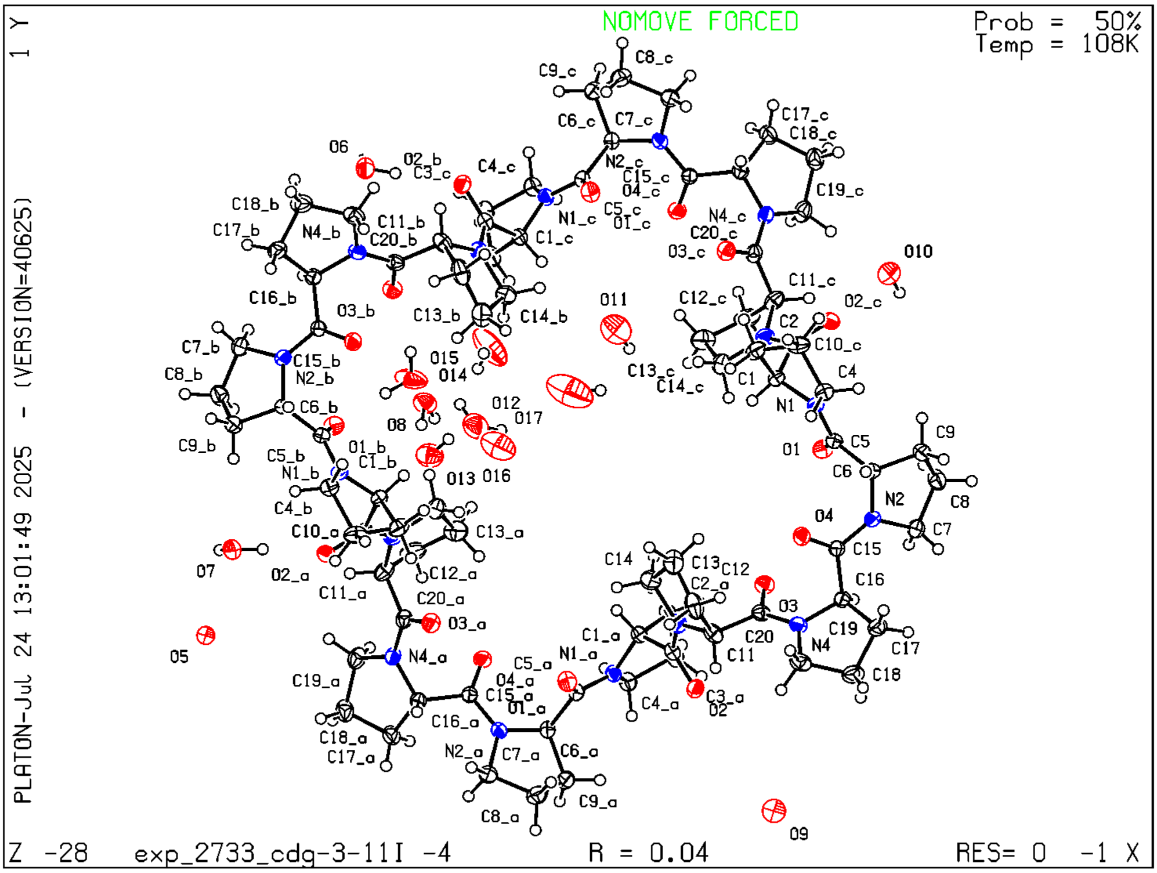

Supplement: Supplementary file 2 — Supporting File 2: anie72664‐sup‐0002‐Data.zip. [file ANIE-65-e8698780-s001.zip › anie72664-sup-0002-Data/checkcif-all-trans-CP[44].pdf]
